# Supplementary material for: Retinal chromophore charge delocalization and confinement explain the extreme photophysics of Neorhodopsin
Source: Nat Commun. 2022 Nov 4;13:6652. doi: 10.1038/s41467-022-33953-y (PMC9636224; doi:10.1038/s41467-022-33953-y)
Supplement: Supplementary file 1 — Supplementary Informations [file 41467_2022_33953_MOESM1_ESM.pdf]

Supplementary information for:

Retinal chromophore charge delocalization and confinement explain the extreme photophysics of Neorhodopsin

Riccardo Palombo [1,2], Leonardo Barneschi[1] Laura Pedraza-González[1], Daniele Padula[1], Igor Schapiro[3] and Massimo Olivucci[1,2]

[1] *Dipartimento di Biotecnologie, Chimica e Farmacia, Università di Siena, via A. Moro 2, I-53100 Siena, Siena, Italy.*
[2] *Department of Chemistry, Bowling Green State University, Bowling Green, Ohio 43403*, *United*
[3] *Fritz Haber Center for Molecular Dynamics, Institute of Chemistry, The Hebrew University of Jerusalem, 9190401 Jerusalem, Israel.*

Correspondence to: olivucci@unisi.it, molivuc@bgsu.edu

This document includes:

S1 a-ARM protocol 2
S2 Absorption and emission calculation 4
S3 Absorption band reproduction 9
S4 Mayer bond order analysis and electron density variations 10
S5 Minimum energy paths 12
References 27

Supplementary section 1: *a* -ARM protocol

All the hybrid quantum-mechanics / molecular-mechanics (QM/MM) models of NeoR reported in this work were generated using the *a*-ARM protocol which has been developed in the author lab [1,2]. *a*-ARM allows the construction of basic and gas-phase models aimed to the rationalization and prediction of trends between sequence variability and function. In fact, the design principle beyond *a*-ARM is the production of basic representations of complex protein systems which reproduce trends in photophysical and photochemical properties. Below, we a provide a schematic overview of the *a*-ARM protocol, while we redirect to the corresponding references for a complete and more exhaustive description of the methodology.

Each QM/MM model is divided into three subsystems (see Supplementary Figure 1) described below:

- **QM atoms.** They are the retinal protonated Schiff base (rPSB) cromophore atoms, the lysine linker (K211) side chain atoms starting from the N-terminal to the Cε and the hydrogen link atom (HLA).
- **MM cavity atoms.** They are the sidechain atoms of the residues forming the cavity, the water molecule atoms within 4 Å from the rPSB chromophore and the lysine linker atoms not included in the QM subsystem.
- **MM environment atoms.** They are all the remaining protein atoms, water atoms and ions excluded from the previous subsystems.

As shown in Supplementary Figure 1 (in gray), the MM environment atoms are kept frozen at the crystallographic (or homology model) position while QM and MM cavity atoms are free to relax. The *a*-ARM protocol is divided in two sequential phases:

1. **Input generation**. It identifies the three subsystems described above and takes as only input a Protein Data Bank (PDB) file corresponding either to a crystallographic structure or, as in this case of NeoR[3], to a homology structure. The protocol identifies the rPSB chromophore, the lysine linker (K211), the residues constituting the cavity and then it allows for the assignment of protonation states which can be automatic or, as in this work, can be manual. The system is then neutralized by adding Na^+ and Cl^- counterions at the intracellular (IS) or extracellular (OS) side of the protein.

2. **QM/MM Model generation.** It starts by adding hydrogen atoms to the whole protein and optimizing their position. Then, QM atoms and MM cavity atoms are equilibrated via an MM energy minimization. Then, 10 independent molecular dynamics (MD) simulations of 1 ns at the MM level using AMBER94 force field[4] are carried out from 10 different seeds. Once the MDs are terminated, the HLA is added at the QM/MM frontier and the generated 10 models are refined through a series of S_0 geometry optimizations featuring an increased level of theory: from HF/3-21G/AMBER94 to single state CASSCF(12,12)/6-31G*/AMBER94. Finally, these models are used to evaluate 10 vertical excitation energies ($\Delta E^{a,ARM}{}_{S0\text{-}S1}$) between S_0 and S_1 at the 3 root state-average CASPT2/CASSCF(12,12)/6-31G*/AMBER94 level of theory.

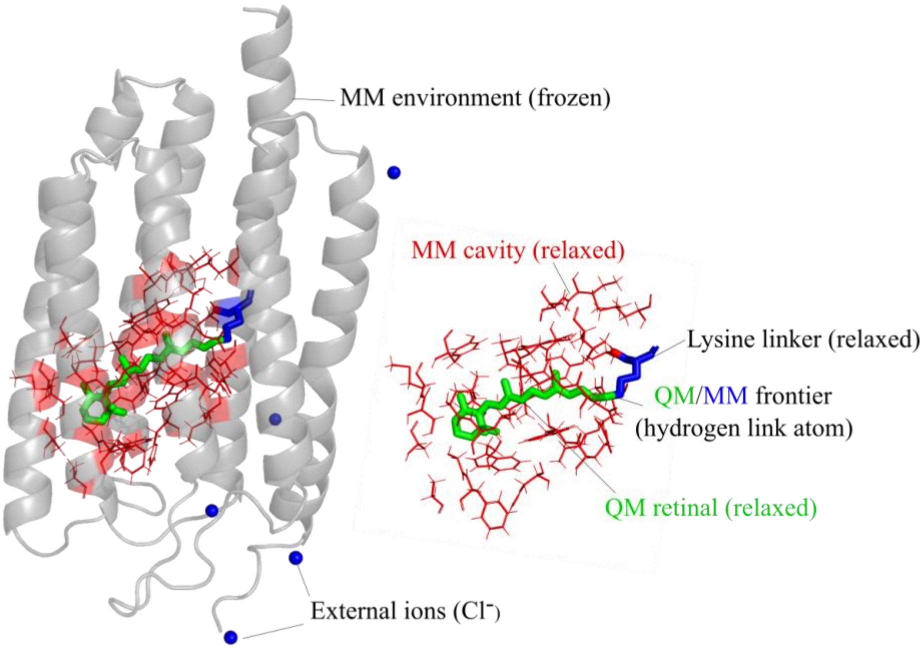

Supplementary Figure 1. Schematic overview of the *a*-ARM model of NeoR. The protein environment (frozen at the homology model position) is in gray while the sidechains of the cavity residues in red. Lysine linker - rPSB chromophore system is colored in blue - green, respectively, to reflect the corresponding QM/MM partitions. Blue spheres indicate the external counterions (Cl^-). Hydrogens atom of the rPSB chromophore were hidden for image clarity.

Supplementary section 2: Absorption and emission computation

Using the *a*-ARM protocol, we have systematically built possible protonation state configurations among E136, D140, E141 and E262 residues, which were identified as a possible candidates for the role of rPSB counterion in NeoR (see Supplementary Figure 2). As stated in the main text, this set of QM/MM models was ranked by computing maximum absorption wavelenght (λ^a_{max}), maximum emission wavelenght (λ^f_{max}) the reorganization energy (E^r) values schematically shown in Figure 1A. Here, we define the procedure adopted to compute these quantities for each choice of rPSB counterion. After the construction of the 10 *a*-ARM replicas and the calculation of the corresponding $\Delta E^{a,ARM}_{S0\text{-}S1}$ values (see Supplementary Section 1), we select the replica with the ($\Delta E^{a,ARM}_{S0\text{-}S1}$) value closest to the average. This replica is then reoptimized at the 2 root state average CASSCF(12,12)/6-31G*/AMBER94 level of theory on S_0 and on S_1 (notice that the replicas generated by *a*-ARM are optimized using a *single state* approach). The S_0 optimization provides the S_0 minimum energy structure of the Dark Adapted State (DA) while the S_1 optimization provides the S_1 minimum energy structure of the Fluorescent State (FS). Then, λ^a_{max} and λ^f_{max} (and also E^r) are obtained evaluating vertical excitation energies between S_0 and S_1 ($\Delta E_{S0\text{–}S1}$) at the DA and at the FS, respectively, at the 3 root state average CASPT2/CASSCF(12,12)/6-31G*/AMBER94 level of theory. The corresponding absorption and emission results are reported in Supplementary Tables 1 and 2, respectively.

As shown in Figure 2B we found that the model with no counterions (i.e. with all E136, D140, E141 and E262 residues protonated) does not exhibits a stable S_1 minimum and therefore, given the absence of a minimum on S_1, the energy gradient controlling the S_1

optimization steered the rPSB directly to the CoIn that was found associated to a nearly 90° twisted conformation along the C12-C13 bond.

Supplementary Table 1. Absorption data of NeoR models with varying rPSB counterions. Vertical energy differences ΔE_{S0}–S_1 (kcal mol^{-1} and eV in parentheses), oscillator strengths f and electronic transition moment μ (Debye, D) are reported for the three transitions among S_0, S_1 and S_2 electronic states.

Counterion ID	ΔE_{S0-S1}	ΔE_{S0-S2}	ΔE_{S1-S2}	f_{S0-S1}	f_{S0-S2}	f_{S1-S2}	μ_{S0-S1}	μ_{S0-S2}	μ_{S1-S2}
No counterion	42.17 (1.83)	60.41 (2.62)	18.24 (0.79)	0.83	0.41	0.07	4.31	2.51	1.86
E136	49.17 (2.13)	75.16 (3.26)	26.00 (1.13)	0.72	0.38	0.19	3.72	2.18	2.64
D140	50.34 (2.18)	70.08 (3.04)	19.75 (0.86)	0.65	0.49	0.27	3.48	2.57	3.61
E141	44.40 (1.93)	63.59 (2.76)	19.18 (0.83)	1.71	0.09	0.02	6.01	1.14	1.08
E262	47.46 (2.06)	75.69 (3.28)	28.24 (1.22)	0.79	0.30	0.11	3.97	1.92	1.95
E136 D140	78.58 (3.41)	90.03 (3.90)	11.46 (0.50)	0.75	0.26	0.01	3.01	1.65	0.63
E136 E141	53.05 (2.30)	76.10 (3.30)	22.96 (0.99)	0.91	0.31	0.07	4.02	1.96	1.74
E136 E262	65.08 (2.82)	74.02 (3.20)	8.94 (0.39)	0.73	0.33	0.12	3.26	2.19	3.50
E141 E262	52.42 (2.27)	76.12 (3.30)	23.70 (1.02)	0.94	0.27	0.04	4.11	1.81	1.33
D140 E141	54.74 (2.37)	78.12 (3.39)	23.38 (1.01)	0.92	0.28	0.04	3.98	1.83	1.39
D140 E262	66.39 (2.88)	71.17 (3.09)	4.77 (0.20)	0.75	0.24	0.05	3.26	1.77	3.18
E136 D140 E141	73.76 (3.19)	71.16 (3.09)	2.60 (0.11)	0.97	0.13	0.02	3.58	1.27	2.50
E136 D140 E262	82.39 (3.57)	100.03 (4.33)	17.64 (0.76)	0.03	0.06	0.00	0.62	0.73	0.20
E136 E141 E262	72.61 (3.15)	72.05 (3.12)	0.56 (0.02)	0.15	0.92	0.00	1.39	3.46	2.53
E140 E141 E262	72.14 (3.13)	72.55 (3.15)	0.41 (0.01)	0.18	0.93	0.00	1.53	3.48	2.75

Supplementary Table 2. Emission data of NeoR models with varying rPSB counterions. Vertical energy differences ΔE (kcal mol^{-1} and eV in parentheses), oscillator strenghts f and electronic transition moment μ (Debye, D) are reported for the three electronic transition among S_0, S_1 and S_2 electronic states. NP entry indicates that the model does not exhibit a S_1 minimum energy geometry (i.e. the FS).

Counterion ID	ΔE_{S0-S1}	ΔE_{S0-S2}	ΔE_{S1-S2}	f_{S0-S1}	f_{S0-S2}	f_{S1-S2}	μ_{S0-S1}	μ_{S0-S2}	μ_{S1-S2}
No counterion	NP	NP	NP	NP	NP	NP	NP	NP	NP
E136	32.05 (1.39)	53.18 (2.30)	21.13 (0.92)	1.17	0.46	0.35	5.86	2.84	3.96
D140	31.84 (1.38)	53.23 (2.30)	21.34 (0.93)	1.15	0.45	0.37	5.83	2.82	4.02
E141	41.27 (1.79)	57.58 (2.50)	16.31 (0.71)	1.90	0.03	0.00	6.57	0.75	0.38
E262	32.05 (1.39)	53.90 (2.34)	21.85 (0.95)	1.29	0.42	0.29	6.15	2.70	3.56
E136 D140	47.93 (2.08)	74.97 (3.25)	27.03 (1.17)	0.58	0.29	0.03	3.38	1.90	1.01
E136 E141	35.08 (1.52)	54.37 (2.36)	19.34 (0.83)	1.08	0.51	0.30	5.39	2.97	3.79
E136 E262	48.00 (2.08)	72.22 (3.13)	24.22 (1.05)	0.49	0.00	0.00	3.10	0.02	0.00
E141 E262	34.31 (1.49)	54.52 (2.36)	20.21 (0.88)	1.08	0.47	0.28	5.46	2.85	3.60
D140 E141	36.01 (1.56)	54.66 (2.37)	18.71 (0.81)	1.11	0.49	0.26	5.39	2.91	3.58
D140 E262	42.13 (1.83)	66.99 (2.90)	24.85 (1.08)	0.44	0.12	0.00	3.15	1.32	0.00
E136 D140 E141	47.42 (2.06)	73.60 (3.19)	26.18 (1.14)	0.00	0.00	0.00	0.04	0.07	0.00
E136 D140 E262	42.42 (1.83)	71.12 (3.08)	28.70 (1.24)	0.00	0.00	0.00	0.20	0.20	0.10
E136 E141 E262	47.07 (2.04)	73.21 (3.17)	26.14 (1.13)	0.01	0.04	0.00	0.33	0.70	0.15
E140 E141 E262	47.91 (2.08)	73.98 (3.20)	26.07 (1.13)	0.00	0.03	0.00	0.35	0.67	0.19

By leveraging the capability of *a*-ARM to produce single or multiple point mutations, and using the procedure described above, we have computed λ^a_{max} values for a set of NeoR variants, and compared them with the experimentally observed counterparts[5]. As stated in the main text, these variants were generated from the *a*-ARM model featuring E141 as *r*PSB counterion (from now on simply ARM_{E141}). Clearly, E141C variant does not feature a *r*PSB counterion. Supplementary Table 3 shows the results in terms of ΔE_{S0-S1} values. We found a Mean Absolute Error (MAE) of 2.18 kcal mol^{-1} and a Mean Absolute Deviation (MAD) of 0.92 kcal mol^{-1}. This MAE value is below the MAE value of 3.0 kcal mol^{-1} that represents the error bar of the *a*-ARM technology. Furthermore, with the aim of quantifying the parallelism between the computed and the observed ΔE_{S0-S1} values, we determined the trend deviation factor ||Trend Dev.|| (see Supplementary Table 3 for definition). The corresponding MAE value is found to be 1.00 kcal mol^{-1}, well below the MAE value of ||Trend Dev.|| of 2.5 kcal mol^{-1} reported for the *a*-ARM protocol benchmark set that included 25 wild-type rhodopsins from vertebrate, invertebrate, and microbial organisms.

Supplementary Table 3. Comparison between computed and experimentally observed vertical excitation energies (ΔE_{S0-S1}) defining the λ^a_{max} values in the WT and a set of NeoR mutants. Differences between computed (ΔE^{Comp}_{S0-S1}) and experimental (ΔE^{Exp}_{S0-S1}) energies are shown ($\Delta E_{S0-S1}^{Comp-Exp}$). All data are in kcal mol^{-1}.

Mutant	ΔE^{Exp}_{S0-S1}	$\Delta_i^{Exp, (WT)\ a}$	ΔE^{Comp}_{S0-S1}	$\Delta_i^{Comp, (WT)\ b}$	$\Delta E_{S0-S1}^{Comp-Exp}$	|| Trend Dev. ||[c]
WT	41.44		44.40	0	2.97	0
E141C	41.02	0.42	41.31	3.10	0.29	2.68
D140N	41.68	-0.24	45.00	-0.59	3.32	0.35
D140C	42.30	-0.86	45.03	-0.62	2.73	0.24
D140T	45.38	-3.94	46.87	-2.46	1.49	1.48
T238A	43.06	-1.62	45.45	-1.04	2.39	0.58
T238P	43.19	-1.75	45.53	-1.12	2.34	0.63
W234F	41.25	0.19	43.91	0.50	2.66	0.31
W241H	41.68	-0.24	45.24	-0.83	3.56	0.59
W110Y	42.23	-0.79	45.10	-0.69	2.87	0.10
S191A	42.36	-0.92	43.58	0.83	1.22	1.75
T238A S191A	45.74	-4.30	45.38	-0.97	-0.34	3.33
MAE ± MAD of $\Delta E_{S0-S1}^{Comp-Exp}$					2.18 ± 0.92	
MAE ± MAD of || Trend Dev. ||						1.00 ± 0.62

[a] Difference between the experimental ΔE^{Exp}_{S0-S1} of WT and of the i-th mutant

[b] Difference between the computed ΔE^{Comp}_{S0-S1} of WT and of the i-th mutant

|| Trend Dev. || = | $\Delta_i^{Exp, (WT)}$ - $\Delta_i^{Comp, (WT)}$ |

In order to determine whether the differences in the absorption and emission vertican excitation energies originate from a geometrical effect of the rPSB chromophore or from specific interactions between the QM and MM subsystems (or a combination of the two), we compare the reported excitation energy trends of the full protein models ($\Delta E^{protein}_{S0-S1}$ values) with the corresponding trends computed vacuo (ΔE^{vacuo}_{S0-S1} values) after extracting the rPSB chromophore geometry from its protein environment and without re-optimizing its geometry. Also, we use the excitation energy of the selected a-ARM$_{E141}$ model as a reference to quantify the extent of the trend variations. As reported in Supplementary Table 4, increasing the net total negative charge of the counterion tetrad (from 0 to -3) results in a general blue shift in absorption: from the excitation energy value of 42.17 kcal mol^{-1} displayed by the model with no counterions to excitation energy values > 70 kcal mol^{-1} displayed by the models with 3 counterions. This is translated in energy variations ranging from ca -2 kcal mol^{-1} (model with no counterion) to ca 38 kcal mol^{-1} (model featuring E136 D140 E262 counterions) with respect to a-ARM$_{E141}$. Such behavior is strongly attenuated or even lost in vacuo where no more than ca 4 kcal mol^{-1} (model featuring E136 E262 counterions) of variation is computed. This behavior is also found in the emission trend (Supplementary Table 5). In conclusion, the main contribution to the computed differences in vertical excitation energies among models displaying different protonation states, originates from the interaction between the chromophore (the QM subsystem) with the surrounding protein environment (the MM subsystem) displaying different protonation state configurations.

Supplementary Table 4. Vertical energy differences (at the CASPT2/AMBER level of theory) for different counterion choices between S_0 and S_1 at the DA computed in protein ($\Delta E^{protein}_{S0-S1}$) and

in vacuo (ΔE^{vacuo}_{S0-S1}). Values in parenthesis show the differences from the QM/MM model featuring E141 as rPSB counterion.

**Counterion ID**	$\Delta E^{protein}_{S0-S1}$	ΔE^{vacuo}_{S0-S1}
No counterion	42.17 (-2.23)	41.98 (1.72)
E136	49.17 (4.77)	45.47 (1.77)
D140	50.34 (5.94)	44.80 (1.1)
E141	44.40 (0)	43.70 (0)
E262	47.46 (3.06)	45.81 (2.11)
E136 D140	78.58 (34.18)	47.22 (3.52)
E136 E141	53.05 (8.65)	45.67 (1.97)
E136 E262	65.08 (20.68)	47.68 (3.98)
E141 E262	52.42 (8.02)	45.15 (1.45)
D140 E141	54.74 (10.34)	45.78 (2.08)
D140 E262	66.39 (21.99)	45.59 (1.89)
E136 D140 E141	73.76 (29.36)	46.38 (2.68)
E136 D140 E262	82.39 (37.99)	47.49 (3.79)
E136 E141 E262	72.61 (28.21)	46.10 (2.4)
E140 E141 E262	72.14 (27.74)	46.07 (2.37)

Supplementary Table 5. Vertical energy differences (at the CASPT2/AMBER level of theory) for different counterion choices between S_0 and S_1 at the FS computed in protein ($\Delta E^{protein}_{S0-S1}$) and in vacuo ($\Delta E^{vacuo}_{S0-S1}$). Values in parenthesis show the differences from the QM/MM model featuring E141 as rPSB counterion.

Counterion ID	$\Delta E^{protein}_{S0-S1}$	ΔE^{vacuo}_{S0-S1}
E136	32.05 (-9.22)	38.59 (-1.76)
D140	31.84 (-9.43)	37.69 (-2.66)
E141	41.27 (0)	40.35 (0)
E262	32.05 (-9.22)	39.00 (-1.35)
E136 D140	47.93 (6.66)	38.51 (-1.84)
E136 E141	35.08 (-6.19)	36.95 (-3.40)
E136 E262	48.00 (6.73)	38.34 (-2.01)
E141 E262	34.31 (-6.96)	36.51 (-3.84)
D140 E141	36.01 (-5.26)	35.59 (-4.76)
D140 E262	42.13 (0.86)	37.43 (-2.92)
E136 D140 E141	47.42 (6.15)	37.67 (-2.68)
E136 D140 E262	42.42 (1.15)	36.11 (-4.24)
E136 E141 E262	47.07 (5.8)	37.90 (-2.45)
E140 E141 E262	47.91 (6.68)	38.01 (-2.34)

Supplementary section 3: Absorption band simulation

The *a*-ARM$_{E141}$ model was used to simulate the experimentally observed absorption band of NeoR (see Figure 2B). The computed band was generated from a set of 200 initial conditions which we assume to represent the ground state room temperature Boltzman-like distribution of NeoR. The following sequential protocol was adopted[6,7] for the production of the initial conditions: (i) Room temperature MD of 20 ns using AMBER94

force field with GROMACS. This simulation consists of 50 ps of heating phase, of 150 ps of equilibration phase (at room temperature) and of 19800 ps of production phase with time interval of 1 fs. (ii) Geometries and velocities corresponding to 200 indipendent snapshots were extracted from the production phase every every 100 ps. (iii) Each snapshot was then used to to propagate a 200 fs trajectory at the HF/3-21G/AMBER94 level of theory. The final geometry and velocity vectors were taken as intermediate initial conditions for running the subsequent 50 fs trajectories on S_0 at 2 root state average CASSCF(12,12)/6-31G*/AMBER94 level of theory. Then, the collection of geometries and velocities from the last snapshots were taken as the final set of initial conditions. Then, a stick spectra was generated by computing ΔE_{S0-S1} values at the 3 root state average CASPT2/CASSCF(12,12)/6-31G*/AMBER94 level of theory from the geometries of the initial conditions. Finally, the band was generated by convoluting the stick spectra using a Gaussian function using the oscillator strenghts (f_{S0-S1}) to weight the intensity of each $S_0 \rightarrow S_1$ transition.

Supplementary section 4: Bond order analysis and electron density variations upon absorption and emission

Supplementary Tables 6 and 7 show the results of the Mayer bond order analysis of the rPSB chromophore based on the 3 root state average CASPT2/CASSCF(12,12)/6-31G*/AMBER94 electron densities of the states (S_0 and S_1) involved in the two vertical transitions defining the computed absorption and emission. The analysis was performed using the Multiwfn[8] toolkit. We have repeated the analysis either for absorption and emission of the model with E141 as rPSB couterion (a-ARM$_{E141}$) either for absorption of the model with E262 (a-ARM$_{E262}$) as rPSB counterions. The analysis of the bond orders was used to derive resonance formulas in Figure 3B, Figure 3C and in Supplementary Figure 2. The resulting bond orders provide an elemental and basic representation of the electron density variation involving the π-system of the *r*PSB chromophore upon the two vertical transitions. The results are therefore qualitative but assumed to be accurate

enough to establish mechanistic diversities between ARM_{E141} and ARM_{E262} models. To further support the qualitative distribution of the positive charge derived by the resonance formulas, we provide in Figure 3A, Figure 3B and in Supplementary Figure 2 the positive charge (Mulliken) distribution in the rPSB chromophore using the bubble representations. The charge values are derived from S_0 and S_1 3 root state average CASPT2/CASSCF(12,12)/6-31G*/AMBER94 electron densities.

Supplementary Table 6. Bond order along the rPSB π-backbone in the a-ARM_{E141} model . Bond orders were derived from Mayer bond order analysis based on S_0 and S_1 3 root state average CASPT2/CASSCF(12,12)/6-31G*/AMBER94 electron densities of the DA, FC, FS and the vertical S_0 state corresponding to the FS geometry (see Fig. 1A).

a-ARM_{E141}				
**Bond**	**DA**	**FC**	**FS**	**S_0**
C5-C6	1.68	1.58	1.54	1.61
C6-C7	1.10	1.17	1.19	1.16
C7-C8	1.61	1.45	1.41	1.51
C8-C9	1.15	1.25	1.26	1.19
C9-C10	1.45	1.37	1.31	1.41
C10-C11	1.26	1.26	1.26	1.30
C11-C12	1.40	1.33	1.31	1.37
C12-C13	1.33	1.25	1.26	1.36
C13-C14	1.30	1.26	1.27	1.27
C14-C15	1.47	1.41	1.43	1.49
C15-N	1.15	1.10	1.10	1.12

Supplementary Table 7. Bond orders along the rPSB π-backbone in the ARM_{E262} model. Bond orders were derived from Mayer bond order analysis based on S_0 and S_1 3 root state average CASPT2/CASSCF(12,12)/6-31G*/AMBER94 electron densities of the DA, and FC.

a-ARM_{E262}		
**Bond**	**DA**	**FC**
C5-C6	1.71	1.60
C6-C7	1.07	1.16
C7-C8	1.65	1.53
C8-C9	1.11	1.19
C9-C10	1.58	1.37
C10-C11	1.14	1.22
C11-C12	1.57	1.40

C12-C13	1.16	1.21
C13-C14	1.51	1.31
C14-C15	1.24	1.33
C15-N	1.50	1.17

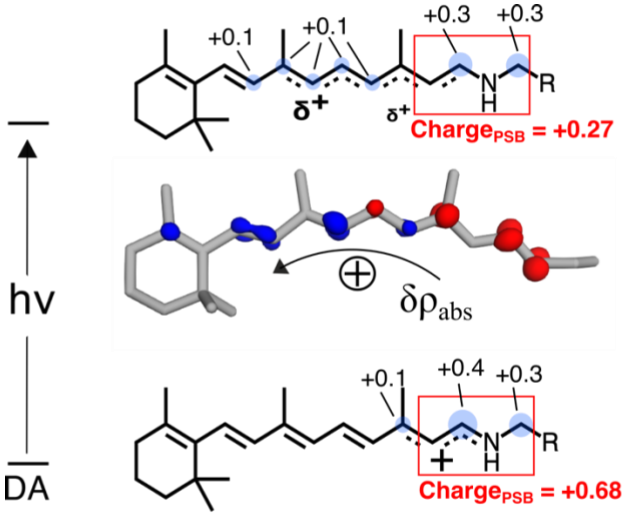

Supplementary Figure 2. Electronic character of the Dark Adapted State (DA) of the NeoR model featuring E262 residue as rPSB counterion (*a*-ARM$_{E262}$). A) Electron density variations between S_0 and S_1 states for the absorption ($\delta\rho_{abs}$). The blue and red clouds correspond to the zones of electron density decrease and increase, respectively. Isovalues set to 0.002 a.u. Resonance formulas are derived from Mayer Bond order analysis (see table S7). Blue bubbles represent the QM positive charge (in e unites). Only absolute values > 0.05 e are reported. As indicated by the red box, the total charge residing in the -C14-C15-N-Cε- rPSB fragment is also reported.

Supplementary section 5: Minimum energy paths

The computation of the minimum energy paths (MEPs) for the rPSB photoisomerization was performed through a serie of constrained geometry optimizations (relaxed scans) along the dihedral angles describing the photoisomerization reactions along C13=C14, C11=C12, C9=C10 and C7=C8 double bonds. As schematically shown in Figure 1A, each

path starts from the S_1 relaxation to the FS and then proceed through the progressive rotation of the selected dihedral angle of 5 degrees. According to the International Union of Pure and Applied Chemistry (IUPAC), the direction of rotation was chosen counterclockwise (CCW) for the C11=C12 and C7=C8 double bonds and clockwise (CW) for the C13=C14 and C9=C10 double bonds. Hence, in the CW rotation the dihedral angle varies from -180 degrees to 0 degree while in the CCW rotation the dihedral angle goes from 0 to -180 degrees. Then, in order to locate the geometries of the four Conical Intersections (CoIn), we carried out a geometry optimization without geometrical constrains, but using the energetical constrain of the energy difference between S_0 and S_1 equal to zero. Since the geometry optimization at the CASPT2 level is not feasible using our Molcas-based technology[9], the series of geometries defining each MEP was collected through a geometry optimization with the necessary S_1 energy gradients calculated at the 2 root state average CASSCF(12,12)/6-31G*/AMBER94 level of theory. However, to account for the missing dynamic correlation effect of the CASSCF method, we have re-evaluated (i.e. single point calculation) the energies of the CASSCF optimized geometries via the more computationally expensive 3 root state average CASPT2/CASSCF(12,12)/6-31G*/AMBER94 level of theory (CASPT2/AMBER). To verify the quality of the CASPT2 energy profiles we also re-performed the analysis with the more robust the 3 root state average XMCQDPT2/CASSCF(12,12)/AMBER94 level of theory method implemented in Firefly v8.2[10]. Supplementary Figure 5 shows the corresponding energy profiles.

The choice of the CW rotation of C13=C14 is consistent with the *ca.* +10 degree pre-twisting seen in both the DA and the FS of the selected model. The choice is based on the observed fact[11–17] that, in both microbial and animal rhodopsins, the pre-twisting imposed by steric and electrostatic cavity effects, bias the rPSB isomerization in the CW or CCW directions. A past study on the CW and CCW C13=C14 isomerization direction in the chimeric C1C2 channelrhodopsin, showed that the CCW path displays a larger energy barrier[18,19] consistently with a *ca* +7 degrees CW pre-twisting. In order to exclude

the possibility of a favourable CCW C13=C14 isomerization path in NeoR, in Supplementary Figure 4 we compare the initial S_1 energy profiles in the CW and CCW directions. As stated above, the pre-twisting of ca. +10 degrees displayed at the FC (i.e. at the DA geometry, see Figure 1A) is conserved after the small S_1 relaxation (orange curly arrow) to FS. From FS, the CCW rotation (i.e. opposite to the direction of pre-twisting) gives rise to a steeper S_1 profile (see Supplementary Figure 4).

The energy trends emerged from the less computationally expensive 2 root state average CASSCF(12,12)/6-31G*/AMBER94 approach are confirmed after both corrections. In fact, from the FS both CASPT2 and XMCQDPT2 corrections indicate that the S_1 energy increases monotonically untill the CoIn's geometries which then correspond to the highest points along each MEP. Consequently, we estimated the S_1 energy barrier (E^f_{S1}) for the double bond photoisomerization as the difference between the CoIn (highest point) and the FS (lowest point) S_1 energy values. Thus, we found 21, 17, 16 and 20 kcal mol^{-1} CASPT2 energy barriers, and 25, 22 ,16 and 16 kcal mol^{-1} XMCQDPT2 energy barriers, for respectively C13=C14, C11=C12, C9=C10 and C7=C8 double bond photoisomerizations. It is evident that after the energy corrections, the S_0/S_1 degeneracy of the CoIn's is lost in all four MEPs. This is not a unexpected since the geometries were optimized at different level and, also, its is know [20–24] that multistate multiconfigurational level approaches suffers from some artifacts when it comes to the description of the region approaching and surrounding the CoIn.

It is worth mentioning that, Marin et al found in a blue-shifted mutant of the *Anabaena Sensory Rhodopsin* (ASR) that a near-degeneracy situation of the S_1 and S_2 states was likely to be the origin of a barrier along the S_1 isomerization path[25]. In contrast, CASSCF, CASPT2 and XMCQDPT2 calculations have showed that S_1 and S_2 are far from being degenerate (more than 15 kcal mol^{-1} energy spacing along the entire paths), and so S_2 appears not be involved in its fluorescent mechanism of NeoR. For this reason, and in line with the works carried out on GFPs[26,27], in the main text we discuss the fluorescent mechanism by referring exclusively to the S_0 and S_1 states.

In order to evaluate the electrostatic effect imposed by the full protein to the MEPs, we have recomputed the energy profiles after setting to zero the MM charges of the entire protein while keeping the rPSB geometries unchanged. Then, we have also evaluated the individual electrostatic effect imposed by the rPSB counterion by setting to zero only the MM charges of the E141 residue. Supplementary Figures 6 and 7 shows the corresponding energy profiles. Finally, Supplementary Figure 8 shows the energy profiles recomputed in absence of the whole protein (in vacuo) in a full QM calculation.

In addition to the MEPs along the rPSB double bonds we computed (see Supplementary Figures 9 and 10) the C8-C9, C10-C11 and C12-C13 single bond MEPs to make sure that there are energy barriers preventing the excited state isomerization along these coordinates. The resulting 21, 21, 41 and 23, 20, 33 kcal mol^{-1} CASPT2 energy barriers for CW and CCW rotations respectively support the stability of the FS of our a-ARM$_{E141}$ model. In conclusion, we found that the S_1 rotation of each chromophore bond comprised between C7 and C14 atoms of the NeoR is associated with a barrier greater than 15 kcal/mol^{-1} (see Supplementary Figures 5, 9 and 10).

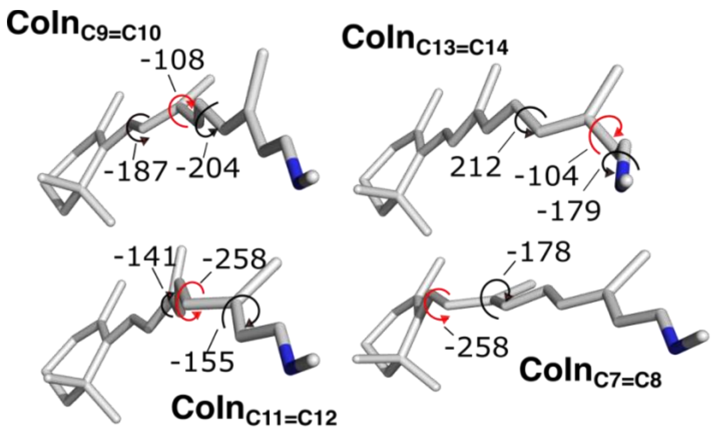

Supplementary Figure 3. Overview of the rPSB four CoIn's structures driving the S_1 isomerizations about the C13=C14, C11=C12, C9=C10 and C7=C8 double bonds. The curly arrows document the competing bicycle pedal torsional deformations.

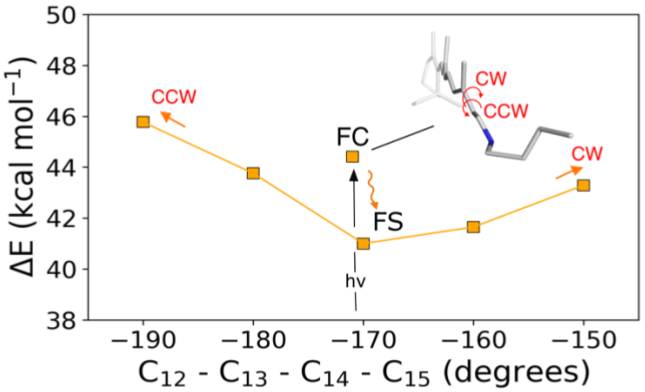

Supplementary Figure 4. Comparison between the S_1 energy profiles (CASPT2/AMBER level of theory) of the first two steps associated to the CW and CCW photoisomerization paths along the C13=C14 double bond. Orange curly arrow indicates the S_1 torsional relaxation from the Franck-Condon (FC) point.

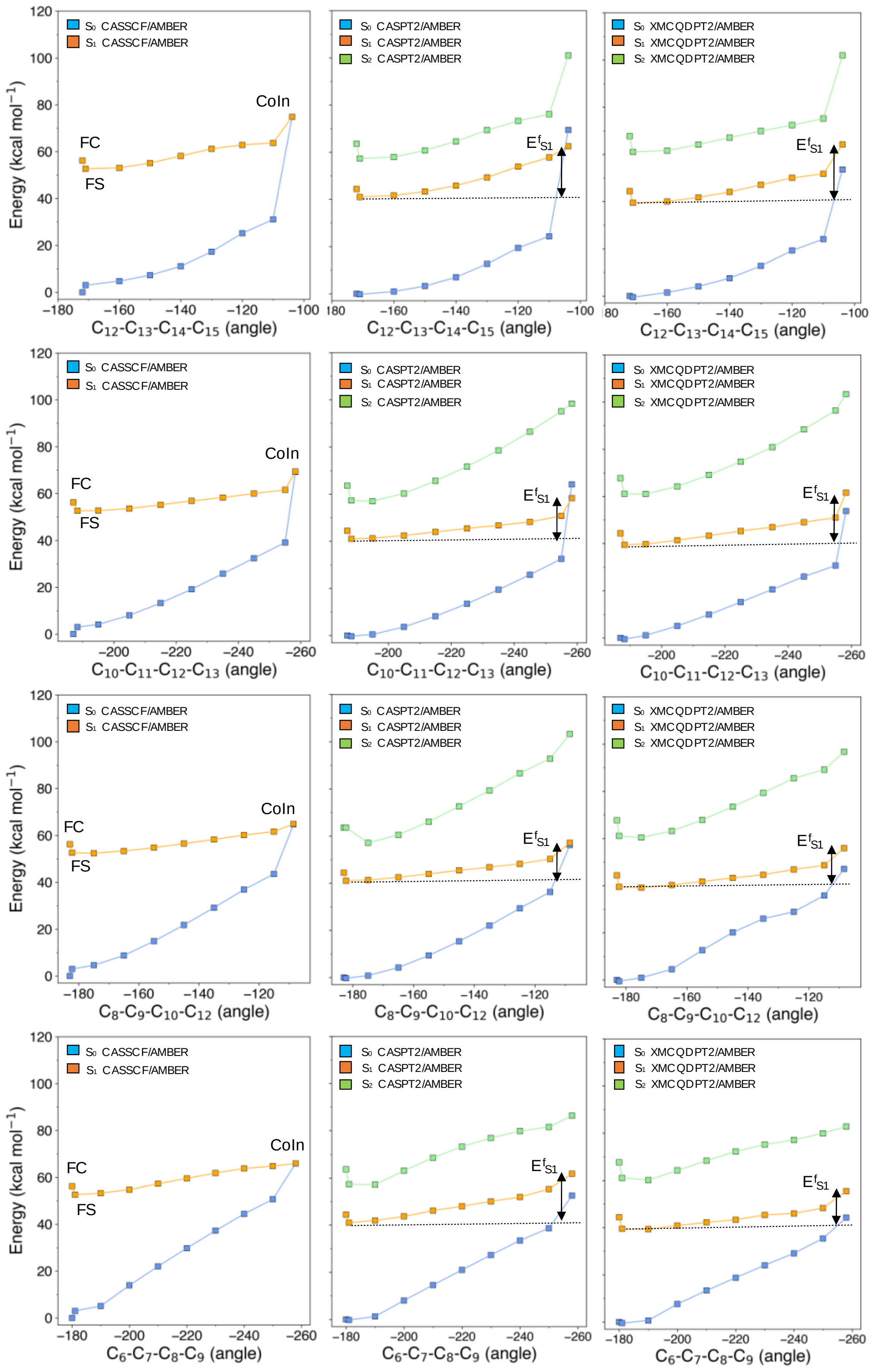
Energy (kcal mol−1)
S0 CASSCF/AMBER
S1 CASSCF/AMBER
S0 CASPT2/AMBER
S1 CASPT2/AMBER
S2 CASPT2/AMBER
S0 XMCQDPT2/AMBER
S1 XMCQDPT2/AMBER
S2 XMCQDPT2/AMBER
FC
FS
CoIn
E^f_{S1}
C12-C13-C14-C15 (angle)
C10-C11-C12-C13 (angle)
C8-C9-C10-C12 (angle)
C6-C7-C8-C9 (angle)

Supplementary Figure 5. Respectively from first row to bottom row: photoisomerization MEP along C13=C14, C11=C12, C9=C10 and C7=C8 rPSB double bonds. Energies are relative to the DA. For each MEPs, 2 root state average CASSCF(12,12)/6-31G*/AMBER94 (CASSCF/AMBER), 3 root state average CASPT2/CASSCF(12,12)/6-31G*/AMBER94 (CASPT2/AMBER) and 3 root state average XMCQDPT2/CASSCF(12,12)/6-31G*/AMBER94 (XMCQDPT2/AMBER) energy profiles are reported. E^{f}_{S1} indicates the S_1 isomerization barrier and corresponds to the CoIn and FS S_1 energy difference.

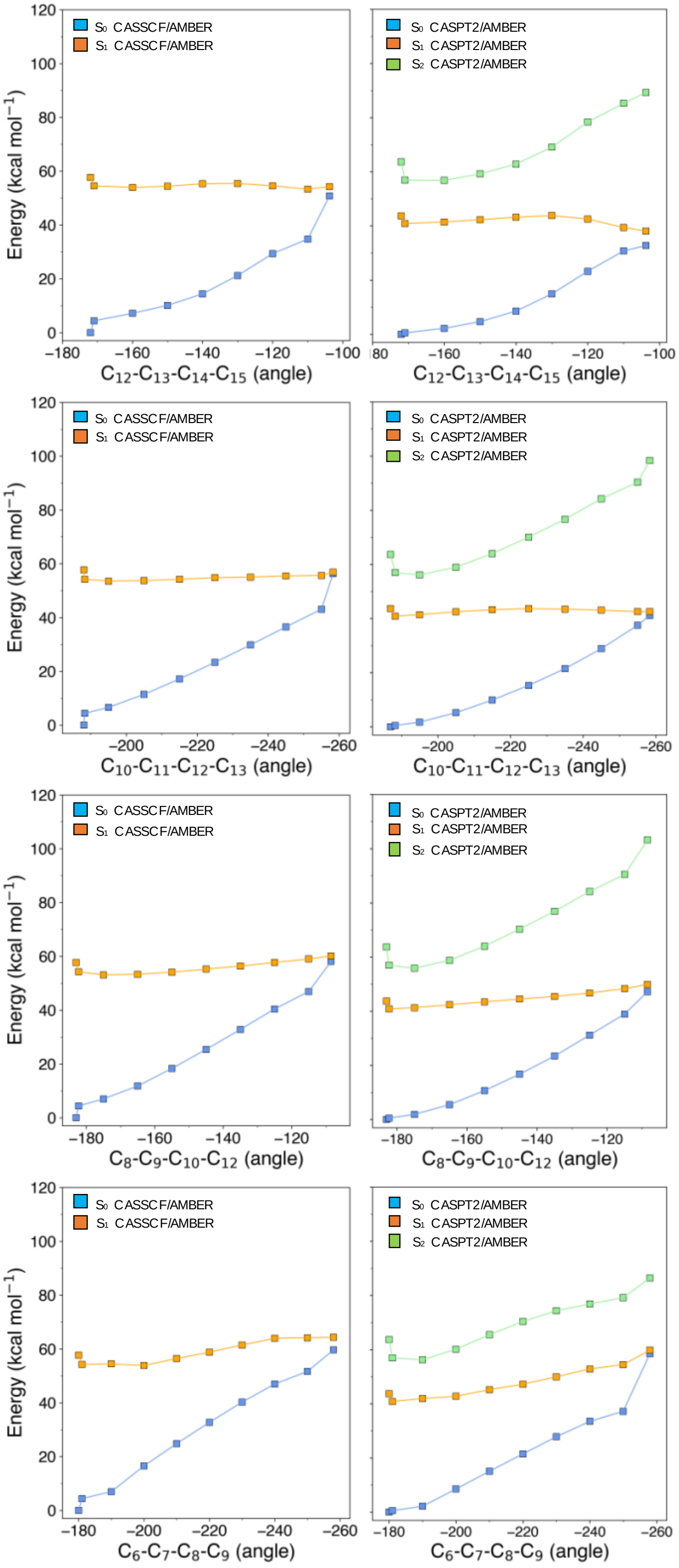

Supplementary Figure 6. Respectively from first row to bottom row: re-evaluated photoisomerization MEPs along C13=C14, C11=C12, C9=C10 and C7=C8 rPSB double bonds

after setting to zero the MM charges of the entire protein. Energies are relative to the DA. For each MEP, 2 root state average CASSCF(12,12)/6-31G*/AMBER94 (CASSCF/AMBER) and 3 root state average CASPT2/CASSCF(12,12)/6-31G*/AMBER94 (CASPT2/AMBER) energy profiles are reported.

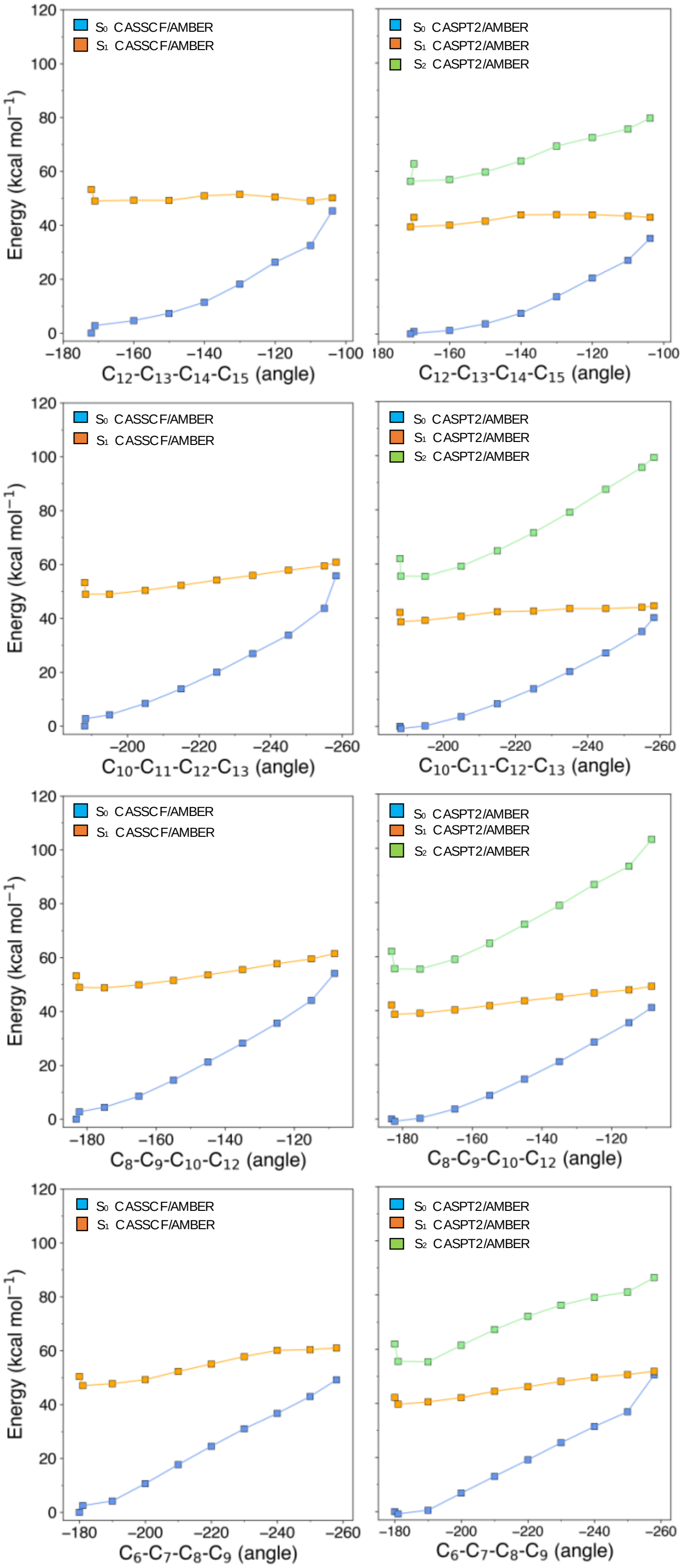

Supplementary Figure 7. Respectively from first row to bottom row: re-evaluated photoisomerization MEPs along C13=C14, C11=C12, C9=C10 and C7=C8 rPSB double bonds

after setting to zero the MM charges of the E141 residue (rPSB counterion). Energies are relative to the DA. For each MEP, 2 root state average CASSCF(12,12)/6-31G*/AMBER94 (CASSCF/AMBER) and 3 root state average CASPT2/CASSCF(12,12)/6-31G*/AMBER94 (CASPT2/AMBER) energy profiles are reported.

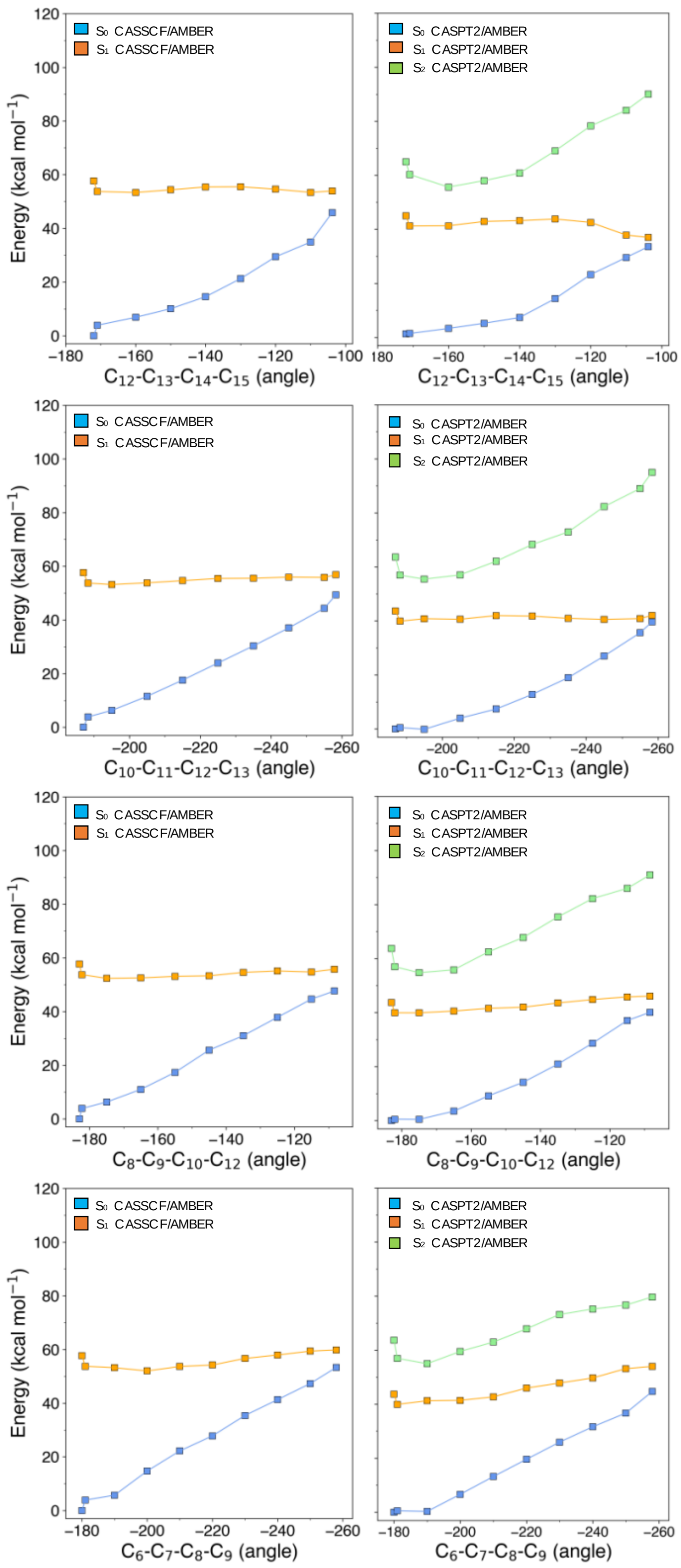

Supplementary Figure 8. Respectively from first row to bottom row: re-evaluated photoisomerization MEPs along C13=C14, C11=C12, C9=C10 and C7=C8 rPSB double bonds in the absence of the whole protein (in vacuo). Energies are relative to the DA. For each MEP, 2

root state average CASSCF(12,12)/6-31G*/AMBER94 (CASSCF/AMBER) and 3 root state average CASPT2/CASSCF(12,12)/6-31G*/AMBER94 (CASPT2/AMBER) energy profiles are reported.

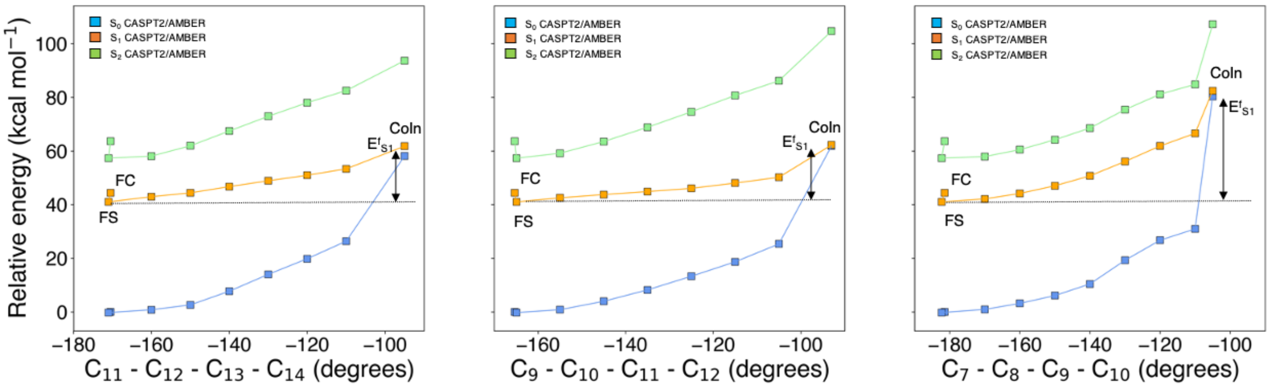

Supplementary Figure 9. Respectively from left to right: photoisomerization MEP associated with the CW rotations of C12-C13, C10-C11, C8-C9 rPSB single bonds. Energies are relative to the DA. For each MEPs, 3 root state average CASPT2/CASSCF(12,12)/6-31G*/AMBER94 (CASPT2/AMBER) energy profiles are reported. E^f_{S1} indicates the S_1 isomerization barrier and corresponds to the CoIn and FS S_1 energy difference.

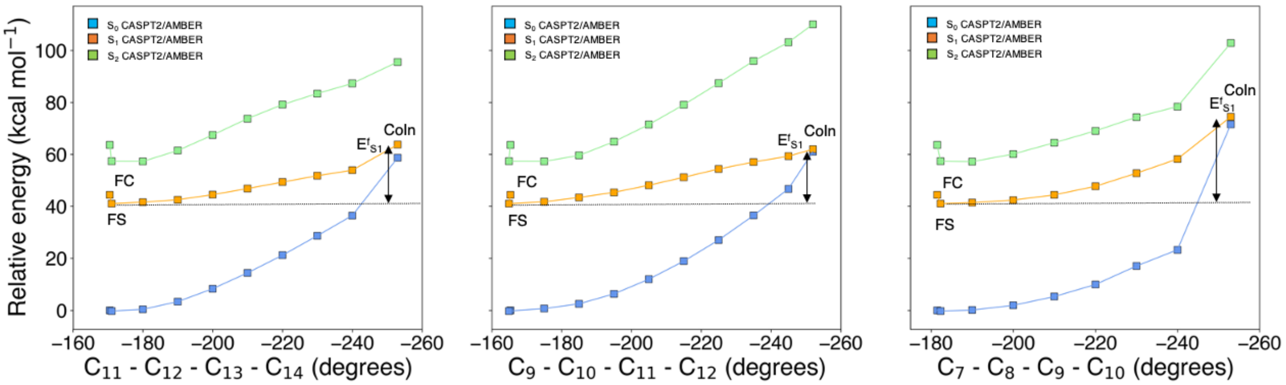

Supplementary Figure 10. Respectively from left to right: photoisomerization MEP associated with the CCW rotations C12-C13, C10-C11, C8-C9 rPSB single bonds. Energies are relative to the DA. For each MEPs, 3 root state average CASPT2/CASSCF(12,12)/6-31G*/AMBER94 (CASPT2/AMBER) energy profiles are reported. E^f_{S1} indicates the S_1 isomerization barrier and corresponds to the CoIn and FS S_1 energy difference.

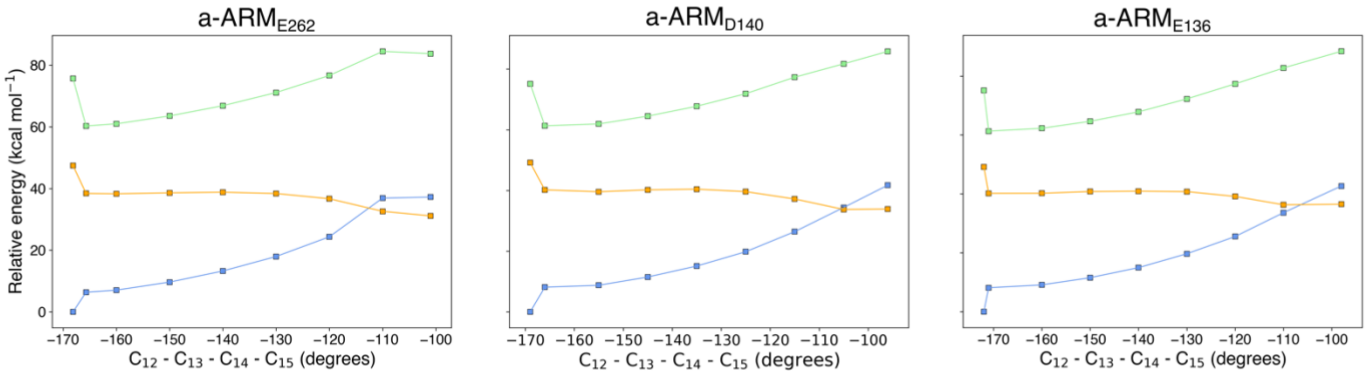

Supplementary Figure 11. Respectively from left to right: photoisomerization MEP associated with the CW rotation of the C13=C14 double bond for NeoR models featuring E262 (a-ARM$_{E262}$), D140 (a-ARM$_{D140}$) and E136 (a-ARM$_{E136}$) residues as rPSB counterions. S_0 (in blue), S_1 (in orange) and S_2 (in green) energies are relative to the DA. For each MEPs, 3 root state average CASPT2/CASSCF(12,12)/6-31G*/AMBER94 (CASPT2/AMBER) energy profiles are reported.

The plot in Fig. S13 is generated using the orthogonalized branching plane (BP) vectors that corresponds to the gradient difference, $\boldsymbol{X_1} = \frac{\partial (V_{S1} - V_{S0})}{\partial R}$, and derivative coupling, $\boldsymbol{X_2} = \langle \psi_{S0} | \frac{\partial}{\partial R} | \psi_{S1} \rangle$, where V_{S1} and V_{S0} are the Born-Oppenheimer (adiabatic) potential energy surfaces and ψ_{S0} and ψ_{S1} are the corresponding wavefunctions. $\mathbf{X_1}$ and $\mathbf{X_2}$ components include both BLA_{PSB} (defined as C14-C15 and C15=N bond lengths difference) and α geometrical coordinates. Indeed, the $\mathbf{X_1}$ and $\mathbf{X_2}$ vectors represented by arrows applied to the rPSB atoms (see Supplementary Figure 12) clearly show components of the BLA_{PSB} and C12-C13=C14-C15 torsional (i.e. α) motions. More specifically, it is found that $\mathbf{X_1}$ is dominated by α (+X_1 describes a mode pointing back to the all-trans reactant) while $\mathbf{X_2}$ is dominated by the C15=N bond elongation/contraction (+X_2 describes a strong C15-N contraction).

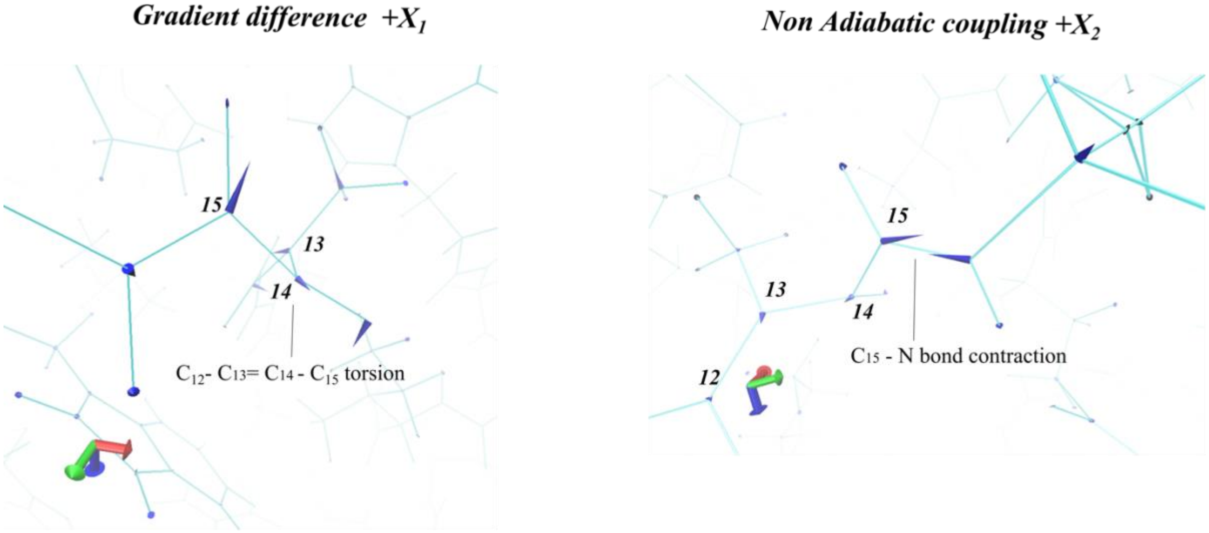

Supplementary Figure 12. Pictorial representation of the $+X_1$ (left) and $+X_2$ (right) branching vectors at the rPSB chromophore CoIn geometry along the C13=C14 photoisomerization path.

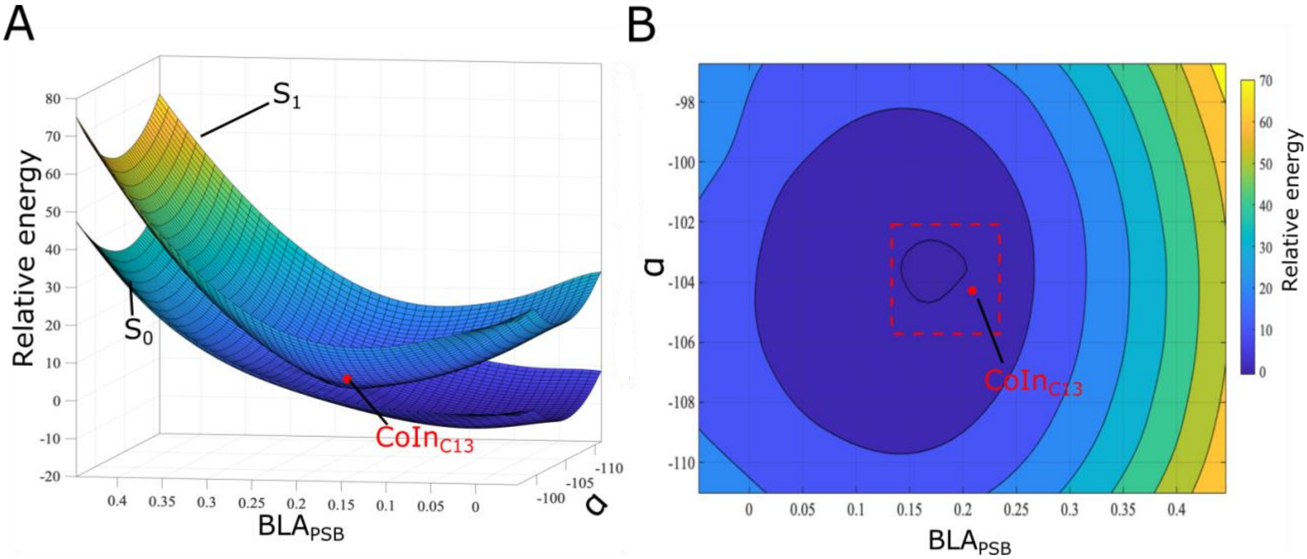

Supplementary Figure 13. A) Three-dimensional representation of the 2 state average CASSCF(12,12)/6-31G*/AMBER94 S_1/S_0 branching space around the CoIn located along the C13=C14 photoisomerization (CoIn) and B) the corresponding S_1 state contour plot. BLA_{PSB} coordinates corresponds to -C14-C15- and -C15-N- bond length difference (Å) while α to the C12-C13-C14-C15 dihedral angle (degrees). The energies (in kcal mol^{-1}) shown are relative to the

crossing point energy. The maps were calculated by sampling the S_0 and S_1 energies at the points of a square 2D grid generated by the $\mathbf{X_1}$ (non adiabatic coupling) $\mathbf{X_2}$ (gradient difference) branching vectors of the CoIn. Although the analysis can confidentially describe the close neighborhood of the branching space around CoIn, B diagram clearly shows a small S_1 region lower in energy than the CoIn. Therefore, the CoIn must have a sloped, rather than a peaked, topography.

Supplementary references:

1. Melaccio, F. *et al.* Toward Automatic Rhodopsin Modeling as a Tool for High-Throughput Computational Photobiology. *Journal of Chemical Theory and Computation* **12**, 6020–6034 (2016).

2. Pedraza-González, L., de Vico, L., Marín, M. del C., Fanelli, F. & Olivucci, M. *a* -ARM: Automatic Rhodopsin Modeling with Chromophore Cavity Generation, Ionization State Selection, and External Counterion Placement. *Journal of Chemical Theory and Computation* **15**, 3134–3152 (2019).

3. Broser, M. *et al.* NeoR, a near-infrared absorbing rhodopsin. *Nature Communications* **11**, 5682 (2020).

4. Cornell, W. D. *et al.* A Second Generation Force Field for the Simulation of Proteins, Nucleic Acids, and Organic Molecules. *J Am Chem Soc* **117**, 5179–5197 (1995).

5. Broser, M. *et al.* NeoR, a near-infrared absorbing rhodopsin. *Nature Communications* **11**, (2020).

6. Manathunga, M. *et al.* Probing the Photodynamics of Rhodopsins with Reduced Retinal Chromophores. *Journal of Chemical Theory and Computation* **12**, 839–850 (2016).

7. Schnedermann, C. *et al.* Evidence for a vibrational phase-dependent isotope effect on the photochemistry of vision. *Nature Chemistry* **10**, 1–7 (2018).

8. Lu, T. & Chen, F. Multiwfn: A multifunctional wavefunction analyzer. *Journal of Computational Chemistry* **33**, 580–592 (2012).

9. Aquilante, F. *et al.* Modern quantum chemistry with [open]molcas. *Journal of Chemical Physics* **152**, (2020).

10. Alex A. Granovsky, Firefly version 8, www http://classic.chem.msu.su/gran/firefly/index.html.

11. Nogly, P. *et al.* Retinal isomerization in bacteriorhodopsin captured by a femtosecond x-ray laser. *Science (1979)* **361**, (2018).

12. The Ultrafast Photoisomerizations of Rhodopsin and Bathorhodopsin Are Modulated by Bond Length Alternation and HOOP Driven Electronic Effects.

13. Luk, H. L., Melaccio, F., Rinaldi, S., Gozem, S. & Olivucci, M. Molecular bases for the selection of the chromophore of animal rhodopsins. *Proceedings of the National Academy of Sciences* **112**, 15297–15302 (2015).

14. Strambi, A., Durbeej, B., Ferré, N. & Olivucci, M. *Anabaena* sensory rhodopsin is a light-driven unidirectional rotor. *Proceedings of the National Academy of Sciences* **107**, 21322–21326 (2010).

15. Rinaldi, S., Melaccio, F., Gozem, S., Fanelli, F. & Olivucci, M. Comparison of the isomerization mechanisms of human melanopsin and invertebrate and vertebrate rhodopsins. *Proceedings of the National Academy of Sciences* **111**, 1714–1719 (2014).

16. Frutos, L. M., Andruniów, T., Santoro, F., Ferré, N. & Olivucci, M. Tracking the excited-state time evolution of the visual pigment with multiconfigurational quantum chemistry. *Proceedings of the National Academy of Sciences* **104**, 7764–7769 (2007).

17. Altoè, P., Cembran, A., Olivucci, M. & Garavelli, M. Aborted double bicycle-pedal isomerization with hydrogen bond breaking is the primary event of bacteriorhodopsin proton pumping. *Proceedings of the National Academy of Sciences* **107**, 20172–20177 (2010).

18. Hontani, Y. *et al.* Reaction dynamics of the chimeric channelrhodopsin. *Scientific Reports* **7**, (2017).

19. Dokukina, I. & Weingart, O. Spectral properties and isomerisation path of retinal in C1C2 channelrhodopsin. *Physical Chemistry Chemical Physics* **17**, 25142–25150 (2015).

20. Szymczak, J. J., Barbatti, M. & Lischka, H. Mechanism of Ultrafast Photodecay in Restricted Motions in Protonated Schiff Bases: The Pentadieniminium Cation. *Journal of Chemical Theory and Computation* **4**, 1189–1199 (2008).

21. Schapiro, I., Weingart, O. & Buss, V. Bicycle-Pedal Isomerization in a Rhodopsin Chromophore Model. *J Am Chem Soc* **131**, 16–17 (2009).

22. Page, C. S. & Olivucci, M. Ground and excited state CASPT2 geometry optimizations of small organic molecules. *Journal of Computational Chemistry* **24**, 298–309 (2003).

23. Gozem, S., Melaccio, F., Luk, H. L., Rinaldi, S. & Olivucci, M. Learning from photobiology how to design molecular devices using a computer. *Chem. Soc. Rev.* **43**, 4019–4036 (2014).

24. Shiozaki, T., Győrffy, W., Celani, P. & Werner, H.-J. Communication: Extended multi-state complete active space second-order perturbation theory: Energy and nuclear gradients. *The Journal of Chemical Physics* **135**, 081106 (2011).

25. Marín, M. D. C. *et al.* Fluorescence Enhancement of a Microbial Rhodopsin via Electronic Reprogramming. *J Am Chem Soc* **141**, 262–271 (2019).

26. Lin, C. Y., Romei, M. G., Oltrogge, L. M., Mathews, I. I. & Boxer, S. G. Unified Model for Photophysical and Electro-Optical Properties of Green Fluorescent Proteins. *J Am Chem Soc* **141**, 15250–15265 (2019).

27. Lin, C.-Y., Romei, M. G., Mathews, I. I. & Boxer, S. G. Energetic Basis and Design of Enzyme Function Demonstrated Using GFP, an Excited-State Enzyme. *J Am Chem Soc* **144**, 3968–3978 (2022).
