# Supplementary material for: Retinal chromophore charge delocalization and confinement explain the extreme photophysics of Neorhodopsin
Source: Nat Commun. 2022 Nov 4;13:6652. doi: 10.1038/s41467-022-33953-y (PMC9636224; doi:10.1038/s41467-022-33953-y)
Supplement: Supplementary file 3 — Description of Additional Supplementary Files [file 41467_2022_33953_MOESM3_ESM.pdf]

DESCRIPTION OF ADDITIONAL SUPPLEMENTARY FILES

File Name: Supplementary Data 1.docx
Description: This file contains the cartesian coordinates of the Dark Adapted State (DA) of the QM/MM model of NeoR.

File Name: Supplementary Data 2.docx
Description: This file contains the cartesian coordinates of the Fluorescent State (FS) of the QM/MM model of NeoR

File Name: Source Data.xlsx
Description: This file contains the Source Data of the 4 computed photoisomerization reactions of NeoR shown in Figure 4. The file is organized in four separated sheets:
1) Source Data 1. It contains data for the C13=C14 photoisomerization computed in protein, in absence of the whole protein charge, in absence of the E141 residue charge and in vacuo.
2) Source Data 2. It contains data for the C11=C12 photoisomerization computed in protein, in absence of the whole protein charge, in absence of the E141 residue charge and in vacuo.
3) Source Data 3. It contains data for the C9=C10 photoisomerization computed in protein, in absence of the whole protein charge, in absence of the E141 residue charge and in vacuo.
4) Source Data 4. It contains data for the C7=C8 photoisomerization computed in protein, in absence of the whole protein charge, in absence of the E141 residue charge and in vacuo.
