# Supplementary material for: Retinal chromophore charge delocalization and confinement explain the extreme photophysics of Neorhodopsin
Source: Nat Commun. 2022 Nov 4;13:6652. doi: 10.1038/s41467-022-33953-y (PMC9636224; doi:10.1038/s41467-022-33953-y)
Supplement: Supplementary file 5 — Supplementary Data 2 [file 41467_2022_33953_MOESM5_ESM.docx]

**Supplementary information for:**

**Retinal chromophore charge delocalization and confinement explain the extreme photophysics of Neorhodopsin**

Riccardo Palombo ^1,^*^2^*, Leonardo Barneschi^1^ Laura Pedraza-González^1^, Daniele Padula^1^, Igor Schapiro*^3^* and Massimo Olivucci*^1,2^*

*^1^ Dipartimento di Biotecnologie, Chimica e Farmacia, Università di Siena, via A. Moro 2,*

*I-53100 Siena, Siena, Italy.*

*^2^ Department of Chemistry, Bowling Green State University, Bowling Green, Ohio 43403*, *United*

*^3^ Fritz Haber Center for Molecular Dynamics, Institute of Chemistry, The Hebrew*

*University of Jerusalem, 9190401 Jerusalem, Israel.*

*Correspondence to:* [*olivucci@unisi.it,*](mailto:molivuc@bgsu.edu) *molivuc@bgsu.edu*

**This document includes the cartesian coordinates (in XYZ format) Fluorescent State (FS) of the QM/MM model of NeoR used in this work:**

3764

1 N3 -4.010000 12.090000 24.550000 356 2 3 4 5

2 H -4.910000 11.910000 24.990000 359 1

3 H -4.210000 12.440000 23.620000 359 1

4 H -3.490000 12.800000 25.050000 359 1

5 CT -3.220000 10.850000 24.460000 357 1 6 7 11

6 H1 -3.020000 10.480000 25.460000 361 5

7 CT -3.980000 9.780000 23.660000 13 5 8 9 10

8 HC -3.400000 8.860000 23.550000 14 7

9 HC -4.860000 9.490000 24.230000 14 7

10 HC -4.300000 10.160000 22.690000 14 7

11 C -1.920000 11.100000 23.790000 358 5 12 13

12 O -1.150000 11.970000 24.200000 360 11

13 N -1.640000 10.340000 22.720000 15 11 14 15

14 H -2.320000 9.710000 22.320000 18 13

15 CT -0.390000 10.530000 22.050000 16 13 16 17 27

16 H1 0.430000 10.530000 22.770000 20 15

17 CT -0.120000 9.510000 20.980000 21 15 18 19 23

18 HC 0.830000 9.740000 20.500000 22 17

19 CT 0.010000 8.130000 21.650000 23 17 20 21 22

20 HC 0.250000 7.390000 20.890000 24 19

21 HC 0.820000 8.160000 22.390000 24 19

22 HC -0.920000 7.850000 22.140000 24 19

23 CT -1.240000 9.580000 19.930000 25 17 24 25 26

24 HC -1.110000 8.730000 19.260000 26 23

25 HC -2.230000 9.500000 20.370000 26 23

26 HC -1.200000 10.450000 19.290000 26 23

27 C -0.450000 11.860000 21.390000 17 15 28 29

28 O -1.410000 12.210000 20.700000 19 27

29 N 0.600000 12.670000 21.620000 210 27 30 31

30 H 1.320000 12.430000 22.280000 213 29

31 CT 0.630000 13.960000 21.020000 211 29 32 33 39

32 H1 -0.370000 14.380000 20.940000 215 31

33 CT 1.540000 14.950000 21.770000 216 31 34 35 36

34 HC 1.570000 15.910000 21.250000 217 33

35 HC 2.550000 14.540000 21.850000 217 33

36 C 0.950000 15.160000 23.160000 218 33 37 38

37 O2 -0.180000 14.670000 23.400000 219 36

38 O2 1.620000 15.820000 24.000000 219 36

39 C 1.200000 13.780000 19.670000 212 31 40 41

40 O 1.680000 12.700000 19.330000 214 39

41 N 1.120000 14.820000 18.830000 287 39 42 43

42 H 0.850000 15.710000 19.220000 290 41

43 CT 1.750000 14.720000 17.550000 288 41 44 45 63

44 H1 1.520000 13.760000 17.080000 292 43

45 CT 1.430000 15.880000 16.580000 293 43 46 47 48

46 HC 2.150000 15.880000 15.760000 294 45

47 HC 1.530000 16.810000 17.140000 294 45

48 CT 0.010000 15.810000 15.980000 295 45 49 50 51

49 HC -0.710000 15.810000 16.800000 296 48

50 HC -0.080000 14.860000 15.450000 296 48

51 CT -0.350000 16.950000 15.000000 297 48 52 53 54

52 H1 0.400000 17.020000 14.220000 298 51

53 H1 -0.390000 17.860000 15.590000 298 51

54 N2 -1.680000 16.620000 14.400000 299 51 55 56

55 H -1.890000 15.670000 14.120000 300 54

56 CA -2.600000 17.590000 14.180000 301 54 57 60

57 N2 -2.330000 18.890000 14.520000 302 56 58 59

58 H -2.970000 19.610000 14.250000 303 57

59 H -1.390000 19.140000 14.770000 303 57

60 N2 -3.810000 17.280000 13.620000 302 56 61 62

61 H -4.360000 18.020000 13.220000 303 60

62 H -3.940000 16.380000 13.170000 303 60

63 C 3.210000 14.690000 17.880000 289 43 64 65

64 O 3.610000 15.040000 18.980000 291 63

65 N 4.060000 14.270000 16.920000 1 63 66 67

66 H 3.750000 14.030000 15.990000 4 65

67 CT 5.450000 14.060000 17.180000 2 65 68 69 70

68 H1 5.910000 13.420000 16.440000 6 67

69 H1 5.540000 13.520000 18.120000 6 67

70 C 6.160000 15.370000 17.370000 3 67 71 72

71 O 5.570000 16.360000 17.800000 5 70

72 N 7.480000 15.360000 17.080000 220 70 73 74

73 H 7.730000 14.610000 16.460000 223 72

74 CT 8.380000 16.470000 17.280000 221 72 75 76 84

75 H1 8.340000 16.750000 18.330000 225 74

76 CT 9.820000 16.110000 16.870000 226 74 77 78 79

77 HC 9.900000 16.710000 16.020000 227 76

78 HC 10.090000 15.060000 16.810000 227 76

79 C 10.820000 17.040000 17.550000 228 76 80 81

80 O 10.470000 18.080000 18.110000 229 79

81 N 12.130000 16.640000 17.510000 230 79 82 83

82 H 12.770000 17.300000 17.920000 231 81

83 H 12.430000 15.790000 17.070000 231 81

84 C 7.910000 17.620000 16.450000 222 74 85 86

85 O 7.210000 17.460000 15.460000 224 84

86 N 8.290000 18.850000 16.880000 55 84 87 88

87 H 8.860000 18.900000 17.720000 58 86

88 CT 7.890000 20.070000 16.260000 56 86 89 90 95

89 H1 6.820000 20.170000 16.360000 60 88

90 CT 8.440000 21.310000 16.990000 61 88 91 92 93

91 H1 8.140000 22.210000 16.450000 62 90

92 H1 9.530000 21.260000 17.030000 62 90

93 OH 7.920000 21.370000 18.310000 63 90 94

94 HO 8.340000 22.100000 18.770000 64 93

95 C 8.390000 20.130000 14.860000 57 88 96 97

96 O 7.670000 20.530000 13.950000 59 95

97 N 9.660000 19.740000 14.630000 210 95 98 99

98 H 10.290000 19.620000 15.410000 213 97

99 CT 10.180000 19.850000 13.300000 211 97 100 101 107

100 H1 10.090000 20.880000 12.930000 215 99

101 CT 11.640000 19.380000 13.190000 216 99 102 103 104

102 HC 11.940000 19.360000 12.140000 217 101

103 HC 11.750000 18.380000 13.610000 217 101

104 C 12.530000 20.360000 13.950000 218 101 105 106

105 O2 12.000000 21.420000 14.390000 219 104

106 O2 13.740000 20.060000 14.090000 219 104

107 C 9.370000 18.950000 12.420000 212 99 108 109

108 O 8.930000 19.340000 11.330000 214 107

109 N 9.130000 17.730000 12.910000 107 107 110 111

110 H 9.410000 17.550000 13.860000 110 109

111 CT 8.430000 16.730000 12.160000 108 109 112 113 127

112 H1 8.910000 16.580000 11.200000 112 111

113 CT 8.370000 15.400000 12.920000 113 111 114 115 116

114 HC 7.960000 14.670000 12.310000 114 113

115 HC 8.100000 15.600000 13.930000 114 113

116 CA 9.750000 14.840000 13.000000 115 113 117 125

117 CA 10.230000 14.040000 11.990000 116 116 118 119

118 HA 9.610000 13.820000 11.130000 117 117

119 CA 11.500000 13.520000 12.050000 118 117 120 121

120 HA 11.880000 12.900000 11.250000 119 119

121 CA 12.300000 13.800000 13.140000 120 119 122 123

122 HA 13.310000 13.420000 13.180000 121 121

123 CA 11.830000 14.590000 14.150000 118 121 124 125

124 HA 12.500000 14.900000 14.940000 119 123

125 CA 10.560000 15.110000 14.080000 116 116 123 126

126 HA 10.340000 16.030000 14.460000 117 125

127 C 7.020000 17.200000 11.910000 109 111 128 129

128 O 6.510000 17.080000 10.800000 111 127

129 N 6.370000 17.760000 12.950000 41 127 130 131

130 H 6.830000 17.760000 13.850000 44 129

131 CT 5.000000 18.160000 12.870000 42 129 132 133 146

132 H1 4.430000 17.300000 12.520000 46 131

133 CT 4.430000 18.570000 14.210000 47 131 134 135 139

134 HC 4.680000 17.800000 14.940000 48 133

135 CT 5.100000 19.890000 14.640000 51 133 136 137 138

136 HC 4.780000 20.140000 15.650000 52 135

137 HC 6.040000 19.470000 14.760000 52 135

138 HC 4.940000 20.780000 14.050000 52 135

139 CT 2.890000 18.650000 14.180000 49 133 140 141 142

140 HC 2.500000 17.670000 13.910000 50 139

141 HC 2.560000 19.370000 13.430000 50 139

142 CT 2.320000 19.070000 15.540000 53 139 143 144 145

143 HC 1.240000 19.060000 15.510000 54 142

144 HC 2.700000 18.420000 16.330000 54 142

145 HC 2.610000 20.100000 15.770000 54 142

146 C 4.810000 19.270000 11.880000 43 131 147 148

147 O 3.900000 19.220000 11.060000 45 146

148 N 5.680000 20.300000 11.910000 122 146 149 150

149 H 6.430000 20.320000 12.600000 125 148

150 CT 5.430000 21.400000 11.020000 123 148 151 152 167

151 H1 4.420000 21.770000 11.180000 127 150

152 CT 6.430000 22.560000 11.170000 128 150 153 154 155

153 HC 7.440000 22.250000 10.890000 129 152

154 HC 6.440000 22.910000 12.200000 129 152

155 CA 5.930000 23.630000 10.270000 130 152 156 165

156 CA 4.960000 24.500000 10.710000 131 155 157 158

157 HA 4.580000 24.410000 11.720000 132 156

158 CA 4.480000 25.500000 9.900000 133 156 159 160

159 HA 3.730000 26.180000 10.270000 134 158

160 C 4.960000 25.630000 8.620000 135 158 161 163

161 OH 4.470000 26.660000 7.780000 136 160 162

162 HO 3.810000 27.190000 8.220000 137 161

163 CA 5.930000 24.770000 8.160000 133 160 164 165

164 HA 6.310000 24.900000 7.160000 134 163

165 CA 6.410000 23.770000 8.990000 131 155 163 166

166 HA 7.180000 23.100000 8.630000 132 165

167 C 5.530000 20.900000 9.630000 124 150 168 169

168 O 4.700000 21.220000 8.780000 126 167

169 N 6.550000 20.070000 9.350000 271 167 170 171

170 H 7.210000 19.840000 10.090000 274 169

171 CT 6.730000 19.590000 8.020000 272 169 172 173 189

172 H1 6.820000 20.420000 7.320000 276 171

173 CT 7.930000 18.630000 7.870000 277 171 174 175 176

174 HC 7.950000 18.390000 6.830000 278 173

175 HC 7.840000 17.760000 8.520000 278 173

176 CT 9.290000 19.300000 8.020000 279 173 177 178 179

177 HC 9.280000 19.930000 8.910000 280 176

178 HC 9.470000 19.950000 7.160000 280 176

179 CT 10.450000 18.310000 8.150000 281 176 180 181 182

180 HC 10.260000 17.670000 9.010000 282 179

181 HC 11.360000 18.880000 8.350000 282 179

182 CT 10.670000 17.450000 6.900000 283 179 183 184 185

183 HP 10.880000 18.100000 6.040000 284 182

184 HP 9.760000 16.890000 6.680000 284 182

185 N3 11.810000 16.520000 7.110000 285 182 186 187 188

186 H 12.000000 16.050000 6.220000 286 185

187 H 12.660000 17.050000 7.270000 286 185

188 H 11.650000 15.860000 7.850000 286 185

189 C 5.510000 18.820000 7.650000 273 171 190 191

190 O 4.990000 18.970000 6.550000 275 189

191 N 5.000000 18.000000 8.580000 1 189 192 193

192 H 5.470000 17.920000 9.480000 4 191

193 CT 3.880000 17.160000 8.270000 2 191 194 195 196

194 H1 3.580000 16.750000 9.240000 6 193

195 H1 3.980000 16.250000 7.700000 6 193

196 C 2.710000 17.990000 7.860000 3 193 197 198

197 O 2.020000 17.650000 6.900000 5 196

198 N 2.470000 19.110000 8.560000 65 196 199 200

199 H 3.040000 19.340000 9.370000 68 198

200 CT 1.330000 19.920000 8.230000 66 198 201 202 210

201 H1 0.420000 19.340000 8.280000 70 200

202 CT 1.200000 21.130000 9.100000 71 200 203 204 208

203 H1 2.070000 21.780000 8.980000 72 202

204 CT -0.070000 21.900000 8.680000 75 202 205 206 207

205 HC -0.220000 22.760000 9.330000 76 204

206 HC 0.160000 22.240000 7.710000 76 204

207 HC -0.950000 21.250000 8.690000 76 204

208 OH 1.100000 20.750000 10.460000 73 202 209

209 HO 1.100000 21.570000 10.960000 74 208

210 C 1.520000 20.400000 6.830000 67 200 211 212

211 O 0.600000 20.370000 6.010000 69 210

212 N 2.750000 20.830000 6.510000 65 210 213 214

213 H 3.460000 20.880000 7.230000 68 212

214 CT 3.070000 21.360000 5.220000 66 212 215 216 224

215 H1 2.440000 22.210000 5.000000 70 214

216 CT 4.490000 21.830000 5.130000 71 214 217 218 222

217 H1 5.180000 21.010000 5.300000 72 216

218 CT 4.740000 22.380000 3.720000 75 216 219 220 221

219 HC 5.740000 22.800000 3.680000 76 218

220 HC 4.690000 21.600000 2.960000 76 218

221 HC 4.020000 23.170000 3.490000 76 218

222 OH 4.740000 22.840000 6.100000 73 216 223

223 HO 5.670000 23.060000 6.080000 74 222

224 C 2.890000 20.280000 4.200000 67 214 225 226

225 O 2.350000 20.520000 3.120000 69 224

226 N 3.310000 19.050000 4.530000 107 224 227 228

227 H 3.720000 18.910000 5.440000 110 226

228 CT 3.260000 17.960000 3.610000 108 226 229 230 244

229 H1 3.820000 18.220000 2.710000 112 228

230 CT 3.750000 16.610000 4.160000 113 228 231 232 233

231 HC 3.490000 15.820000 3.450000 114 230

232 HC 3.260000 16.380000 5.100000 114 230

233 CA 5.240000 16.610000 4.330000 115 230 234 242

234 CA 6.060000 16.630000 3.230000 116 233 235 236

235 HA 5.640000 16.660000 2.240000 117 234

236 CA 7.430000 16.620000 3.380000 118 234 237 238

237 HA 8.070000 16.650000 2.510000 119 236

238 CA 7.990000 16.580000 4.630000 120 236 239 240

239 HA 9.070000 16.570000 4.710000 121 238

240 CA 7.170000 16.540000 5.740000 118 238 241 242

241 HA 7.580000 16.340000 6.720000 119 240

242 CA 5.800000 16.550000 5.580000 116 233 240 243

243 HA 5.220000 16.350000 6.410000 117 242

244 C 1.840000 17.740000 3.220000 109 228 245 246

245 O 1.540000 17.450000 2.060000 111 244

246 N 0.920000 17.890000 4.190000 15 244 247 248

247 H 1.240000 18.130000 5.120000 18 246

248 CT -0.470000 17.600000 3.980000 16 246 249 250 260

249 H1 -0.580000 16.560000 3.690000 20 248

250 CT -1.310000 17.880000 5.190000 21 248 251 252 256

251 HC -1.240000 18.920000 5.480000 22 250

252 CT -2.780000 17.640000 4.830000 23 250 253 254 255

253 HC -3.450000 17.580000 5.670000 24 252

254 HC -3.180000 18.430000 4.190000 24 252

255 HC -2.850000 16.680000 4.340000 24 252

256 CT -0.790000 17.010000 6.350000 25 250 257 258 259

257 HC -1.630000 16.600000 6.910000 26 256

258 HC -0.170000 16.180000 6.010000 26 256

259 HC -0.240000 17.640000 7.010000 26 256

260 C -1.010000 18.440000 2.850000 17 248 261 262

261 O -1.900000 18.000000 2.130000 19 260

262 N -0.490000 19.660000 2.680000 107 260 263 264

263 H 0.260000 19.940000 3.310000 110 262

264 CT -0.960000 20.640000 1.730000 108 262 265 266 280

265 H1 -2.040000 20.740000 1.870000 112 264

266 CT -0.340000 22.030000 1.950000 113 264 267 268 269

267 HC -0.540000 22.670000 1.090000 114 266

268 HC 0.740000 21.980000 2.070000 114 266

269 CA -1.000000 22.630000 3.140000 115 266 270 278

270 CA -2.190000 23.310000 3.000000 116 269 271 272

271 HA -2.640000 23.410000 2.020000 117 270

272 CA -2.810000 23.880000 4.090000 118 270 273 274

273 HA -3.730000 24.420000 3.960000 119 272

274 CA -2.250000 23.770000 5.330000 120 272 275 276

275 HA -2.710000 24.270000 6.170000 121 274

276 CA -1.060000 23.100000 5.490000 118 274 277 278

277 HA -0.550000 23.310000 6.390000 119 276

278 CA -0.440000 22.530000 4.400000 116 269 276 279

279 HA 0.530000 22.090000 4.470000 117 278

280 C -0.790000 20.290000 0.260000 109 264 281 282

281 O -1.570000 20.760000 -0.560000 111 280

282 N 0.220000 19.480000 -0.120000 41 280 283 284

283 H 0.760000 19.100000 0.650000 44 282

284 CT 0.740000 19.340000 -1.480000 42 282 285 286 299

285 H1 1.110000 20.330000 -1.750000 46 284

286 CT 1.920000 18.410000 -1.500000 47 284 287 288 292

287 HC 1.760000 17.410000 -1.160000 48 286

288 CT 2.330000 18.200000 -2.980000 51 286 289 290 291

289 HC 3.330000 17.800000 -3.030000 52 288

290 HC 1.690000 17.500000 -3.510000 52 288

291 HC 2.390000 19.160000 -3.510000 52 288

292 CT 3.060000 18.960000 -0.630000 49 286 293 294 295

293 HC 2.730000 19.070000 0.400000 50 292

294 HC 3.360000 19.940000 -1.000000 50 292

295 CT 4.280000 18.040000 -0.630000 53 292 296 297 298

296 HC 4.990000 18.420000 0.100000 54 295

297 HC 3.990000 17.030000 -0.340000 54 295

298 HC 4.790000 18.030000 -1.590000 54 295

299 C -0.130000 18.910000 -2.680000 43 284 300 301

300 O -0.070000 19.610000 -3.690000 45 299

301 N -0.960000 17.830000 -2.660000 15 299 302 303

302 H -1.020000 17.350000 -1.770000 18 301

303 CT -1.610000 17.200000 -3.840000 16 301 304 305 315

304 H1 -0.830000 16.900000 -4.540000 20 303

305 CT -2.410000 15.980000 -3.490000 21 303 306 307 311

306 HC -2.910000 15.590000 -4.380000 22 305

307 CT -1.450000 14.890000 -3.000000 23 305 308 309 310

308 HC -1.920000 14.070000 -2.500000 24 307

309 HC -0.860000 14.520000 -3.840000 24 307

310 HC -0.780000 15.440000 -2.440000 24 307

311 CT -3.480000 16.370000 -2.460000 25 305 312 313 314

312 HC -3.940000 15.450000 -2.130000 26 311

313 HC -3.060000 16.860000 -1.590000 26 311

314 HC -4.240000 17.000000 -2.910000 26 311

315 C -2.540000 18.100000 -4.620000 17 303 316 317

316 O -2.710000 17.900000 -5.830000 19 315

317 N -3.190000 19.100000 -4.000000 7 315 318 319

318 H -2.980000 19.290000 -3.030000 10 317

319 CT -4.210000 19.870000 -4.680000 8 317 320 321 325

320 H1 -5.020000 19.200000 -4.970000 12 319

321 CT -4.800000 20.980000 -3.780000 13 319 322 323 324

322 HC -5.580000 21.520000 -4.320000 14 321

323 HC -5.240000 20.530000 -2.890000 14 321

324 HC -4.020000 21.680000 -3.480000 14 321

325 C -3.710000 20.530000 -5.930000 9 319 326 327

326 O -4.430000 20.580000 -6.930000 11 325

327 N -2.470000 21.050000 -5.930000 15 325 328 329

328 H -1.900000 20.960000 -5.100000 18 327

329 CT -2.000000 21.780000 -7.070000 16 327 330 331 341

330 H1 -2.650000 22.640000 -7.250000 20 329

331 CT -0.580000 22.250000 -6.910000 21 329 332 333 337

332 HC 0.070000 21.400000 -6.730000 22 331

333 CT -0.140000 22.930000 -8.220000 23 331 334 335 336

334 HC 0.840000 23.380000 -8.070000 24 333

335 HC -0.030000 22.220000 -9.040000 24 333

336 HC -0.840000 23.720000 -8.500000 24 333

337 CT -0.520000 23.160000 -5.680000 25 331 338 339 340

338 HC 0.490000 23.540000 -5.570000 26 337

339 HC -1.200000 24.000000 -5.800000 26 337

340 HC -0.760000 22.610000 -4.770000 26 337

341 C -2.040000 20.900000 -8.290000 17 329 342 343

342 O -2.430000 21.350000 -9.370000 19 341

343 N -1.650000 19.620000 -8.140000 77 341 344 345

344 H -1.370000 19.300000 -7.230000 80 343

345 CT -1.580000 18.720000 -9.260000 78 343 346 347 352

346 H1 -0.920000 19.140000 -10.020000 82 345

347 CT -1.050000 17.350000 -8.840000 83 345 348 349 350

348 H1 -1.000000 16.700000 -9.710000 84 347

349 H1 -1.720000 16.900000 -8.100000 84 347

350 SH 0.610000 17.530000 -8.130000 85 347 351

351 HS 1.030000 16.280000 -8.320000 86 350

352 C -2.940000 18.560000 -9.850000 79 345 353 354

353 O -3.090000 18.490000 -11.070000 81 352

354 N -3.970000 18.510000 -9.000000 107 352 355 356

355 H -3.790000 18.680000 -8.020000 110 354

356 CT -5.320000 18.310000 -9.450000 108 354 357 358 372

357 H1 -5.430000 17.460000 -10.090000 112 356

358 CT -6.310000 18.340000 -8.280000 113 356 359 360 361

359 HC -6.280000 19.290000 -7.760000 114 358

360 HC -6.050000 17.560000 -7.560000 114 358

361 CA -7.700000 18.120000 -8.780000 115 358 362 370

362 CA -8.220000 16.860000 -8.880000 116 361 363 364

363 HA -7.600000 16.060000 -8.580000 117 362

364 CA -9.500000 16.660000 -9.340000 118 362 365 366

365 HA -9.920000 15.660000 -9.370000 119 364

366 CA -10.270000 17.740000 -9.710000 120 364 367 368

367 HA -11.280000 17.590000 -10.080000 121 366

368 CA -9.760000 19.010000 -9.610000 118 366 369 370

369 HA -10.360000 19.860000 -9.910000 119 368

370 CA -8.480000 19.200000 -9.150000 116 361 368 371

371 HA -8.090000 20.210000 -9.080000 117 370

372 C -5.690000 19.410000 -10.390000 109 356 373 374

373 O -6.180000 19.170000 -11.490000 111 372

374 N -5.440000 20.660000 -9.980000 65 372 375 376

375 H -4.960000 20.810000 -9.100000 68 374

376 CT -5.800000 21.790000 -10.790000 66 374 377 378 386

377 H1 -6.860000 21.740000 -11.050000 70 376

378 CT -5.510000 23.100000 -10.120000 71 376 379 380 384

379 H1 -5.850000 23.920000 -10.760000 72 378

380 CT -6.280000 23.150000 -8.790000 75 378 381 382 383

381 HC -6.120000 24.120000 -8.320000 76 380

382 HC -7.350000 23.030000 -8.970000 76 380

383 HC -5.940000 22.380000 -8.100000 76 380

384 OH -4.120000 23.240000 -9.880000 73 378 385

385 HO -3.970000 24.090000 -9.470000 74 384

386 C -5.010000 21.750000 -12.060000 67 376 387 388

387 O -5.530000 22.030000 -13.140000 69 386

388 N -3.730000 21.380000 -11.950000 122 386 389 390

389 H -3.360000 21.300000 -11.010000 125 388

390 CT -2.820000 21.360000 -13.070000 123 388 391 392 407

391 H1 -2.770000 22.350000 -13.520000 127 390

392 CT -1.410000 20.940000 -12.600000 128 390 393 394 395

393 HC -1.460000 20.010000 -12.040000 129 392

394 HC -1.020000 21.710000 -11.930000 129 392

395 CA -0.470000 20.760000 -13.740000 130 392 396 405

396 CA 0.130000 21.840000 -14.340000 131 395 397 398

397 HA -0.090000 22.840000 -13.980000 132 396

398 CA 1.000000 21.670000 -15.390000 133 396 399 400

399 HA 1.460000 22.530000 -15.850000 134 398

400 C 1.280000 20.410000 -15.860000 135 398 401 403

401 OH 2.170000 20.230000 -16.940000 136 400 402

402 HO 2.450000 21.060000 -17.320000 137 401

403 CA 0.680000 19.320000 -15.270000 133 400 404 405

404 HA 0.910000 18.330000 -15.640000 134 403

405 CA -0.190000 19.500000 -14.220000 131 395 403 406

406 HA -0.660000 18.640000 -13.770000 132 405

407 C -3.330000 20.410000 -14.100000 124 390 408 409

408 O -3.400000 20.750000 -15.280000 126 407

409 N -3.720000 19.190000 -13.690000 27 407 410 411

410 H -3.660000 18.960000 -12.700000 30 409

411 CT -4.170000 18.240000 -14.660000 28 409 412 413 426

412 H1 -3.420000 18.230000 -15.450000 32 411

413 CT -4.380000 16.820000 -14.110000 33 411 414 415 416

414 HC -5.150000 16.830000 -13.340000 34 413

415 HC -3.430000 16.590000 -13.670000 34 413

416 CT -4.730000 15.830000 -15.230000 35 413 417 418 422

417 HC -5.590000 16.330000 -15.570000 36 416

418 CT -3.590000 15.740000 -16.260000 37 416 419 420 421

419 HC -3.780000 14.960000 -16.990000 38 418

420 HC -3.370000 16.690000 -16.720000 38 418

421 HC -2.690000 15.430000 -15.730000 38 418

422 CT -5.130000 14.460000 -14.680000 39 416 423 424 425

423 HC -5.420000 13.850000 -15.520000 40 422

424 HC -4.510000 14.090000 -13.970000 40 422

425 HC -6.010000 14.770000 -14.180000 40 422

426 C -5.460000 18.700000 -15.260000 29 411 427 428

427 O -5.710000 18.480000 -16.440000 31 426

428 N -6.320000 19.350000 -14.460000 1 426 429 430

429 H -6.080000 19.480000 -13.480000 4 428

430 CT -7.610000 19.770000 -14.940000 2 428 431 432 433

431 H1 -8.160000 20.270000 -14.140000 6 430

432 H1 -8.180000 18.910000 -15.280000 6 430

433 C -7.430000 20.720000 -16.080000 3 430 434 435

434 O -8.150000 20.650000 -17.070000 5 433

435 N -6.470000 21.650000 -15.960000 122 433 436 437

436 H -5.930000 21.690000 -15.100000 125 435

437 CT -6.260000 22.620000 -17.010000 123 435 438 439 454

438 H1 -7.190000 23.140000 -17.230000 127 437

439 CT -5.160000 23.640000 -16.680000 128 437 440 441 442

440 HC -4.230000 23.140000 -16.430000 129 439

441 HC -5.470000 24.260000 -15.840000 129 439

442 CA -4.990000 24.480000 -17.910000 130 439 443 452

443 CA -5.800000 25.570000 -18.130000 131 442 444 445

444 HA -6.560000 25.820000 -17.410000 132 443

445 CA -5.650000 26.340000 -19.260000 133 443 446 447

446 HA -6.290000 27.190000 -19.420000 134 445

447 C -4.680000 26.020000 -20.180000 135 445 448 450

448 OH -4.510000 26.800000 -21.340000 136 447 449

449 HO -5.040000 27.600000 -21.290000 137 448

450 CA -3.860000 24.930000 -19.970000 133 447 451 452

451 HA -3.080000 24.690000 -20.670000 134 450

452 CA -4.020000 24.170000 -18.830000 131 442 450 453

453 HA -3.360000 23.330000 -18.660000 132 452

454 C -5.820000 21.900000 -18.240000 124 437 455 456

455 O -6.270000 22.200000 -19.340000 126 454

456 N -4.940000 20.900000 -18.070000 41 454 457 458

457 H -4.630000 20.720000 -17.120000 44 456

458 CT -4.360000 20.160000 -19.150000 42 456 459 460 473

459 H1 -3.850000 20.840000 -19.830000 46 458

460 CT -3.410000 19.090000 -18.690000 47 458 461 462 466

461 HC -3.910000 18.480000 -17.970000 48 460

462 CT -2.990000 18.290000 -19.930000 51 460 463 464 465

463 HC -2.300000 17.560000 -19.730000 52 462

464 HC -3.740000 17.860000 -20.530000 52 462

465 HC -2.470000 18.980000 -20.600000 52 462

466 CT -2.230000 19.680000 -17.920000 49 460 467 468 469

467 HC -2.480000 20.380000 -17.170000 50 466

468 HC -1.680000 20.290000 -18.640000 50 466

469 CT -1.250000 18.600000 -17.450000 53 466 470 471 472

470 HC -0.410000 19.200000 -17.220000 54 469

471 HC -1.590000 17.970000 -16.630000 54 469

472 HC -0.820000 18.100000 -18.290000 54 469

473 C -5.420000 19.440000 -19.910000 43 458 474 475

474 O -5.340000 19.330000 -21.140000 45 473

475 N -6.450000 18.930000 -19.210000 1 473 476 477

476 H -6.440000 19.040000 -18.200000 4 475

477 CT -7.440000 18.080000 -19.820000 2 475 478 479 480

478 H1 -8.210000 17.800000 -19.100000 6 477

479 H1 -7.090000 17.240000 -20.320000 6 477

480 C -8.070000 18.760000 -20.990000 3 477 481 482

481 O -8.380000 18.110000 -21.990000 5 480

482 N -8.280000 20.080000 -20.920000 65 480 483 484

483 H -7.940000 20.610000 -20.130000 68 482

484 CT -8.920000 20.740000 -22.010000 66 482 485 486 494

485 H1 -9.900000 20.300000 -22.190000 70 484

486 CT -9.080000 22.220000 -21.790000 71 484 487 488 492

487 H1 -9.620000 22.660000 -22.630000 72 486

488 CT -9.900000 22.440000 -20.500000 75 486 489 490 491

489 HC -10.070000 23.510000 -20.360000 76 488

490 HC -10.870000 21.940000 -20.590000 76 488

491 HC -9.370000 22.060000 -19.630000 76 488

492 OH -7.810000 22.840000 -21.670000 73 486 493

493 HO -7.920000 23.660000 -21.190000 74 492

494 C -8.100000 20.560000 -23.250000 67 484 495 496

495 O -8.630000 20.350000 -24.340000 69 494

496 N -6.760000 20.650000 -23.110000 271 494 497 498

497 H -6.420000 20.780000 -22.160000 274 496

498 CT -5.830000 20.600000 -24.200000 272 496 499 500 516

499 H1 -6.140000 21.330000 -24.950000 276 498

500 CT -4.390000 20.880000 -23.750000 277 498 501 502 503

501 HC -3.730000 20.750000 -24.610000 278 500

502 HC -4.070000 20.160000 -23.000000 278 500

503 CT -4.180000 22.290000 -23.220000 279 500 504 505 506

504 HC -3.140000 22.370000 -22.890000 280 503

505 HC -4.820000 22.450000 -22.350000 280 503

506 CT -4.460000 23.390000 -24.250000 281 503 507 508 509

507 HC -5.510000 23.330000 -24.550000 282 506

508 HC -3.840000 23.230000 -25.140000 282 506

509 CT -4.240000 24.800000 -23.720000 283 506 510 511 512

510 HP -4.780000 24.910000 -22.780000 284 509

511 HP -4.650000 25.520000 -24.440000 284 509

512 N3 -2.800000 25.060000 -23.520000 285 509 513 514 515

513 H -2.610000 25.970000 -23.120000 286 512

514 H -2.340000 24.410000 -22.900000 286 512

515 H -2.240000 25.010000 -24.360000 286 512

516 C -5.840000 19.280000 -24.930000 273 498 517 518

517 O -5.730000 19.250000 -26.160000 275 516

518 N -5.950000 18.140000 -24.220000 55 516 519 520

519 H -6.250000 18.220000 -23.260000 58 518

520 CT -5.810000 16.860000 -24.850000 56 518 521 522 527

521 H1 -4.930000 16.930000 -25.460000 60 520

522 CT -5.700000 15.700000 -23.850000 61 520 523 524 525

523 H1 -4.910000 15.900000 -23.120000 62 522

524 H1 -5.550000 14.820000 -24.330000 62 522

525 OH -6.960000 15.520000 -23.210000 63 522 526

526 HO -6.900000 14.980000 -22.420000 64 525

527 C -6.980000 16.540000 -25.730000 57 520 528 529

528 O -8.050000 17.140000 -25.620000 59 527

529 N -6.780000 15.570000 -26.640000 7 527 530 531

530 H -5.850000 15.250000 -26.800000 10 529

531 CT -7.770000 15.100000 -27.570000 8 529 532 533 537

532 H1 -8.410000 15.930000 -27.860000 12 531

533 CT -7.180000 14.460000 -28.840000 13 531 534 535 536

534 HC -7.970000 14.190000 -29.540000 14 533

535 HC -6.520000 15.180000 -29.340000 14 533

536 HC -6.610000 13.560000 -28.590000 14 533

537 C -8.610000 14.070000 -26.880000 9 531 538 539

538 O -8.370000 13.710000 -25.730000 11 537

539 N -9.650000 13.570000 -27.590000 7 537 540 541

540 H -9.780000 13.880000 -28.540000 10 539

541 CT -10.520000 12.570000 -27.050000 8 539 542 543 547

542 H1 -10.910000 12.920000 -26.090000 12 541

543 CT -11.690000 12.210000 -27.980000 13 541 544 545 546

544 HC -12.330000 11.450000 -27.520000 14 543

545 HC -12.290000 13.100000 -28.170000 14 543

546 HC -11.310000 11.830000 -28.930000 14 543

547 C -9.710000 11.330000 -26.840000 9 541 548 549

548 O -8.620000 11.180000 -27.390000 11 547

549 N -10.200000 10.430000 -25.970000 271 547 550 551

550 H -11.110000 10.640000 -25.590000 274 549

551 CT -9.550000 9.210000 -25.580000 272 549 552 553 569

552 H1 -10.250000 8.540000 -25.100000 276 551

553 CT -8.820000 8.530000 -26.750000 277 551 554 555 556

554 HC -8.150000 7.850000 -26.260000 278 553

555 HC -8.050000 9.090000 -27.260000 278 553

556 CT -9.750000 7.910000 -27.800000 279 553 557 558 559

557 HC -9.170000 7.630000 -28.680000 280 556

558 HC -10.480000 8.660000 -28.110000 280 556

559 CT -10.490000 6.680000 -27.280000 281 556 560 561 562

560 HC -11.350000 6.510000 -27.930000 282 559

561 HC -10.870000 6.840000 -26.280000 282 559

562 CT -9.640000 5.410000 -27.320000 283 559 563 564 565

563 HP -9.470000 5.120000 -28.360000 284 562

564 HP -10.170000 4.590000 -26.830000 284 562

565 N3 -8.330000 5.660000 -26.660000 285 562 566 567 568

566 H -7.760000 4.820000 -26.690000 286 565

567 H -7.780000 6.350000 -27.160000 286 565

568 H -8.410000 5.920000 -25.690000 286 565

569 C -8.500000 9.580000 -24.580000 273 551 570 571

570 O -8.180000 8.800000 -23.690000 275 569

571 N -7.970000 10.810000 -24.700000 271 569 572 573

572 H -8.120000 11.300000 -25.570000 274 571

573 CT -7.050000 11.380000 -23.760000 272 571 574 575 591

574 H1 -6.320000 10.630000 -23.440000 276 573

575 CT -6.310000 12.610000 -24.300000 277 573 576 577 578

576 HC -5.630000 12.850000 -23.480000 278 575

577 HC -6.910000 13.460000 -24.280000 278 575

578 CT -5.350000 12.310000 -25.450000 279 575 579 580 581

579 HC -5.890000 11.700000 -26.180000 280 578

580 HC -4.510000 11.710000 -25.090000 280 578

581 CT -4.840000 13.550000 -26.180000 281 578 582 583 584

582 HC -5.750000 13.890000 -26.050000 282 581

583 HC -4.680000 13.260000 -27.200000 282 581

584 CT -3.690000 14.250000 -25.450000 283 581 585 586 587

585 HP -2.950000 13.540000 -25.270000 284 584

586 HP -3.890000 14.510000 -24.420000 284 584

587 N3 -3.250000 15.440000 -26.220000 285 584 588 589 590

588 H -2.500000 15.910000 -25.720000 286 587

589 H -2.840000 15.140000 -27.100000 286 587

590 H -3.980000 16.100000 -26.420000 286 587

591 C -7.850000 11.800000 -22.580000 273 573 592 593

592 O -7.380000 11.780000 -21.450000 275 591

593 N -9.110000 12.210000 -22.810000 55 591 594 595

594 H -9.390000 12.340000 -23.770000 58 593

595 CT -9.900000 12.740000 -21.740000 56 593 596 597 602

596 H1 -9.450000 13.650000 -21.360000 60 595

597 CT -11.330000 13.100000 -22.180000 61 595 598 599 600

598 H1 -11.290000 13.800000 -23.020000 62 597

599 H1 -11.850000 13.580000 -21.350000 62 597

600 OH -12.040000 11.930000 -22.560000 63 597 601

601 HO -12.970000 12.170000 -22.670000 64 600

602 C -10.010000 11.720000 -20.660000 57 595 603 604

603 O -9.940000 12.060000 -19.480000 59 602

604 N -10.140000 10.430000 -21.030000 258 602 605 606

605 H -10.250000 10.230000 -22.010000 261 604

606 CT -10.280000 9.390000 -20.050000 259 604 607 608 619

607 H1 -11.120000 9.610000 -19.390000 263 606

608 CT -10.470000 7.990000 -20.660000 264 606 609 610 611

609 HC -10.420000 7.250000 -19.860000 265 608

610 HC -9.640000 7.800000 -21.340000 265 608

611 CT -11.800000 7.810000 -21.380000 266 608 612 613 614

612 H1 -11.890000 8.560000 -22.170000 267 611

613 H1 -12.600000 7.980000 -20.660000 267 611

614 S -12.040000 6.150000 -22.100000 268 611 615

615 CT -10.840000 6.400000 -23.440000 269 614 616 617 618

616 H1 -10.810000 5.500000 -24.050000 270 615

617 H1 -11.200000 7.220000 -24.040000 270 615

618 H1 -9.840000 6.600000 -23.050000 270 615

619 C -9.040000 9.350000 -19.220000 260 606 620 621

620 O -9.100000 9.160000 -18.010000 262 619

621 N -7.870000 9.560000 -19.850000 65 619 622 623

622 H -7.850000 9.830000 -20.820000 68 621

623 CT -6.620000 9.440000 -19.150000 66 621 624 625 633

624 H1 -6.700000 8.410000 -18.820000 70 623

625 CT -5.390000 9.660000 -19.980000 71 623 626 627 631

626 H1 -4.550000 9.640000 -19.370000 72 625

627 CT -5.500000 8.810000 -21.260000 75 625 628 629 630

628 HC -4.680000 9.000000 -21.940000 76 627

629 HC -5.500000 7.750000 -21.000000 76 627

630 HC -6.410000 9.040000 -21.810000 76 627

631 OH -5.210000 11.030000 -20.310000 73 625 632

632 HO -4.640000 11.020000 -21.080000 74 631

633 C -6.630000 10.450000 -18.060000 67 623 634 635

634 O -5.880000 10.330000 -17.090000 69 633

635 N -7.440000 11.520000 -18.200000 107 633 636 637

636 H -8.040000 11.620000 -19.010000 110 635

637 CT -7.470000 12.480000 -17.140000 108 635 638 639 653

638 H1 -6.440000 12.710000 -16.870000 112 637

639 CT -8.160000 13.800000 -17.520000 113 637 640 641 642

640 HC -8.360000 14.420000 -16.650000 114 639

641 HC -9.110000 13.650000 -18.020000 114 639

642 CA -7.200000 14.550000 -18.380000 115 639 643 651

643 CA -6.260000 15.380000 -17.810000 116 642 644 645

644 HA -6.530000 15.450000 -16.810000 117 643

645 CA -5.370000 16.070000 -18.580000 118 643 646 647

646 HA -4.690000 16.760000 -18.180000 119 645

647 CA -5.410000 15.940000 -19.950000 120 645 648 649

648 HA -4.620000 16.210000 -20.610000 121 647

649 CA -6.340000 15.120000 -20.530000 118 647 650 651

650 HA -6.030000 14.630000 -21.420000 119 649

651 CA -7.230000 14.420000 -19.750000 116 642 649 652

652 HA -7.870000 13.680000 -20.160000 117 651

653 C -8.150000 11.910000 -15.890000 109 637 654 655

654 O -8.760000 12.670000 -15.150000 111 653

655 N -8.030000 10.570000 -15.630000 15 653 656 657

656 H -7.640000 10.030000 -16.390000 18 655

657 CT -8.400000 9.800000 -14.460000 16 655 658 659 669

658 H1 -9.410000 10.040000 -14.140000 20 657

659 CT -8.220000 8.310000 -14.620000 21 657 660 661 665

660 HC -7.210000 8.090000 -14.980000 22 659

661 CT -8.390000 7.660000 -13.240000 23 659 662 663 664

662 HC -8.490000 6.580000 -13.370000 24 661

663 HC -7.500000 7.790000 -12.630000 24 661

664 HC -9.280000 8.030000 -12.740000 24 661

665 CT -9.240000 7.790000 -15.640000 25 659 666 667 668

666 HC -9.670000 6.850000 -15.310000 26 665

667 HC -10.050000 8.490000 -15.820000 26 665

668 HC -8.680000 7.510000 -16.530000 26 665

669 C -7.440000 10.230000 -13.430000 17 657 670 671

670 O -7.650000 10.100000 -12.220000 19 669

671 N -6.310000 10.730000 -13.940000 27 669 672 673

672 H -6.180000 10.680000 -14.940000 30 671

673 CT -5.230000 11.180000 -13.130000 28 671 674 675 688

674 H1 -4.860000 10.380000 -12.490000 32 673

675 CT -4.090000 11.790000 -13.970000 33 673 676 677 678

676 HC -3.430000 12.100000 -13.190000 34 675

677 HC -4.460000 12.340000 -14.770000 34 675

678 CT -3.440000 10.780000 -14.930000 35 675 679 680 684

679 HC -4.170000 10.450000 -15.630000 36 678

680 CT -2.300000 11.430000 -15.740000 37 678 681 682 683

681 HC -2.230000 10.760000 -16.590000 38 680

682 HC -2.590000 12.390000 -16.180000 38 680

683 HC -1.340000 11.410000 -15.330000 38 680

684 CT -2.980000 9.510000 -14.190000 39 678 685 686 687

685 HC -2.480000 8.850000 -14.900000 40 684

686 HC -2.290000 9.770000 -13.390000 40 684

687 HC -3.830000 8.960000 -13.790000 40 684

688 C -5.770000 12.270000 -12.250000 29 673 689 690

689 O -5.370000 12.400000 -11.100000 31 688

690 N -6.700000 13.070000 -12.800000 258 688 691 692

691 H -6.990000 12.910000 -13.750000 261 690

692 CT -7.270000 14.160000 -12.050000 259 690 693 694 705

693 H1 -6.480000 14.820000 -11.700000 263 692

694 CT -8.260000 14.980000 -12.890000 264 692 695 696 697

695 HC -9.090000 14.320000 -13.150000 265 694

696 HC -7.850000 15.330000 -13.830000 265 694

697 CT -8.800000 16.220000 -12.170000 266 694 698 699 700

698 H1 -7.990000 16.840000 -11.790000 267 697

699 H1 -9.380000 15.780000 -11.390000 267 697

700 S -9.970000 17.210000 -13.160000 268 697 701

701 CT -11.270000 15.950000 -13.170000 269 700 702 703 704

702 H1 -12.150000 16.360000 -13.670000 270 701

703 H1 -10.950000 15.070000 -13.730000 270 701

704 H1 -11.540000 15.680000 -12.150000 270 701

705 C -8.010000 13.590000 -10.880000 260 692 706 707

706 O -7.930000 14.130000 -9.780000 262 705

707 N -8.740000 12.480000 -11.090000 258 705 708 709

708 H -8.680000 12.030000 -11.990000 261 707

709 CT -9.510000 11.880000 -10.040000 259 707 710 711 722

710 H1 -10.190000 12.620000 -9.610000 263 709

711 CT -10.310000 10.660000 -10.510000 264 709 712 713 714

712 HC -10.800000 10.220000 -9.640000 265 711

713 HC -9.660000 9.900000 -10.930000 265 711

714 CT -11.410000 11.000000 -11.530000 266 711 715 716 717

715 H1 -10.950000 11.470000 -12.400000 267 714

716 H1 -12.090000 11.720000 -11.080000 267 714

717 S -12.370000 9.560000 -12.100000 268 714 718

718 CT -13.440000 10.510000 -13.210000 269 717 719 720 721

719 H1 -14.140000 9.840000 -13.700000 270 718

720 H1 -14.000000 11.260000 -12.640000 270 718

721 H1 -12.830000 11.010000 -13.970000 270 718

722 C -8.590000 11.400000 -8.980000 260 709 723 724

723 O -8.870000 11.540000 -7.790000 262 722

724 N -7.440000 10.830000 -9.390000 65 722 725 726

725 H -7.280000 10.650000 -10.380000 68 724

726 CT -6.570000 10.260000 -8.410000 66 724 727 728 736

727 H1 -7.100000 9.480000 -7.860000 70 726

728 CT -5.320000 9.690000 -9.020000 71 726 729 730 734

729 H1 -4.750000 10.470000 -9.530000 72 728

730 CT -4.460000 9.100000 -7.890000 75 728 731 732 733

731 HC -3.580000 8.640000 -8.340000 76 730

732 HC -4.140000 9.870000 -7.210000 76 730

733 HC -5.040000 8.340000 -7.380000 76 730

734 OH -5.650000 8.680000 -9.960000 73 728 735

735 HO -4.900000 8.420000 -10.530000 74 734

736 C -6.150000 11.330000 -7.460000 67 726 737 738

737 O -6.310000 11.190000 -6.250000 69 736

738 N -5.620000 12.450000 -7.980000 77 736 739 740

739 H -5.590000 12.560000 -8.990000 80 738

740 CT -5.130000 13.510000 -7.140000 78 738 741 742 747

741 H1 -4.450000 13.100000 -6.390000 82 740

742 CT -4.370000 14.590000 -7.930000 83 740 743 744 745

743 H1 -3.450000 14.150000 -8.320000 84 742

744 H1 -4.110000 15.410000 -7.260000 84 742

745 SH -5.320000 15.240000 -9.330000 85 742 746

746 HS -4.360000 16.020000 -9.850000 86 745

747 C -6.260000 14.160000 -6.400000 79 740 748 749

748 O -6.090000 14.580000 -5.250000 81 747

749 N -7.440000 14.290000 -7.030000 1 747 750 751

750 H -7.510000 14.010000 -8.010000 4 749

751 CT -8.560000 14.940000 -6.390000 2 749 752 753 754

752 H1 -9.400000 14.990000 -7.080000 6 751

753 H1 -8.290000 15.950000 -6.090000 6 751

754 C -8.980000 14.160000 -5.180000 3 751 755 756

755 O -9.240000 14.740000 -4.120000 5 754

756 N -9.060000 12.820000 -5.300000 15 754 757 758

757 H -8.860000 12.390000 -6.200000 18 756

758 CT -9.500000 12.000000 -4.210000 16 756 759 760 770

759 H1 -10.460000 12.360000 -3.840000 20 758

760 CT -9.600000 10.550000 -4.580000 21 758 761 762 766

761 HC -8.620000 10.220000 -4.930000 22 760

762 CT -9.980000 9.750000 -3.330000 23 760 763 764 765

763 HC -10.140000 8.710000 -3.630000 24 762

764 HC -9.190000 9.750000 -2.580000 24 762

765 HC -10.900000 10.140000 -2.900000 24 762

766 CT -10.600000 10.410000 -5.740000 25 760 767 768 769

767 HC -10.410000 9.480000 -6.280000 26 766

768 HC -11.610000 10.370000 -5.320000 26 766

769 HC -10.640000 11.240000 -6.420000 26 766

770 C -8.490000 12.120000 -3.110000 17 758 771 772

771 O -8.850000 12.240000 -1.940000 19 770

772 N -7.200000 12.120000 -3.470000 15 770 773 774

773 H -7.010000 12.020000 -4.460000 18 772

774 CT -6.130000 12.190000 -2.520000 16 772 775 776 786

775 H1 -6.220000 11.400000 -1.780000 20 774

776 CT -4.790000 12.190000 -3.170000 21 774 777 778 782

777 HC -4.680000 13.060000 -3.810000 22 776

778 CT -3.710000 12.290000 -2.080000 23 776 779 780 781

779 HC -2.740000 11.940000 -2.430000 24 778

780 HC -3.640000 13.330000 -1.770000 24 778

781 HC -4.010000 11.790000 -1.170000 24 778

782 CT -4.700000 10.940000 -4.050000 25 776 783 784 785

783 HC -4.450000 11.400000 -4.990000 26 782

784 HC -3.890000 10.480000 -3.640000 26 782

785 HC -5.660000 10.590000 -4.150000 26 782

786 C -6.250000 13.490000 -1.800000 17 774 787 788

787 O -6.010000 13.570000 -0.590000 19 786

788 N -6.610000 14.560000 -2.520000 7 786 789 790

789 H -6.770000 14.480000 -3.520000 10 788

790 CT -6.670000 15.870000 -1.930000 8 788 791 792 796

791 H1 -5.730000 16.140000 -1.460000 12 790

792 CT -7.090000 16.960000 -2.930000 13 790 793 794 795

793 HC -7.070000 17.930000 -2.440000 14 792

794 HC -6.400000 16.980000 -3.770000 14 792

795 HC -8.100000 16.780000 -3.300000 14 792

796 C -7.680000 15.870000 -0.820000 9 790 797 798

797 O -7.430000 16.420000 0.250000 11 796

798 N -8.850000 15.240000 -1.030000 65 796 799 800

799 H -9.030000 14.790000 -1.920000 68 798

800 CT -9.840000 15.260000 0.010000 66 798 801 802 810

801 H1 -10.040000 16.290000 0.310000 70 800

802 CT -11.140000 14.610000 -0.400000 71 800 803 804 808

803 H1 -11.860000 14.700000 0.410000 72 802

804 CT -11.690000 15.330000 -1.630000 75 802 805 806 807

805 HC -12.650000 14.900000 -1.900000 76 804

806 HC -11.830000 16.390000 -1.410000 76 804

807 HC -11.020000 15.230000 -2.480000 76 804

808 OH -10.940000 13.230000 -0.680000 73 802 809

809 HO -11.750000 12.890000 -1.060000 74 808

810 C -9.320000 14.520000 1.200000 67 800 811 812

811 O -9.460000 14.980000 2.340000 69 810

812 N -8.670000 13.370000 0.970000 55 810 813 814

813 H -8.690000 13.040000 0.010000 58 812

814 CT -8.180000 12.550000 2.050000 56 812 815 816 821

815 H1 -9.000000 12.290000 2.720000 60 814

816 CT -7.520000 11.260000 1.550000 61 814 817 818 819

817 H1 -6.690000 11.490000 0.880000 62 816

818 H1 -8.270000 10.690000 1.000000 62 816

819 OH -7.050000 10.500000 2.660000 63 816 820

820 HO -6.860000 9.600000 2.380000 64 819

821 C -7.160000 13.310000 2.840000 57 814 822 823

822 O -7.200000 13.300000 4.070000 59 821

823 N -6.230000 14.000000 2.160000 138 821 824 825

824 H -6.280000 14.030000 1.150000 141 823

825 CT -5.180000 14.710000 2.830000 139 823 826 827 845

826 H1 -4.660000 14.030000 3.500000 143 825

827 CT -4.150000 15.340000 1.870000 144 825 828 829 830

828 HC -3.540000 16.050000 2.420000 145 827

829 HC -4.670000 15.900000 1.090000 145 827

830 C* -3.220000 14.350000 1.210000 146 827 831 844

831 CW -3.310000 12.990000 1.100000 147 830 832 833

832 H4 -4.190000 12.470000 1.410000 148 831

833 NA -2.210000 12.500000 0.440000 150 831 834 835

834 H -2.130000 11.540000 0.140000 151 833

835 CN -1.380000 13.550000 0.110000 152 833 836 844

836 CA -0.180000 13.580000 -0.540000 155 835 837 838

837 HA 0.280000 12.690000 -0.900000 156 836

838 CA 0.410000 14.810000 -0.720000 159 836 839 840

839 HA 1.370000 14.870000 -1.210000 160 838

840 CA -0.180000 15.970000 -0.260000 157 838 841 842

841 HA 0.190000 16.960000 -0.290000 158 840

842 CA -1.390000 15.940000 0.390000 153 840 843 844

843 HA -2.020000 16.800000 0.420000 154 842

844 CB -1.980000 14.720000 0.570000 149 830 835 842

845 C -5.730000 15.810000 3.670000 140 825 846 847

846 O -5.250000 16.050000 4.770000 142 845

847 N -6.750000 16.520000 3.160000 107 845 848 849

848 H -7.100000 16.320000 2.230000 110 847

849 CT -7.270000 17.630000 3.900000 108 847 850 851 865

850 H1 -6.480000 18.360000 4.090000 112 849

851 CT -8.460000 18.310000 3.200000 113 849 852 853 854

852 HC -9.300000 17.620000 3.100000 114 851

853 HC -8.160000 18.630000 2.200000 114 851

854 CA -8.840000 19.490000 4.030000 115 851 855 863

855 CA -9.710000 19.360000 5.090000 116 854 856 857

856 HA -10.180000 18.410000 5.280000 117 855

857 CA -10.060000 20.450000 5.850000 118 855 858 859

858 HA -10.930000 20.410000 6.470000 119 857

859 CA -9.540000 21.680000 5.560000 120 857 860 861

860 HA -9.860000 22.550000 6.120000 121 859

861 CA -8.670000 21.830000 4.500000 118 859 862 863

862 HA -8.270000 22.810000 4.260000 119 861

863 CA -8.320000 20.730000 3.740000 116 854 861 864

864 HA -7.630000 20.850000 2.910000 117 863

865 C -7.750000 17.100000 5.210000 109 849 866 867

866 O -7.470000 17.660000 6.270000 111 865

867 N -8.470000 15.970000 5.160000 27 865 868 869

868 H -8.650000 15.550000 4.250000 30 867

869 CT -9.000000 15.380000 6.350000 28 867 870 871 884

870 H1 -9.570000 16.120000 6.910000 32 869

871 CT -9.860000 14.130000 6.060000 33 869 872 873 874

872 HC -10.170000 13.700000 7.010000 34 871

873 HC -9.270000 13.390000 5.530000 34 871

874 CT -11.110000 14.430000 5.220000 35 871 875 876 880

875 HC -10.800000 14.790000 4.250000 36 874

876 CT -11.930000 13.160000 4.970000 37 874 877 878 879

877 HC -12.800000 13.400000 4.350000 38 876

878 HC -11.310000 12.430000 4.450000 38 876

879 HC -12.260000 12.750000 5.920000 38 876

880 CT -11.940000 15.570000 5.840000 39 874 881 882 883

881 HC -12.850000 15.720000 5.250000 40 880

882 HC -12.220000 15.320000 6.860000 40 880

883 HC -11.390000 16.510000 5.830000 40 880

884 C -7.870000 14.950000 7.240000 29 869 885 886

885 O -7.950000 15.100000 8.450000 31 884

886 N -6.780000 14.420000 6.640000 27 884 887 888

887 H -6.820000 14.300000 5.640000 30 886

888 CT -5.680000 13.870000 7.390000 28 886 889 890 903

889 H1 -6.070000 13.150000 8.110000 32 888

890 CT -4.600000 13.270000 6.470000 33 888 891 892 893

891 HC -4.200000 14.090000 5.880000 34 890

892 HC -4.910000 12.590000 5.700000 34 890

893 CT -3.370000 12.660000 7.190000 35 890 894 895 899

894 HC -2.840000 12.060000 6.450000 36 893

895 CT -2.390000 13.740000 7.690000 37 893 896 897 898

896 HC -2.130000 13.580000 8.730000 38 895

897 HC -1.500000 13.700000 7.070000 38 895

898 HC -2.730000 14.750000 7.600000 38 895

899 CT -3.820000 11.690000 8.290000 39 893 900 901 902

900 HC -3.020000 11.000000 8.540000 40 899

901 HC -3.980000 12.290000 9.170000 40 899

902 HC -4.760000 11.280000 8.110000 40 899

903 C -5.030000 14.900000 8.240000 29 888 904 905

904 O -4.710000 14.600000 9.390000 31 903

905 N -4.780000 16.120000 7.730000 122 903 906 907

906 H -5.070000 16.360000 6.790000 125 905

907 CT -4.140000 17.010000 8.640000 123 905 908 909 924

908 H1 -3.290000 16.490000 9.090000 127 907

909 CT -3.670000 18.380000 8.150000 128 907 910 911 912

910 HC -4.500000 19.030000 7.870000 129 909

911 HC -2.920000 18.340000 7.380000 129 909

912 CA -2.930000 18.880000 9.350000 130 909 913 922

913 CA -1.650000 18.440000 9.590000 131 912 914 915

914 HA -1.190000 17.720000 8.940000 132 913

915 CA -0.950000 18.880000 10.690000 133 913 916 917

916 HA 0.050000 18.510000 10.870000 134 915

917 C -1.530000 19.760000 11.570000 135 915 918 920

918 OH -0.830000 20.220000 12.700000 136 917 919

919 HO 0.100000 20.100000 12.500000 137 918

920 CA -2.810000 20.200000 11.340000 133 917 921 922

921 HA -3.250000 20.910000 12.010000 134 920

922 CA -3.510000 19.760000 10.230000 131 912 920 923

923 HA -4.510000 20.120000 10.050000 132 922

924 C -5.120000 17.290000 9.720000 124 907 925 926

925 O -4.760000 17.390000 10.890000 126 924

926 N -6.400000 17.400000 9.330000 27 924 927 928

927 H -6.670000 17.170000 8.380000 30 926

928 CT -7.380000 17.570000 10.340000 28 926 929 930 943

929 H1 -7.120000 18.400000 11.000000 32 928

930 CT -8.820000 17.710000 9.810000 33 928 931 932 933

931 HC -9.490000 17.750000 10.670000 34 930

932 HC -9.140000 16.870000 9.220000 34 930

933 CT -9.060000 18.980000 8.990000 35 930 934 935 939

934 HC -8.430000 18.950000 8.110000 36 933

935 CT -10.510000 19.070000 8.490000 37 933 936 937 938

936 HC -10.780000 20.120000 8.400000 38 935

937 HC -10.640000 18.480000 7.590000 38 935

938 HC -11.170000 18.670000 9.260000 38 935

939 CT -8.620000 20.230000 9.760000 39 933 940 941 942

940 HC -8.840000 21.110000 9.160000 40 939

941 HC -9.160000 20.300000 10.700000 40 939

942 HC -7.550000 20.220000 9.930000 40 939

943 C -7.310000 16.300000 11.130000 29 928 944 945

944 O -6.770000 15.290000 10.690000 31 943

945 N -7.780000 16.340000 12.370000 287 943 946 947

946 H -8.220000 17.180000 12.710000 290 945

947 CT -7.720000 15.160000 13.160000 288 945 948 949 967

948 H1 -6.710000 14.750000 13.150000 292 947

949 CT -8.190000 15.390000 14.610000 293 947 950 951 952

950 HC -9.200000 15.770000 14.580000 294 949

951 HC -7.550000 16.150000 15.060000 294 949

952 CT -8.130000 14.130000 15.480000 295 949 953 954 955

953 HC -7.130000 13.700000 15.420000 296 952

954 HC -8.840000 13.390000 15.110000 296 952

955 CT -8.450000 14.400000 16.950000 297 952 956 957 958

956 H1 -7.720000 15.110000 17.340000 298 955

957 H1 -8.400000 13.470000 17.520000 298 955

958 N2 -9.820000 14.980000 17.010000 299 955 959 960

959 H -10.390000 15.140000 16.180000 300 958

960 CA -10.270000 15.540000 18.170000 301 958 961 964

961 N2 -9.470000 15.560000 19.270000 302 960 962 963

962 H -9.810000 15.960000 20.120000 303 961

963 H -8.560000 15.140000 19.230000 303 961

964 N2 -11.520000 16.090000 18.230000 302 960 965 966

965 H -11.880000 16.560000 19.040000 303 964

966 H -11.970000 16.220000 17.320000 303 964

967 C -8.600000 14.130000 12.550000 289 947 968 969

968 O -8.300000 12.940000 12.600000 291 967

969 N -9.710000 14.570000 11.930000 7 967 970 971

970 H -9.880000 15.560000 11.900000 10 969

971 CT -10.670000 13.660000 11.380000 8 969 972 973 977

972 H1 -11.060000 13.040000 12.190000 12 971

973 CT -11.850000 14.350000 10.680000 13 971 974 975 976

974 HC -12.560000 13.610000 10.300000 14 973

975 HC -12.370000 15.000000 11.390000 14 973

976 HC -11.480000 14.960000 9.850000 14 973

977 C -10.000000 12.780000 10.380000 9 971 978 979

978 O -8.970000 13.110000 9.820000 11 977

979 N -10.570000 11.580000 10.180000 1 977 980 981

980 H -11.410000 11.350000 10.680000 4 979

981 CT -10.000000 10.670000 9.240000 2 979 982 983 984

982 H1 -9.450000 11.240000 8.500000 6 981

983 H1 -10.800000 10.050000 8.840000 6 981

984 C -9.020000 9.840000 9.990000 3 981 985 986

985 O -8.400000 8.920000 9.440000 5 984

986 N -8.830000 10.150000 11.280000 41 984 987 988

987 H -9.200000 11.010000 11.680000 44 986

988 CT -7.940000 9.360000 12.070000 42 986 989 990 1003

989 H1 -7.560000 8.520000 11.510000 46 988

990 CT -6.780000 10.130000 12.620000 47 988 991 992 996

991 HC -7.140000 10.960000 13.230000 48 990

992 CT -6.010000 9.210000 13.570000 51 990 993 994 995

993 HC -5.120000 9.710000 13.950000 52 992

994 HC -6.590000 9.030000 14.460000 52 992

995 HC -5.710000 8.290000 13.090000 52 992

996 CT -5.920000 10.700000 11.480000 49 990 997 998 999

997 HC -6.510000 11.360000 10.840000 50 996

998 HC -5.540000 9.870000 10.890000 50 996

999 CT -4.720000 11.490000 12.000000 53 996 1000 1001 1002

1000 HC -4.180000 11.940000 11.180000 54 999

1001 HC -5.070000 12.290000 12.650000 54 999

1002 HC -4.020000 10.850000 12.530000 54 999

1003 C -8.740000 8.870000 13.220000 43 988 1004 1005

1004 O -9.400000 9.650000 13.900000 45 1003

1005 N -8.730000 7.550000 13.460000 65 1003 1006 1007

1006 H -8.220000 6.920000 12.860000 68 1005

1007 CT -9.450000 7.100000 14.610000 66 1005 1008 1009 1017

1008 H1 -10.460000 7.510000 14.620000 70 1007

1009 CT -9.510000 5.610000 14.730000 71 1007 1010 1011 1015

1010 H1 -8.500000 5.200000 14.800000 72 1009

1011 CT -10.280000 5.250000 16.010000 75 1009 1012 1013 1014

1012 HC -10.330000 4.170000 16.110000 76 1011

1013 HC -9.750000 5.680000 16.820000 76 1011

1014 HC -11.290000 5.650000 15.980000 76 1011

1015 OH -10.150000 5.040000 13.600000 73 1009 1016

1016 HO -10.410000 4.140000 13.830000 74 1015

1017 C -8.650000 7.620000 15.740000 67 1007 1018 1019

1018 O -7.450000 7.350000 15.830000 69 1017

1019 N -9.300000 8.400000 16.640000 27 1017 1020 1021

1020 H -10.260000 8.660000 16.470000 30 1019

1021 CT -8.570000 8.980000 17.730000 28 1019 1022 1023 1036

1022 H1 -7.750000 9.610000 17.370000 32 1021

1023 CT -9.480000 9.750000 18.700000 33 1021 1024 1025 1026

1024 HC -8.870000 10.100000 19.530000 34 1023

1025 HC -10.250000 9.090000 19.110000 34 1023

1026 CT -10.180000 10.960000 18.070000 35 1023 1027 1028 1032

1027 HC -9.410000 11.590000 17.620000 36 1026

1028 CT -11.130000 10.530000 16.940000 37 1026 1029 1030 1031

1029 HC -11.800000 11.360000 16.720000 38 1028

1030 HC -10.600000 10.360000 16.010000 38 1028

1031 HC -11.750000 9.690000 17.240000 38 1028

1032 CT -10.890000 11.820000 19.130000 39 1026 1033 1034 1035

1033 HC -11.360000 12.680000 18.660000 40 1032

1034 HC -11.660000 11.230000 19.630000 40 1032

1035 HC -10.170000 12.170000 19.870000 40 1032

1036 C -8.030000 7.820000 18.470000 29 1021 1037 1038

1037 O -6.860000 7.760000 18.810000 31 1036

1038 N -8.920000 6.840000 18.710000 27 1036 1039 1040

1039 H -9.890000 6.930000 18.450000 30 1038

1040 CT -8.470000 5.650000 19.340000 28 1038 1041 1042 1055

1041 H1 -7.470000 5.410000 18.980000 32 1040

1042 CT -8.430000 5.720000 20.890000 33 1040 1043 1044 1045

1043 HC -7.770000 6.550000 21.160000 34 1042

1044 HC -7.960000 4.810000 21.280000 34 1042

1045 CT -9.780000 5.920000 21.610000 35 1042 1046 1047 1051

1046 HC -10.330000 6.690000 21.070000 36 1045

1047 CT -10.650000 4.650000 21.590000 37 1045 1048 1049 1050

1048 HC -11.560000 4.930000 22.120000 38 1047

1049 HC -11.050000 4.100000 20.780000 38 1047

1050 HC -10.140000 4.010000 22.270000 38 1047

1051 CT -9.560000 6.460000 23.030000 39 1045 1052 1053 1054

1052 HC -10.520000 6.620000 23.520000 40 1051

1053 HC -8.970000 5.760000 23.620000 40 1051

1054 HC -9.040000 7.420000 22.990000 40 1051

1055 C -9.400000 4.580000 18.920000 29 1040 1056 1057

1056 O -10.620000 4.780000 18.850000 31 1055

1057 N -8.830000 3.430000 18.550000 210 1055 1058 1059

1058 H -7.830000 3.410000 18.400000 213 1057

1059 CT -9.600000 2.290000 18.200000 211 1057 1060 1061 1067

1060 H1 -10.400000 2.550000 17.500000 215 1059

1061 CT -8.730000 1.160000 17.620000 216 1059 1062 1063 1064

1062 HC -8.090000 0.740000 18.390000 217 1061

1063 HC -8.100000 1.570000 16.830000 217 1061

1064 C -9.630000 0.090000 17.030000 218 1061 1065 1066

1065 O2 -10.870000 0.180000 17.210000 219 1064

1066 O2 -9.080000 -0.850000 16.390000 219 1064

1067 C -10.230000 1.790000 19.460000 212 1059 1068 1069

1068 O -11.330000 1.250000 19.460000 214 1067

1069 N -9.530000 2.020000 20.590000 1 1067 1070 1071

1070 H -8.790000 2.700000 20.510000 4 1069

1071 CT -9.900000 1.480000 21.860000 2 1069 1072 1073 1074

1072 H1 -10.810000 0.890000 21.790000 6 1071

1073 H1 -10.000000 2.170000 22.680000 6 1071

1074 C -8.760000 0.580000 22.200000 3 1071 1075 1076

1075 O -8.500000 0.270000 23.360000 5 1074

1076 N -8.070000 0.130000 21.140000 7 1074 1077 1078

1077 H -8.510000 0.270000 20.240000 10 1076

1078 CT -6.850000 -0.610000 21.230000 8 1076 1079 1080 1084

1079 H1 -6.970000 -1.460000 21.900000 12 1078

1080 CT -6.300000 -1.050000 19.860000 13 1078 1081 1082 1083

1081 HC -5.300000 -1.430000 19.970000 14 1080

1082 HC -6.960000 -1.800000 19.410000 14 1080

1083 HC -6.240000 -0.190000 19.190000 14 1080

1084 C -5.870000 0.350000 21.820000 9 1078 1085 1086

1085 O -4.950000 -0.040000 22.540000 11 1084

1086 N -6.040000 1.640000 21.490000 1 1084 1087 1088

1087 H -6.880000 1.880000 20.980000 4 1086

1088 CT -5.190000 2.660000 22.020000 2 1086 1089 1090 1091

1089 H1 -4.520000 2.280000 22.790000 6 1088

1090 H1 -5.800000 3.440000 22.470000 6 1088

1091 C -4.370000 3.270000 20.930000 3 1088 1092 1093

1092 O -3.680000 4.260000 21.160000 5 1091

1093 N -4.410000 2.720000 19.700000 161 1091 1094 1095

1094 H -5.050000 1.970000 19.480000 164 1093

1095 CT -3.600000 3.360000 18.710000 162 1093 1096 1097 1109

1096 H1 -2.940000 4.080000 19.150000 166 1095

1097 CT -2.540000 2.420000 18.110000 167 1095 1098 1099 1100

1098 HC -2.030000 2.910000 17.280000 168 1097

1099 HC -3.010000 1.510000 17.730000 168 1097

1100 CC -1.530000 2.060000 19.160000 169 1097 1101 1107

1101 NA -0.450000 2.840000 19.510000 170 1100 1102 1103

1102 H -0.230000 3.760000 19.130000 171 1101

1103 CR 0.190000 2.210000 20.520000 174 1101 1104 1105

1104 H5 0.840000 2.730000 21.190000 175 1103

1105 NA -0.400000 1.070000 20.850000 176 1103 1106 1107

1106 H -0.150000 0.450000 21.620000 177 1105

1107 CW -1.480000 0.980000 20.000000 172 1100 1105 1108

1108 H4 -2.210000 0.180000 20.000000 173 1107

1109 C -4.450000 3.940000 17.630000 163 1095 1110 1111

1110 O -5.500000 3.410000 17.260000 165 1109

1111 N -4.030000 5.100000 17.200000 96 1109 1112 1121

1112 CT -3.430000 6.030000 18.140000 105 1111 1113 1114 1115

1113 H1 -2.340000 6.010000 18.050000 106 1112

1114 H1 -3.750000 5.870000 19.170000 106 1112

1115 CT -3.950000 7.420000 17.750000 103 1112 1116 1117 1118

1116 HC -3.140000 8.150000 17.830000 104 1115

1117 HC -4.720000 7.740000 18.410000 104 1115

1118 CT -4.360000 7.260000 16.280000 101 1115 1119 1120 1121

1119 HC -3.510000 7.480000 15.650000 102 1118

1120 HC -5.160000 7.950000 16.030000 102 1118

1121 CT -4.760000 5.790000 16.170000 97 1111 1118 1122 1123

1122 H1 -5.820000 5.710000 16.400000 100 1121

1123 C -4.520000 5.220000 14.820000 98 1121 1124 1125

1124 O -3.400000 4.840000 14.520000 99 1123

1125 N -5.560000 5.160000 13.970000 15 1123 1126 1127

1126 H -6.450000 5.550000 14.250000 18 1125

1127 CT -5.360000 4.660000 12.650000 16 1125 1128 1129 1139

1128 H1 -4.320000 4.410000 12.450000 20 1127

1129 CT -6.200000 3.450000 12.350000 21 1127 1130 1131 1135

1130 HC -7.250000 3.700000 12.480000 22 1129

1131 CT -5.990000 3.050000 10.880000 23 1129 1132 1133 1134

1132 HC -6.520000 2.110000 10.710000 24 1131

1133 HC -6.430000 3.780000 10.200000 24 1131

1134 HC -4.930000 2.910000 10.670000 24 1131

1135 CT -5.850000 2.340000 13.360000 25 1129 1136 1137 1138

1136 HC -6.440000 1.450000 13.140000 26 1135

1137 HC -4.800000 2.090000 13.270000 26 1135

1138 HC -6.080000 2.650000 14.380000 26 1135

1139 C -5.780000 5.750000 11.720000 17 1127 1140 1141

1140 O -6.810000 6.380000 11.940000 19 1139

1141 N -4.990000 6.000000 10.660000 649 1139 1142 1143

1142 H -4.150000 5.460000 10.510000 652 1141

1143 CT -5.380000 7.000000 9.700000 650 1141 1144 1145 1152

1144 H1 -5.990000 7.760000 10.180000 654 1143

1145 CT -4.190000 7.650000 8.970000 655 1143 1146 1147 1148

1146 HC -3.650000 6.950000 8.330000 656 1145

1147 HC -3.490000 8.070000 9.670000 656 1145

1148 C -4.730000 8.830000 8.170000 657 1145 1149 1150

1149 O -5.810000 9.360000 8.550000 658 1148

1150 OH -4.080000 9.200000 7.160000 659 1148 1151

1151 HO -3.140000 9.240000 7.350000 660 1150

1152 C -6.180000 6.270000 8.680000 651 1143 1153 1154

1153 O -5.650000 5.810000 7.670000 653 1152

1154 N -7.500000 6.150000 8.930000 41 1152 1155 1156

1155 H -7.860000 6.650000 9.740000 44 1154

1156 CT -8.430000 5.420000 8.110000 42 1154 1157 1158 1171

1157 H1 -8.150000 4.390000 8.100000 46 1156

1158 CT -9.810000 5.370000 8.690000 47 1156 1159 1160 1164

1159 HC -10.440000 4.780000 8.020000 48 1158

1160 CT -9.730000 4.620000 10.030000 51 1158 1161 1162 1163

1161 HC -10.730000 4.420000 10.410000 52 1160

1162 HC -9.230000 3.660000 9.920000 52 1160

1163 HC -9.200000 5.210000 10.780000 52 1160

1164 CT -10.410000 6.780000 8.800000 49 1158 1165 1166 1167

1165 HC -10.520000 7.310000 7.860000 50 1164

1166 HC -9.860000 7.300000 9.550000 50 1164

1167 CT -11.820000 6.750000 9.390000 53 1164 1168 1169 1170

1168 HC -12.230000 7.760000 9.400000 54 1167

1169 HC -12.470000 6.120000 8.780000 54 1167

1170 HC -11.810000 6.390000 10.420000 54 1167

1171 C -8.530000 6.050000 6.760000 43 1156 1172 1173

1172 O -8.670000 5.370000 5.750000 45 1171

1173 N -8.460000 7.390000 6.700000 41 1171 1174 1175

1174 H -8.340000 7.920000 7.560000 44 1173

1175 CT -8.660000 8.090000 5.470000 42 1173 1176 1177 1190

1176 H1 -9.660000 7.860000 5.090000 46 1175

1177 CT -8.520000 9.580000 5.580000 47 1175 1178 1179 1183

1178 HC -8.600000 10.010000 4.580000 48 1177

1179 CT -9.720000 10.120000 6.370000 51 1177 1180 1181 1182

1180 HC -9.690000 11.190000 6.540000 52 1179

1181 HC -10.620000 9.950000 5.770000 52 1179

1182 HC -9.820000 9.480000 7.220000 52 1179

1183 CT -7.140000 9.950000 6.140000 49 1177 1184 1185 1186

1184 HC -6.280000 9.550000 5.620000 50 1183

1185 HC -7.320000 9.560000 7.110000 50 1183

1186 CT -6.970000 11.460000 6.270000 53 1183 1187 1188 1189

1187 HC -6.290000 11.520000 7.060000 54 1186

1188 HC -6.560000 11.660000 5.310000 54 1186

1189 HC -7.860000 12.010000 6.540000 54 1186

1190 C -7.680000 7.620000 4.440000 43 1175 1191 1192

1191 O -8.020000 7.540000 3.260000 45 1190

1192 N -6.430000 7.320000 4.830000 287 1190 1193 1194

1193 H -6.200000 7.420000 5.810000 290 1192

1194 CT -5.450000 6.920000 3.860000 288 1192 1195 1196 1214

1195 H1 -5.320000 7.700000 3.110000 292 1194

1196 CT -4.100000 6.590000 4.500000 293 1194 1197 1198 1199

1197 HC -3.500000 5.990000 3.820000 294 1196

1198 HC -4.260000 6.000000 5.400000 294 1196

1199 CT -3.330000 7.850000 4.860000 295 1196 1200 1201 1202

1200 HC -2.690000 7.670000 5.720000 296 1199

1201 HC -4.080000 8.610000 5.010000 296 1199

1202 CT -2.550000 8.390000 3.670000 297 1199 1203 1204 1205

1203 H1 -3.190000 8.440000 2.790000 298 1202

1204 H1 -1.730000 7.720000 3.430000 298 1202

1205 N2 -2.070000 9.750000 4.030000 299 1202 1206 1207

1206 H -2.550000 10.550000 3.630000 300 1205

1207 CA -0.960000 9.900000 4.800000 301 1205 1208 1211

1208 N2 -0.360000 8.800000 5.360000 302 1207 1209 1210

1209 H 0.280000 9.100000 6.110000 303 1208

1210 H -0.760000 7.890000 5.320000 303 1208

1211 N2 -0.450000 11.140000 5.010000 302 1207 1212 1213

1212 H 0.210000 11.190000 5.780000 303 1211

1213 H -0.980000 11.960000 4.750000 303 1211

1214 C -5.920000 5.700000 3.140000 289 1194 1215 1216

1215 O -5.780000 5.600000 1.920000 291 1214

1216 N -6.480000 4.720000 3.870000 194 1214 1217 1218

1217 H -6.600000 4.870000 4.860000 197 1216

1218 CT -6.900000 3.490000 3.270000 195 1216 1219 1220 1231

1219 H1 -6.070000 3.010000 2.740000 199 1218

1220 CT -7.378573 2.517037 4.364411 200 1218 1221 1222 1223

1221 HC -7.393102 1.512665 3.941428 201 1220

1222 HC -8.399335 2.782759 4.638654 201 1220

1223 CC -6.582712 2.474851 5.649799 202 1220 1224 1229

1224 NB -5.246758 2.836569 5.812444 203 1223 1225

1225 CR -4.985109 2.718849 7.119138 206 1224 1226 1227

1226 H5 -4.028706 2.954371 7.565778 207 1225

1227 NA -6.073247 2.283728 7.777349 208 1225 1228 1229

1228 H -6.148053 2.119987 8.769672 209 1227

1229 CW -7.094812 2.140710 6.868399 204 1223 1227 1230

1230 H4 -8.115344 1.848469 7.071668 205 1229

1231 C -8.010000 3.750000 2.310000 196 1218 1232 1233

1232 O -8.060000 3.170000 1.230000 198 1231

1233 N -8.960000 4.640000 2.690000 27 1231 1234 1235

1234 H -8.880000 5.060000 3.610000 30 1233

1235 CT -10.090000 4.900000 1.860000 28 1233 1236 1237 1250

1236 H1 -10.590000 3.950000 1.650000 32 1235

1237 CT -11.080000 5.910000 2.500000 33 1235 1238 1239 1240

1238 HC -10.580000 6.870000 2.660000 34 1237

1239 HC -11.360000 5.530000 3.480000 34 1237

1240 CT -12.380000 6.150000 1.700000 35 1237 1241 1242 1246

1241 HC -13.020000 6.770000 2.330000 36 1240

1242 CT -12.140000 6.940000 0.410000 37 1240 1243 1244 1245

1243 HC -13.110000 7.310000 0.080000 38 1242

1244 HC -11.530000 7.820000 0.630000 38 1242

1245 HC -11.770000 6.430000 -0.410000 38 1242

1246 CT -13.130000 4.830000 1.470000 39 1240 1247 1248 1249

1247 HC -14.090000 5.040000 1.010000 40 1246

1248 HC -12.570000 4.170000 0.810000 40 1246

1249 HC -13.310000 4.340000 2.430000 40 1246

1250 C -9.590000 5.480000 0.580000 29 1235 1251 1252

1251 O -10.070000 5.130000 -0.500000 31 1250

1252 N -8.600000 6.380000 0.670000 661 1250 1253 1254

1253 H -8.280000 6.640000 1.600000 664 1252

1254 CT -8.050000 7.010000 -0.500000 662 1252 1255 1256 1266

1255 H1 -8.830000 7.540000 -1.040000 666 1254

1256 CT -6.971546 8.001397 -0.061228 667 1254 1257 1258 1259

1257 HC -6.224303 7.493390 0.539180 668 1256

1258 HC -7.426600 8.774121 0.547007 668 1256

1259 CT -6.265360 8.628709 -1.262328 669 1256 1260 1261 1262

1260 HC -6.907397 9.391515 -1.693886 670 1259

1261 HC -6.049222 7.864030 -1.997903 670 1259

1262 C -4.917699 9.210428 -0.921310 671 1259 1263 1264

1263 O -4.752033 9.705250 0.175481 672 1262

1264 OH -3.877977 8.725591 -1.618644 673 1262 1265

1265 HO -3.124186 8.904944 -1.039513 674 1264

1266 C -7.450000 5.970000 -1.390000 663 1254 1267 1268

1267 O -7.700000 5.930000 -2.590000 665 1266

1268 N -6.660000 5.060000 -0.790000 138 1266 1269 1270

1269 H -6.490000 5.140000 0.210000 141 1268

1270 CT -5.930000 4.060000 -1.510000 139 1268 1271 1272 1290

1271 H1 -5.320000 4.520000 -2.280000 143 1270

1272 CT -4.967740 3.312122 -0.588442 144 1270 1273 1274 1275

1273 HC -4.581509 2.463097 -1.149681 145 1272

1274 HC -5.508081 2.927166 0.276696 145 1272

1275 C* -3.770314 4.083310 -0.119870 146 1272 1276 1289

1276 CW -3.351877 5.280172 -0.595192 147 1275 1277 1278

1277 H4 -3.841085 5.860129 -1.366941 148 1276

1278 NA -2.169887 5.632811 0.018796 150 1276 1279 1280

1279 H -1.643537 6.456373 -0.253986 151 1278

1280 CN -1.774376 4.691669 0.941548 152 1278 1281 1289

1281 CA -0.663501 4.594448 1.790209 155 1280 1282 1283

1282 HA 0.094285 5.359781 1.788469 156 1281

1283 CA -0.536191 3.463893 2.613255 159 1281 1284 1285

1284 HA 0.320815 3.354656 3.264163 160 1283

1285 CA -1.505421 2.446502 2.565092 157 1283 1286 1287

1286 HA -1.389004 1.563892 3.180566 158 1285

1287 CA -2.619496 2.559700 1.713935 153 1285 1288 1289

1288 HA -3.356047 1.771281 1.690479 154 1287

1289 CB -2.782540 3.684584 0.877780 149 1275 1280 1287

1290 C -6.880000 3.140000 -2.210000 140 1270 1291 1292

1291 O -6.620000 2.710000 -3.330000 142 1290

1292 N -8.030000 2.820000 -1.580000 7 1290 1293 1294

1293 H -8.170000 3.120000 -0.620000 10 1292

1294 CT -8.920000 1.880000 -2.190000 8 1292 1295 1296 1300

1295 H1 -8.550000 0.900000 -2.380000 12 1294

1296 CT -10.190000 1.640000 -1.350000 13 1294 1297 1298 1299

1297 HC -10.840000 0.930000 -1.860000 14 1296

1298 HC -9.910000 1.230000 -0.380000 14 1296

1299 HC -10.730000 2.580000 -1.190000 14 1296

1300 C -9.350000 2.410000 -3.520000 9 1294 1301 1302

1301 O -9.350000 1.670000 -4.500000 11 1300

1302 N -9.740000 3.690000 -3.590000 161 1300 1303 1304

1303 H -9.660000 4.240000 -2.750000 164 1302

1304 CT -10.170000 4.260000 -4.840000 162 1302 1305 1306 1318

1305 H1 -10.820000 3.550000 -5.360000 166 1304

1306 CT -10.930000 5.590000 -4.670000 167 1304 1307 1308 1309

1307 HC -11.000000 6.090000 -5.640000 168 1306

1308 HC -10.370000 6.250000 -4.000000 168 1306

1309 CC -12.320000 5.390000 -4.140000 169 1306 1310 1316

1310 NA -13.410000 5.090000 -4.930000 170 1309 1311 1312

1311 H -13.390000 4.990000 -5.940000 171 1310

1312 CR -14.480000 5.000000 -4.110000 174 1310 1313 1314

1313 H5 -15.490000 4.790000 -4.460000 175 1312

1314 NA -14.160000 5.220000 -2.840000 176 1312 1315 1316

1315 H -14.770000 5.220000 -2.030000 177 1314

1316 CW -12.800000 5.470000 -2.870000 172 1309 1314 1317

1317 H4 -12.140000 5.600000 -2.050000 173 1316

1318 C -9.030000 4.520000 -5.780000 163 1304 1319 1320

1319 O -9.110000 4.170000 -6.960000 165 1318

1320 N -7.950000 5.150000 -5.290000 649 1318 1321 1322

1321 H -7.970000 5.400000 -4.310000 652 1320

1322 CT -6.870000 5.620000 -6.120000 650 1320 1323 1324 1331

1323 H1 -7.290000 6.180000 -6.960000 654 1322

1324 CT -6.052515 6.642526 -5.350909 655 1322 1325 1326 1327

1325 HC -5.124212 6.818801 -5.883520 656 1324

1326 HC -5.810649 6.275229 -4.354112 656 1324

1327 C -6.849407 7.945232 -5.276583 657 1324 1328 1329

1328 O -7.768103 8.189809 -6.036108 658 1327

1329 OH -6.883720 8.574381 -4.112027 659 1327 1330

1330 HO -7.507057 9.244925 -4.407909 660 1329

1331 C -6.060000 4.520000 -6.740000 651 1322 1332 1333

1332 O -5.710000 4.600000 -7.920000 653 1331

1333 N -5.750000 3.450000 -5.980000 232 1331 1334 1335

1334 H -6.150000 3.340000 -5.050000 235 1333

1335 CT -4.850000 2.420000 -6.420000 233 1333 1336 1337 1346

1336 H1 -3.870000 2.840000 -6.640000 237 1335

1337 CT -4.594446 1.432597 -5.270611 238 1335 1338 1339 1340

1338 HC -5.419348 0.724901 -5.176903 239 1337

1339 HC -4.542110 2.030108 -4.362431 239 1337

1340 CT -3.239912 0.709618 -5.295190 240 1337 1341 1342 1343

1341 HC -3.014887 0.396759 -4.272489 241 1340

1342 HC -2.468334 1.418861 -5.592896 241 1340

1343 C -3.162865 -0.521404 -6.194836 242 1340 1344 1345

1344 O2 -4.073315 -0.730353 -7.023044 243 1343

1345 O2 -2.152992 -1.250439 -6.073650 243 1343

1346 C -5.400000 1.800000 -7.670000 234 1335 1347 1348

1347 O -4.660000 1.580000 -8.640000 236 1346

1348 N -6.710000 1.480000 -7.690000 7 1346 1349 1350

1349 H -7.250000 1.610000 -6.850000 10 1348

1350 CT -7.310000 0.850000 -8.830000 8 1348 1351 1352 1356

1351 H1 -6.770000 -0.080000 -9.060000 12 1350

1352 CT -8.800000 0.550000 -8.630000 13 1350 1353 1354 1355

1353 HC -9.200000 0.070000 -9.530000 14 1352

1354 HC -8.920000 -0.130000 -7.790000 14 1352

1355 HC -9.350000 1.470000 -8.430000 14 1352

1356 C -7.190000 1.770000 -10.010000 9 1350 1357 1358

1357 O -6.920000 1.340000 -11.120000 11 1356

1358 N -7.400000 3.080000 -9.770000 220 1356 1359 1360

1359 H -7.580000 3.370000 -8.820000 223 1358

1360 CT -7.380000 4.060000 -10.820000 221 1358 1361 1362 1370

1361 H1 -8.060000 3.760000 -11.620000 225 1360

1362 CT -7.700000 5.470000 -10.310000 226 1360 1363 1364 1365

1363 HC -7.580000 6.190000 -11.110000 227 1362

1364 HC -7.060000 5.780000 -9.490000 227 1362

1365 C -9.150000 5.480000 -9.860000 228 1362 1366 1367

1366 O -9.490000 6.040000 -8.820000 229 1365

1367 N -10.040000 4.830000 -10.660000 230 1365 1368 1369

1368 H -10.990000 4.880000 -10.350000 231 1367

1369 H -9.770000 4.410000 -11.530000 231 1367

1370 C -6.010000 4.100000 -11.450000 222 1360 1371 1372

1371 O -5.890000 4.190000 -12.670000 224 1370

1372 N -4.930000 4.030000 -10.640000 27 1370 1373 1374

1373 H -5.060000 3.920000 -9.640000 30 1372

1374 CT -3.620000 4.070000 -11.240000 28 1372 1375 1376 1389

1375 H1 -3.540000 4.920000 -11.910000 32 1374

1376 CT -2.569270 4.155314 -10.111544 33 1374 1377 1378 1379

1377 HC -1.575899 4.021142 -10.541545 34 1376

1378 HC -2.738740 3.325601 -9.423396 34 1376

1379 CT -2.617211 5.486582 -9.326837 35 1376 1380 1381 1385

1380 HC -3.624730 5.898593 -9.347789 36 1379

1381 CT -2.228477 5.318687 -7.857533 37 1379 1382 1383 1384

1382 HC -2.922725 4.631447 -7.382759 38 1381

1383 HC -1.215320 4.926321 -7.781528 38 1381

1384 HC -2.282768 6.284639 -7.364477 38 1381

1385 CT -1.660912 6.513360 -9.927284 39 1379 1386 1387 1388

1386 HC -1.822268 7.490325 -9.476341 40 1385

1387 HC -0.633139 6.208177 -9.737778 40 1385

1388 HC -1.806969 6.585949 -11.001260 40 1385

1389 C -3.440000 2.860000 -12.110000 29 1374 1390 1391

1390 O -2.890000 2.950000 -13.210000 31 1389

1391 N -3.890000 1.690000 -11.640000 7 1389 1392 1393

1392 H -4.270000 1.630000 -10.700000 10 1391

1393 CT -3.730000 0.470000 -12.380000 8 1391 1394 1395 1399

1394 H1 -2.680000 0.310000 -12.610000 12 1393

1395 CT -4.270000 -0.770000 -11.640000 13 1393 1396 1397 1398

1396 HC -4.080000 -1.670000 -12.230000 14 1395

1397 HC -3.770000 -0.860000 -10.680000 14 1395

1398 HC -5.340000 -0.680000 -11.460000 14 1395

1399 C -4.490000 0.580000 -13.660000 9 1393 1400 1401

1400 O -4.050000 0.100000 -14.710000 11 1399

1401 N -5.670000 1.210000 -13.600000 122 1399 1402 1403

1402 H -6.000000 1.480000 -12.680000 125 1401

1403 CT -6.540000 1.360000 -14.740000 123 1401 1404 1405 1420

1404 H1 -6.760000 0.380000 -15.170000 127 1403

1405 CT -7.840000 2.100000 -14.370000 128 1403 1406 1407 1408

1406 HC -7.630000 3.100000 -14.000000 129 1405

1407 HC -8.360000 1.550000 -13.580000 129 1405

1408 CA -8.720000 2.190000 -15.570000 130 1405 1409 1418

1409 CA -9.460000 1.100000 -15.980000 131 1408 1410 1411

1410 HA -9.640000 0.350000 -15.240000 132 1409

1411 CA -10.280000 1.180000 -17.080000 133 1409 1412 1413

1412 HA -10.900000 0.340000 -17.360000 134 1411

1413 C -10.380000 2.360000 -17.780000 135 1411 1414 1416

1414 OH -11.220000 2.440000 -18.900000 136 1413 1415

1415 HO -11.500000 1.570000 -19.190000 137 1414

1416 CA -9.650000 3.460000 -17.370000 133 1413 1417 1418

1417 HA -9.870000 4.410000 -17.810000 134 1416

1418 CA -8.830000 3.370000 -16.270000 131 1408 1416 1419

1419 HA -8.280000 4.240000 -15.950000 132 1418

1420 C -5.800000 2.160000 -15.750000 124 1403 1421 1422

1421 O -5.860000 1.890000 -16.950000 126 1420

1422 N -5.060000 3.190000 -15.300000 107 1420 1423 1424

1423 H -5.040000 3.370000 -14.300000 110 1422

1424 CT -4.370000 4.050000 -16.200000 108 1422 1425 1426 1440

1425 H1 -5.090000 4.450000 -16.920000 112 1424

1426 CT -3.620000 5.180000 -15.490000 113 1424 1427 1428 1429

1427 HC -2.640000 4.870000 -15.130000 114 1426

1428 HC -4.210000 5.580000 -14.670000 114 1426

1429 CA -3.520000 6.160000 -16.590000 115 1426 1430 1438

1430 CA -2.520000 6.050000 -17.520000 116 1429 1431 1432

1431 HA -1.760000 5.280000 -17.450000 117 1430

1432 CA -2.460000 6.950000 -18.550000 118 1430 1433 1434

1433 HA -1.700000 6.720000 -19.250000 119 1432

1434 CA -3.400000 7.950000 -18.630000 120 1432 1435 1436

1435 HA -3.510000 8.160000 -19.660000 121 1434

1436 CA -4.410000 8.050000 -17.710000 118 1434 1437 1438

1437 HA -5.300000 8.450000 -17.690000 119 1436

1438 CA -4.470000 7.150000 -16.690000 116 1429 1436 1439

1439 HA -5.290000 7.180000 -15.990000 117 1438

1440 C -3.350000 3.240000 -16.970000 109 1424 1441 1442

1441 O -3.190000 3.420000 -18.180000 111 1440

1442 N -2.620000 2.350000 -16.270000 27 1440 1443 1444

1443 H -2.810000 2.250000 -15.280000 30 1442

1444 CT -1.600000 1.540000 -16.890000 28 1442 1445 1446 1459

1445 H1 -0.870000 2.160000 -17.410000 32 1444

1446 CT -0.910000 0.570000 -15.910000 33 1444 1447 1448 1449

1447 HC -0.160000 0.000000 -16.470000 34 1446

1448 HC -1.630000 -0.140000 -15.510000 34 1446

1449 CT -0.210000 1.240000 -14.720000 35 1446 1450 1451 1455

1450 HC -0.970000 1.700000 -14.100000 36 1449

1451 CT 0.500000 0.220000 -13.830000 37 1449 1452 1453 1454

1452 HC 0.930000 0.720000 -12.980000 38 1451

1453 HC -0.220000 -0.520000 -13.480000 38 1451

1454 HC 1.280000 -0.290000 -14.400000 38 1451

1455 CT 0.720000 2.360000 -15.190000 39 1449 1456 1457 1458

1456 HC 1.220000 2.750000 -14.310000 40 1455

1457 HC 1.460000 1.960000 -15.880000 40 1455

1458 HC 0.150000 3.160000 -15.660000 40 1455

1459 C -2.240000 0.640000 -17.900000 29 1444 1460 1461

1460 O -1.720000 0.460000 -19.010000 31 1459

1461 N -3.380000 0.040000 -17.520000 1 1459 1462 1463

1462 H -3.710000 0.210000 -16.580000 4 1461

1463 CT -4.080000 -0.900000 -18.340000 2 1461 1464 1465 1466

1464 H1 -4.900000 -1.390000 -17.820000 6 1463

1465 H1 -3.370000 -1.590000 -18.750000 6 1463

1466 C -4.540000 -0.260000 -19.600000 3 1463 1467 1468

1467 O -4.520000 -0.880000 -20.670000 5 1466

1468 N -5.010000 1.000000 -19.520000 27 1466 1469 1470

1469 H -5.060000 1.430000 -18.610000 30 1468

1470 CT -5.500000 1.710000 -20.670000 28 1468 1471 1472 1485

1471 H1 -6.270000 1.120000 -21.170000 32 1470

1472 CT -6.000000 3.140000 -20.340000 33 1470 1473 1474 1475

1473 HC -6.010000 3.730000 -21.260000 34 1472

1474 HC -5.300000 3.620000 -19.660000 34 1472

1475 CT -7.410000 3.210000 -19.730000 35 1472 1476 1477 1481

1476 HC -7.440000 2.610000 -18.830000 36 1475

1477 CT -7.770000 4.650000 -19.350000 37 1475 1478 1479 1480

1478 HC -8.830000 4.830000 -19.430000 38 1477

1479 HC -7.360000 4.900000 -18.370000 38 1477

1480 HC -7.330000 5.320000 -20.090000 38 1477

1481 CT -8.440000 2.620000 -20.700000 39 1475 1482 1483 1484

1482 HC -9.440000 2.850000 -20.390000 40 1481

1483 HC -8.300000 3.050000 -21.690000 40 1481

1484 HC -8.330000 1.540000 -20.770000 40 1481

1485 C -4.400000 1.900000 -21.650000 29 1470 1486 1487

1486 O -4.600000 1.750000 -22.860000 31 1485

1487 N -3.200000 2.240000 -21.160000 27 1485 1488 1489

1488 H -3.090000 2.340000 -20.160000 30 1487

1489 CT -2.100000 2.490000 -22.040000 28 1487 1490 1491 1504

1490 H1 -2.380000 3.200000 -22.820000 32 1489

1491 CT -0.820000 2.890000 -21.280000 33 1489 1492 1493 1494

1492 HC -0.280000 3.150000 -22.140000 34 1491

1493 HC -0.480000 2.090000 -20.630000 34 1491

1494 CT -0.920000 4.240000 -20.550000 35 1491 1495 1496 1500

1495 HC -1.690000 4.160000 -19.810000 36 1494

1496 CT 0.390000 4.570000 -19.810000 37 1494 1497 1498 1499

1497 HC 0.190000 5.390000 -19.120000 38 1496

1498 HC 0.720000 3.710000 -19.230000 38 1496

1499 HC 1.150000 4.930000 -20.480000 38 1496

1500 CT -1.330000 5.360000 -21.510000 39 1494 1501 1502 1503

1501 HC -1.920000 6.100000 -21.030000 40 1500

1502 HC -0.480000 5.690000 -22.110000 40 1500

1503 HC -2.070000 5.030000 -22.230000 40 1500

1504 C -1.790000 1.240000 -22.780000 29 1489 1505 1506

1505 O -1.580000 1.260000 -23.990000 31 1504

1506 N -1.780000 0.110000 -22.050000 65 1504 1507 1508

1507 H -2.030000 0.180000 -21.070000 68 1506

1508 CT -1.450000 -1.190000 -22.550000 66 1506 1509 1510 1518

1509 H1 -0.540000 -1.140000 -23.140000 70 1508

1510 CT -1.260000 -2.220000 -21.470000 71 1508 1511 1512 1516

1511 H1 -0.940000 -3.160000 -21.920000 72 1510

1512 CT -0.180000 -1.720000 -20.500000 75 1510 1513 1514 1515

1513 HC -0.220000 -2.320000 -19.630000 76 1512

1514 HC 0.740000 -2.070000 -20.900000 76 1512

1515 HC -0.200000 -0.680000 -20.240000 76 1512

1516 OH -2.470000 -2.440000 -20.780000 73 1510 1517

1517 HO -2.390000 -3.290000 -20.350000 74 1516

1518 C -2.500000 -1.680000 -23.490000 67 1508 1519 1520

1519 O -2.210000 -2.460000 -24.400000 69 1518

1520 N -3.760000 -1.230000 -23.330000 55 1518 1521 1522

1521 H -3.950000 -0.540000 -22.620000 58 1520

1522 CT -4.820000 -1.790000 -24.120000 56 1520 1523 1524 1529

1523 H1 -5.750000 -1.260000 -23.910000 60 1522

1524 CT -4.560000 -1.750000 -25.630000 61 1522 1525 1526 1527

1525 H1 -5.420000 -2.210000 -26.120000 62 1524

1526 H1 -3.700000 -2.310000 -25.990000 62 1524

1527 OH -4.510000 -0.410000 -26.090000 63 1524 1528

1528 HO -3.630000 -0.250000 -26.490000 64 1527

1529 C -4.980000 -3.220000 -23.700000 57 1522 1530 1531

1530 O -5.170000 -4.110000 -24.520000 59 1529

1531 N -4.910000 -3.440000 -22.370000 7 1529 1532 1533

1532 H -4.670000 -2.640000 -21.800000 10 1531

1533 CT -5.100000 -4.720000 -21.770000 8 1531 1534 1535 1539

1534 H1 -4.520000 -5.470000 -22.320000 12 1533

1535 CT -4.750000 -4.740000 -20.270000 13 1533 1536 1537 1538

1536 HC -5.410000 -5.380000 -19.690000 14 1535

1537 HC -3.690000 -4.890000 -20.100000 14 1535

1538 HC -4.980000 -3.750000 -19.880000 14 1535

1539 C -6.550000 -5.050000 -21.870000 9 1533 1540 1541

1540 O -7.400000 -4.170000 -22.020000 11 1539

1541 N -6.880000 -6.360000 -21.820000 210 1539 1542 1543

1542 H -6.180000 -7.080000 -21.680000 213 1541

1543 CT -8.250000 -6.770000 -21.910000 211 1541 1544 1545 1551

1544 H1 -8.750000 -6.150000 -22.660000 215 1543

1545 CT -8.430000 -8.250000 -22.320000 216 1543 1546 1547 1548

1546 HC -7.980000 -8.420000 -23.300000 217 1545

1547 HC -9.490000 -8.500000 -22.380000 217 1545

1548 C -7.730000 -9.110000 -21.280000 218 1545 1549 1550

1549 O2 -6.510000 -8.890000 -21.050000 219 1548

1550 O2 -8.400000 -10.020000 -20.710000 219 1548

1551 C -8.910000 -6.550000 -20.580000 212 1543 1552 1553

1552 O -8.250000 -6.300000 -19.580000 214 1551

1553 N -10.250000 -6.650000 -20.570000 244 1551 1554 1555

1554 H -10.700000 -6.830000 -21.460000 247 1553

1555 CT -11.040000 -6.410000 -19.390000 245 1553 1556 1557 1568

1556 H1 -10.820000 -5.410000 -19.040000 249 1555

1557 CT -12.550000 -6.490000 -19.650000 250 1555 1558 1559 1560

1558 HC -13.040000 -6.420000 -18.680000 251 1557

1559 HC -12.750000 -7.470000 -20.090000 251 1557

1560 CT -13.090000 -5.390000 -20.560000 252 1557 1561 1562 1563

1561 HC -12.620000 -5.440000 -21.540000 253 1560

1562 HC -12.900000 -4.410000 -20.120000 253 1560

1563 C -14.590000 -5.590000 -20.720000 254 1560 1564 1565

1564 O -15.260000 -4.820000 -21.400000 255 1563

1565 N -15.120000 -6.670000 -20.080000 256 1563 1566 1567

1566 H -16.100000 -6.780000 -20.240000 257 1565

1567 H -14.570000 -7.310000 -19.550000 257 1565

1568 C -10.710000 -7.440000 -18.360000 246 1555 1569 1570

1569 O -10.660000 -7.140000 -17.170000 248 1568

1570 N -10.460000 -8.690000 -18.790000 138 1568 1571 1572

1571 H -10.330000 -8.890000 -19.770000 141 1570

1572 CT -10.220000 -9.750000 -17.870000 139 1570 1573 1574 1592

1573 H1 -11.060000 -9.850000 -17.190000 143 1572

1574 CT -9.970000 -11.100000 -18.570000 144 1572 1575 1576 1577

1575 HC -9.820000 -11.800000 -17.750000 145 1574

1576 HC -9.030000 -11.250000 -19.060000 145 1574

1577 C* -11.170000 -11.630000 -19.310000 146 1574 1578 1591

1578 CW -11.540000 -11.430000 -20.610000 147 1577 1579 1580

1579 H4 -10.950000 -10.840000 -21.310000 148 1578

1580 NA -12.700000 -12.120000 -20.870000 150 1578 1581 1582

1581 H -13.150000 -12.160000 -21.770000 151 1580

1582 CN -13.090000 -12.780000 -19.720000 152 1580 1583 1591

1583 CA -14.160000 -13.590000 -19.470000 155 1582 1584 1585

1584 HA -14.880000 -13.810000 -20.240000 156 1583

1585 CA -14.280000 -14.110000 -18.200000 159 1583 1586 1587

1586 HA -15.120000 -14.760000 -17.970000 160 1585

1587 CA -13.350000 -13.830000 -17.220000 157 1585 1588 1589

1588 HA -13.470000 -14.260000 -16.240000 158 1587

1589 CA -12.270000 -13.020000 -17.470000 153 1587 1590 1591

1590 HA -11.540000 -12.800000 -16.700000 154 1589

1591 CB -12.150000 -12.500000 -18.730000 149 1577 1582 1589

1592 C -8.990000 -9.430000 -17.070000 140 1572 1593 1594

1593 O -8.990000 -9.560000 -15.840000 142 1592

1594 N -7.910000 -8.990000 -17.740000 65 1592 1595 1596

1595 H -7.910000 -9.030000 -18.760000 68 1594

1596 CT -6.690000 -8.720000 -17.050000 66 1594 1597 1598 1606

1597 H1 -6.390000 -9.600000 -16.480000 70 1596

1598 CT -5.550000 -8.360000 -17.960000 71 1596 1599 1600 1604

1599 H1 -5.910000 -7.470000 -18.470000 72 1598

1600 CT -4.320000 -8.070000 -17.090000 75 1598 1601 1602 1603

1601 HC -3.550000 -7.490000 -17.400000 76 1600

1602 HC -4.550000 -7.330000 -16.340000 76 1600

1603 HC -3.990000 -8.960000 -16.560000 76 1600

1604 OH -5.290000 -9.430000 -18.850000 73 1598 1605

1605 HO -5.700000 -9.200000 -19.730000 74 1604

1606 C -6.880000 -7.590000 -16.100000 67 1596 1607 1608

1607 O -6.380000 -7.620000 -14.980000 69 1606

1608 N -7.620000 -6.550000 -16.510000 41 1606 1609 1610

1609 H -7.970000 -6.540000 -17.470000 44 1608

1610 CT -7.770000 -5.420000 -15.640000 42 1608 1611 1612 1625

1611 H1 -6.770000 -5.100000 -15.330000 46 1610

1612 CT -8.470000 -4.250000 -16.280000 47 1610 1613 1614 1618

1613 HC -8.050000 -4.100000 -17.280000 48 1612

1614 CT -9.970000 -4.570000 -16.410000 51 1612 1615 1616 1617

1615 HC -10.480000 -3.660000 -16.730000 52 1614

1616 HC -10.020000 -5.110000 -17.310000 52 1614

1617 HC -10.560000 -4.860000 -15.550000 52 1614

1618 CT -8.180000 -2.980000 -15.470000 49 1612 1619 1620 1621

1619 HC -7.100000 -2.810000 -15.470000 50 1618

1620 HC -8.520000 -3.090000 -14.440000 50 1618

1621 CT -8.870000 -1.750000 -16.070000 53 1618 1622 1623 1624

1622 HC -8.270000 -0.970000 -15.640000 54 1621

1623 HC -8.730000 -1.720000 -17.150000 54 1621

1624 HC -9.920000 -1.710000 -15.790000 54 1621

1625 C -8.520000 -5.840000 -14.410000 43 1610 1626 1627

1626 O -8.180000 -5.430000 -13.310000 45 1625

1627 N -9.550000 -6.690000 -14.570000 1 1625 1628 1629

1628 H -9.780000 -6.990000 -15.510000 4 1627

1629 CT -10.340000 -7.090000 -13.440000 2 1627 1630 1631 1632

1630 H1 -11.130000 -7.770000 -13.770000 6 1629

1631 H1 -10.780000 -6.210000 -12.960000 6 1629

1632 C -9.470000 -7.800000 -12.450000 3 1629 1633 1634

1633 O -9.610000 -7.600000 -11.240000 5 1632

1634 N -8.560000 -8.670000 -12.920000 287 1632 1635 1636

1635 H -8.460000 -8.770000 -13.930000 290 1634

1636 CT -7.740000 -9.430000 -12.020000 288 1634 1637 1638 1656

1637 H1 -8.360000 -9.960000 -11.300000 292 1636

1638 CT -6.820000 -10.430000 -12.750000 293 1636 1639 1640 1641

1639 HC -6.240000 -10.890000 -11.950000 294 1638

1640 HC -6.150000 -9.900000 -13.440000 294 1638

1641 CT -7.590000 -11.520000 -13.510000 295 1638 1642 1643 1644

1642 HC -8.210000 -11.060000 -14.260000 296 1641

1643 HC -8.260000 -12.040000 -12.820000 296 1641

1644 CT -6.680000 -12.530000 -14.210000 297 1641 1645 1646 1647

1645 H1 -5.860000 -12.020000 -14.730000 298 1644

1646 H1 -7.270000 -13.070000 -14.960000 298 1644

1647 N2 -6.210000 -13.500000 -13.170000 299 1644 1648 1649

1648 H -6.740000 -14.330000 -12.980000 300 1647

1649 CA -5.060000 -13.270000 -12.490000 301 1647 1650 1653

1650 N2 -4.320000 -12.150000 -12.750000 302 1649 1651 1652

1651 H -3.700000 -11.770000 -12.040000 303 1650

1652 H -4.610000 -11.430000 -13.390000 303 1650

1653 N2 -4.630000 -14.160000 -11.540000 302 1649 1654 1655

1654 H -3.850000 -13.830000 -10.990000 303 1653

1655 H -5.160000 -14.960000 -11.280000 303 1653

1656 C -6.870000 -8.510000 -11.230000 289 1636 1657 1658

1657 O -6.730000 -8.670000 -10.020000 291 1656

1658 N -6.260000 -7.520000 -11.900000 65 1656 1659 1660

1659 H -6.270000 -7.530000 -12.920000 68 1658

1660 CT -5.340000 -6.640000 -11.230000 66 1658 1661 1662 1670

1661 H1 -4.560000 -7.230000 -10.750000 70 1660

1662 CT -4.700000 -5.650000 -12.160000 71 1660 1663 1664 1668

1663 H1 -5.460000 -5.020000 -12.620000 72 1662

1664 CT -3.730000 -4.780000 -11.350000 75 1662 1665 1666 1667

1665 HC -3.180000 -4.130000 -12.030000 76 1664

1666 HC -4.250000 -4.140000 -10.640000 76 1664

1667 HC -3.010000 -5.410000 -10.820000 76 1664

1668 OH -3.990000 -6.340000 -13.190000 73 1662 1669

1669 HO -3.460000 -5.730000 -13.710000 74 1668

1670 C -6.070000 -5.860000 -10.180000 67 1660 1671 1672

1671 O -5.580000 -5.690000 -9.070000 69 1670

1672 N -7.260000 -5.350000 -10.520000 41 1670 1673 1674

1673 H -7.600000 -5.520000 -11.460000 44 1672

1674 CT -8.000000 -4.550000 -9.590000 42 1672 1675 1676 1689

1675 H1 -7.360000 -3.740000 -9.250000 46 1674

1676 CT -9.250000 -3.970000 -10.180000 47 1674 1677 1678 1682

1677 HC -9.850000 -4.780000 -10.590000 48 1676

1678 CT -10.050000 -3.310000 -9.050000 51 1676 1679 1680 1681

1679 HC -10.940000 -2.820000 -9.450000 52 1678

1680 HC -10.430000 -4.040000 -8.340000 52 1678

1681 HC -9.430000 -2.570000 -8.540000 52 1678

1682 CT -8.900000 -3.010000 -11.330000 49 1676 1683 1684 1685

1683 HC -8.300000 -3.480000 -12.090000 50 1682

1684 HC -8.290000 -2.200000 -10.930000 50 1682

1685 CT -10.140000 -2.390000 -11.970000 53 1682 1686 1687 1688

1686 HC -9.830000 -1.770000 -12.810000 54 1685

1687 HC -10.800000 -3.180000 -12.340000 54 1685

1688 HC -10.670000 -1.750000 -11.270000 54 1685

1689 C -8.370000 -5.370000 -8.400000 43 1674 1690 1691

1690 O -8.280000 -4.900000 -7.270000 45 1689

1691 N -8.790000 -6.630000 -8.620000 27 1689 1692 1693

1692 H -8.810000 -7.000000 -9.570000 30 1691

1693 CT -9.220000 -7.430000 -7.510000 28 1691 1694 1695 1708

1694 H1 -10.000000 -6.890000 -6.980000 32 1693

1695 CT -9.730000 -8.820000 -7.950000 33 1693 1696 1697 1698

1696 HC -8.930000 -9.340000 -8.490000 34 1695

1697 HC -10.560000 -8.690000 -8.640000 34 1695

1698 CT -10.180000 -9.690000 -6.770000 35 1695 1699 1700 1704

1699 HC -9.370000 -9.770000 -6.040000 36 1698

1700 CT -11.390000 -9.060000 -6.040000 37 1698 1701 1702 1703

1701 HC -11.710000 -9.730000 -5.240000 38 1700

1702 HC -11.110000 -8.110000 -5.600000 38 1700

1703 HC -12.210000 -8.930000 -6.740000 38 1700

1704 CT -10.450000 -11.130000 -7.220000 39 1698 1705 1706 1707

1705 HC -10.750000 -11.730000 -6.360000 40 1704

1706 HC -11.240000 -11.140000 -7.970000 40 1704

1707 HC -9.540000 -11.550000 -7.650000 40 1704

1708 C -8.080000 -7.660000 -6.570000 29 1693 1709 1710

1709 O -8.240000 -7.530000 -5.350000 31 1708

1710 N -6.880000 -7.980000 -7.100000 232 1708 1711 1712

1711 H -6.760000 -8.070000 -8.110000 235 1710

1712 CT -5.770000 -8.250000 -6.240000 233 1710 1713 1714 1723

1713 H1 -6.000000 -9.060000 -5.540000 237 1712

1714 CT -4.510000 -8.600000 -7.030000 238 1712 1715 1716 1717

1715 HC -3.630000 -8.640000 -6.410000 239 1714

1716 HC -4.370000 -7.790000 -7.740000 239 1714

1717 CT -4.630000 -9.910000 -7.830000 240 1714 1718 1719 1720

1718 HC -5.560000 -9.960000 -8.390000 241 1717

1719 HC -4.630000 -10.750000 -7.130000 241 1717

1720 C -3.420000 -10.020000 -8.750000 242 1717 1721 1722

1721 O2 -2.560000 -9.100000 -8.730000 243 1720

1722 O2 -3.330000 -11.040000 -9.490000 243 1720

1723 C -5.480000 -7.010000 -5.470000 234 1712 1724 1725

1724 O -5.300000 -7.030000 -4.250000 236 1723

1725 N -5.460000 -5.860000 -6.160000 55 1723 1726 1727

1726 H -5.570000 -5.890000 -7.170000 58 1725

1727 CT -5.080000 -4.630000 -5.530000 56 1725 1728 1729 1734

1728 H1 -4.090000 -4.750000 -5.110000 60 1727

1729 CT -5.050000 -3.440000 -6.510000 61 1727 1730 1731 1732

1730 H1 -4.300000 -3.550000 -7.300000 62 1729

1731 H1 -4.910000 -2.600000 -5.850000 62 1729

1732 OH -6.350000 -3.150000 -7.010000 63 1729 1733

1733 HO -6.180000 -2.470000 -7.670000 64 1732

1734 C -6.040000 -4.290000 -4.440000 57 1727 1735 1736

1735 O -5.630000 -3.810000 -3.380000 59 1734

1736 N -7.340000 -4.540000 -4.660000 161 1734 1737 1738

1737 H -7.560000 -4.850000 -5.610000 164 1736

1738 CT -8.320000 -4.200000 -3.680000 162 1736 1739 1740 1752

1739 H1 -8.210000 -3.150000 -3.420000 166 1738

1740 CT -9.750000 -4.500000 -4.160000 167 1738 1741 1742 1743

1741 HC -9.930000 -5.580000 -4.170000 168 1740

1742 HC -9.830000 -4.170000 -5.200000 168 1740

1743 CC -10.820000 -3.810000 -3.370000 169 1740 1744 1750

1744 NA -11.240000 -2.520000 -3.620000 170 1743 1745 1746

1745 H -10.860000 -1.930000 -4.360000 171 1744

1746 CR -12.210000 -2.250000 -2.710000 174 1744 1747 1748

1747 H5 -12.750000 -1.310000 -2.680000 175 1746

1748 NA -12.430000 -3.260000 -1.890000 176 1746 1749 1750

1749 H -13.130000 -3.350000 -1.160000 177 1748

1750 CW -11.550000 -4.240000 -2.310000 172 1743 1748 1751

1751 H4 -11.490000 -5.280000 -2.080000 173 1750

1752 C -8.060000 -5.020000 -2.460000 163 1738 1753 1754

1753 O -8.150000 -4.540000 -1.330000 165 1752

1754 N -7.700000 -6.310000 -2.660000 1 1752 1755 1756

1755 H -7.560000 -6.650000 -3.610000 4 1754

1756 CT -7.460000 -7.190000 -1.560000 2 1754 1757 1758 1759

1757 H1 -7.230000 -8.190000 -1.930000 6 1756

1758 H1 -8.340000 -7.250000 -0.920000 6 1756

1759 C -6.310000 -6.670000 -0.760000 3 1756 1760 1761

1760 O -6.350000 -6.690000 0.470000 5 1759

1761 N -5.250000 -6.170000 -1.430000 27 1759 1762 1763

1762 H -5.220000 -6.230000 -2.450000 30 1761

1763 CT -4.100000 -5.750000 -0.690000 28 1761 1764 1765 1778

1764 H1 -3.760000 -6.580000 -0.070000 32 1763

1765 CT -2.909884 -5.448024 -1.643706 33 1763 1766 1767 1768

1766 HC -3.093931 -5.966330 -2.577516 34 1765

1767 HC -2.015382 -5.900350 -1.215915 34 1765

1768 CT -2.558274 -3.999671 -2.023922 35 1765 1769 1770 1774

1769 HC -3.460155 -3.442960 -2.235518 36 1768

1770 CT -1.768089 -3.285726 -0.929870 37 1768 1771 1772 1773

1771 HC -2.316964 -3.259537 0.006098 38 1770

1772 HC -0.818400 -3.794532 -0.771543 38 1770

1773 HC -1.597951 -2.263126 -1.255105 38 1770

1774 CT -1.676761 -3.969104 -3.274349 39 1768 1775 1776 1777

1775 HC -0.734581 -4.478070 -3.089834 40 1774

1776 HC -2.181226 -4.470917 -4.098193 40 1774

1777 HC -1.490266 -2.938049 -3.578612 40 1774

1778 C -4.480000 -4.640000 0.230000 29 1763 1779 1780

1779 O -4.080000 -4.620000 1.400000 31 1778

1780 N -5.290000 -3.670000 -0.250000 27 1778 1781 1782

1781 H -5.540000 -3.720000 -1.230000 30 1780

1782 CT -5.680000 -2.560000 0.570000 28 1780 1783 1784 1797

1783 H1 -4.810000 -2.130000 1.060000 32 1782

1784 CT -6.288487 -1.493625 -0.328407 33 1782 1785 1786 1787

1785 HC -6.649966 -0.669746 0.288666 34 1784

1786 HC -7.130140 -1.920244 -0.877119 34 1784

1787 CT -5.226930 -0.976938 -1.319329 35 1784 1788 1789 1793

1788 HC -4.824522 -1.777381 -1.928471 36 1787

1789 CT -5.934046 -0.024224 -2.243308 37 1787 1790 1791 1792

1790 HC -5.201646 0.454309 -2.882522 38 1789

1791 HC -6.643883 -0.565830 -2.868679 38 1789

1792 HC -6.438247 0.710097 -1.634087 38 1789

1793 CT -4.070195 -0.240955 -0.633446 39 1787 1794 1795 1796

1794 HC -3.449215 0.248886 -1.384620 40 1793

1795 HC -4.460410 0.492673 0.067083 40 1793

1796 HC -3.439984 -0.950844 -0.101670 40 1793

1797 C -6.570000 -3.030000 1.680000 29 1782 1798 1799

1798 O -6.420000 -2.590000 2.820000 31 1797

1799 N -7.510000 -3.950000 1.380000 27 1797 1800 1801

1800 H -7.540000 -4.300000 0.430000 30 1799

1801 CT -8.470000 -4.400000 2.360000 28 1799 1802 1803 1816

1802 H1 -9.000000 -3.550000 2.780000 32 1801

1803 CT -9.460000 -5.430000 1.780000 33 1801 1804 1805 1806

1804 HC -10.110000 -5.770000 2.590000 34 1803

1805 HC -8.910000 -6.300000 1.410000 34 1803

1806 CT -10.330000 -4.890000 0.630000 35 1803 1807 1808 1812

1807 HC -9.860000 -4.550000 -0.210000 36 1806

1808 CT -11.290000 -5.970000 0.120000 37 1806 1809 1810 1811

1809 HC -12.230000 -5.560000 -0.230000 38 1808

1810 HC -10.790000 -6.630000 -0.590000 38 1808

1811 HC -11.580000 -6.600000 0.970000 38 1808

1812 CT -11.040000 -3.590000 1.020000 39 1806 1813 1814 1815

1813 HC -11.750000 -3.240000 0.310000 40 1812

1814 HC -11.580000 -3.760000 1.950000 40 1812

1815 HC -10.310000 -2.800000 1.180000 40 1812

1816 C -7.760000 -5.080000 3.480000 29 1801 1817 1818

1817 O -8.040000 -4.820000 4.650000 31 1816

1818 N -6.810000 -5.970000 3.150000 107 1816 1819 1820

1819 H -6.600000 -6.170000 2.180000 110 1818

1820 CT -6.130000 -6.700000 4.190000 108 1818 1821 1822 1836

1821 H1 -6.860000 -7.170000 4.850000 112 1820

1822 CT -5.130000 -7.770000 3.690000 113 1820 1823 1824 1825

1823 HC -4.460000 -8.140000 4.470000 114 1822

1824 HC -4.640000 -7.310000 2.860000 114 1822

1825 CA -5.850000 -8.880000 3.010000 115 1822 1826 1834

1826 CA -6.650000 -9.750000 3.720000 116 1825 1827 1828

1827 HA -6.810000 -9.610000 4.770000 117 1826

1828 CA -7.300000 -10.780000 3.080000 118 1826 1829 1830

1829 HA -7.940000 -11.440000 3.640000 119 1828

1830 CA -7.160000 -10.960000 1.720000 120 1828 1831 1832

1831 HA -7.730000 -11.730000 1.210000 121 1830

1832 CA -6.350000 -10.100000 1.010000 118 1830 1833 1834

1833 HA -6.600000 -10.110000 -0.020000 119 1832

1834 CA -5.700000 -9.080000 1.650000 116 1825 1832 1835

1835 HA -5.030000 -8.420000 1.140000 117 1834

1836 C -5.350000 -5.720000 5.010000 109 1820 1837 1838

1837 O -5.270000 -5.850000 6.230000 111 1836

1838 N -4.679184 -4.768367 4.352420 1 1836 1839 1840

1839 H -4.671356 -4.807549 3.337547 4 1838

1840 CT -3.897030 -3.742813 5.016520 2 1838 1841 1842 1843

1841 H1 -3.517889 -3.042018 4.275063 6 1840

1842 H1 -3.057966 -4.205615 5.536975 6 1840

1843 C -4.750000 -2.980000 6.020000 3 1840 1844 1845

1844 O -4.280000 -2.700000 7.120000 5 1843

1845 N -5.990000 -2.610000 5.640000 107 1843 1846 1847

1846 H -6.290000 -2.830000 4.700000 110 1845

1847 CT -6.840000 -1.850000 6.510000 108 1845 1848 1849 1863

1848 H1 -6.340000 -0.940000 6.820000 112 1847

1849 CT -8.131381 -1.527614 5.711857 113 1847 1850 1851 1852

1850 HC -8.669884 -2.456113 5.523431 114 1849

1851 HC -7.814898 -1.154692 4.736432 114 1849

1852 CA -9.145615 -0.527135 6.264365 115 1849 1853 1861

1853 CA -9.707756 0.423337 5.387152 116 1852 1854 1855

1854 HA -9.424057 0.425740 4.345124 117 1853

1855 CA -10.638660 1.372358 5.848480 118 1853 1856 1857

1856 HA -11.051018 2.104243 5.168603 119 1855

1857 CA -11.043718 1.360882 7.193101 120 1855 1858 1859

1858 HA -11.767224 2.080174 7.552100 121 1857

1859 CA -10.537207 0.381279 8.062538 118 1857 1860 1861

1860 HA -10.880074 0.339102 9.087770 119 1859

1861 CA -9.607306 -0.566012 7.597062 116 1852 1859 1862

1862 HA -9.285968 -1.330930 8.282054 117 1861

1863 C -7.140000 -2.660000 7.730000 109 1847 1864 1865

1864 O -7.080000 -2.150000 8.850000 111 1863

1865 N -7.440000 -3.950000 7.540000 27 1863 1866 1867

1866 H -7.450000 -4.310000 6.590000 30 1865

1867 CT -7.790000 -4.800000 8.640000 28 1865 1868 1869 1882

1868 H1 -8.650000 -4.380000 9.140000 32 1867

1869 CT -8.190000 -6.220000 8.160000 33 1867 1870 1871 1872

1870 HC -7.310000 -6.710000 7.750000 34 1869

1871 HC -8.910000 -6.110000 7.350000 34 1869

1872 CT -8.820000 -7.150000 9.210000 35 1869 1873 1874 1878

1873 HC -9.760000 -6.710000 9.530000 36 1872

1874 CT -9.170000 -8.500000 8.580000 37 1872 1875 1876 1877

1875 HC -9.300000 -9.280000 9.320000 38 1874

1876 HC -10.130000 -8.390000 8.070000 38 1874

1877 HC -8.470000 -8.690000 7.790000 38 1874

1878 CT -7.970000 -7.320000 10.480000 39 1872 1879 1880 1881

1879 HC -8.240000 -8.250000 10.970000 40 1878

1880 HC -6.910000 -7.370000 10.250000 40 1878

1881 HC -8.230000 -6.560000 11.200000 40 1878

1882 C -6.600000 -4.900000 9.530000 29 1867 1883 1884

1883 O -6.720000 -4.890000 10.760000 31 1882

1884 N -5.390000 -4.980000 8.950000 7 1882 1885 1886

1885 H -5.320000 -5.070000 7.940000 10 1884

1886 CT -4.220000 -5.100000 9.760000 8 1884 1887 1888 1892

1887 H1 -4.300000 -5.990000 10.390000 12 1886

1888 CT -2.975602 -5.207827 8.859007 13 1886 1889 1890 1891

1889 HC -2.119583 -5.509801 9.462520 14 1888

1890 HC -3.126049 -5.929902 8.061468 14 1888

1891 HC -2.745180 -4.247883 8.401867 14 1888

1892 C -4.100000 -3.890000 10.630000 9 1886 1893 1894

1893 O -3.790000 -4.000000 11.810000 11 1892

1894 N -4.360000 -2.700000 10.060000 55 1892 1895 1896

1895 H -4.560000 -2.670000 9.070000 58 1894

1896 CT -4.220000 -1.500000 10.840000 56 1894 1897 1898 1903

1897 H1 -3.210000 -1.460000 11.250000 60 1896

1898 CT -4.500000 -0.220000 10.040000 61 1896 1899 1900 1901

1899 H1 -4.580000 0.620000 10.730000 62 1898

1900 H1 -5.430000 -0.320000 9.490000 62 1898

1901 OH -3.430000 0.030000 9.150000 63 1898 1902

1902 HO -3.620000 0.770000 8.580000 64 1901

1903 C -5.200000 -1.540000 11.960000 57 1896 1904 1905

1904 O -4.860000 -1.180000 13.080000 59 1903

1905 N -6.450000 -1.960000 11.720000 41 1903 1906 1907

1906 H -6.720000 -2.290000 10.800000 44 1905

1907 CT -7.370000 -1.940000 12.810000 42 1905 1908 1909 1922

1908 H1 -7.360000 -0.960000 13.290000 46 1907

1909 CT -8.790000 -2.220000 12.390000 47 1907 1910 1911 1915

1910 HC -9.420000 -2.190000 13.280000 48 1909

1911 CT -9.220000 -1.060000 11.470000 51 1909 1912 1913 1914

1912 HC -10.280000 -1.140000 11.250000 52 1911

1913 HC -9.070000 -0.110000 11.980000 52 1911

1914 HC -8.660000 -1.060000 10.540000 52 1911

1915 CT -8.930000 -3.610000 11.750000 49 1909 1916 1917 1918

1916 HC -8.610000 -4.430000 12.390000 50 1915

1917 HC -8.400000 -3.480000 10.840000 50 1915

1918 CT -10.370000 -3.890000 11.310000 53 1915 1919 1920 1921

1919 HC -10.430000 -4.900000 10.920000 54 1918

1920 HC -11.040000 -3.810000 12.160000 54 1918

1921 HC -10.680000 -3.200000 10.520000 54 1918

1922 C -6.930000 -2.930000 13.860000 43 1907 1923 1924

1923 O -6.990000 -2.650000 15.060000 45 1922

1924 N -6.440000 -4.110000 13.430000 7 1922 1925 1926

1925 H -6.350000 -4.250000 12.430000 10 1924

1926 CT -6.090000 -5.200000 14.300000 8 1924 1927 1928 1932

1927 H1 -6.910000 -5.340000 15.010000 12 1926

1928 CT -5.840000 -6.520000 13.570000 13 1926 1929 1930 1931

1929 HC -5.580000 -7.320000 14.260000 14 1928

1930 HC -6.740000 -6.810000 13.030000 14 1928

1931 HC -5.040000 -6.390000 12.840000 14 1928

1932 C -4.850000 -4.890000 15.080000 9 1926 1933 1934

1933 O -4.150000 -3.910000 14.830000 11 1932

1934 N -4.590000 -5.720000 16.110000 287 1932 1935 1936

1935 H -5.130000 -6.580000 16.150000 290 1934

1936 CT -3.410000 -5.580000 16.910000 288 1934 1937 1938 1956

1937 H1 -2.640000 -5.070000 16.330000 292 1936

1938 CT -3.650000 -4.830000 18.230000 293 1936 1939 1940 1941

1939 HC -4.410000 -5.360000 18.800000 294 1938

1940 HC -4.040000 -3.850000 17.950000 294 1938

1941 CT -2.390000 -4.640000 19.070000 295 1938 1942 1943 1944

1942 HC -1.540000 -4.430000 18.420000 296 1941

1943 HC -2.190000 -5.550000 19.640000 296 1941

1944 CT -2.510000 -3.500000 20.090000 297 1941 1945 1946 1947

1945 H1 -2.240000 -2.570000 19.590000 298 1944

1946 H1 -1.830000 -3.640000 20.930000 298 1944

1947 N2 -3.940000 -3.420000 20.490000 299 1944 1948 1949

1948 H -4.590000 -2.900000 19.920000 300 1947

1949 CA -4.410000 -4.210000 21.510000 301 1947 1950 1953

1950 N2 -3.560000 -5.050000 22.170000 302 1949 1951 1952

1951 H -3.850000 -5.320000 23.090000 303 1950

1952 H -2.580000 -4.990000 21.940000 303 1950

1953 N2 -5.730000 -4.140000 21.850000 302 1949 1954 1955

1954 H -6.120000 -4.640000 22.630000 303 1953

1955 H -6.350000 -3.530000 21.340000 303 1953

1956 C -2.930000 -6.970000 17.190000 289 1936 1957 1958

1957 O -3.610000 -7.940000 16.880000 291 1956

1958 N -1.700000 -7.090000 17.740000 161 1956 1959 1960

1959 H -1.070000 -6.380000 17.370000 164 1958

1960 CT -1.170000 -8.390000 18.050000 162 1958 1961 1962 1974

1961 H1 -0.300000 -8.250000 18.690000 166 1960

1962 CT -2.170000 -9.330000 18.750000 167 1960 1963 1964 1965

1963 HC -1.950000 -10.380000 18.750000 168 1962

1964 HC -3.170000 -9.320000 18.430000 168 1962

1965 CC -2.460000 -8.930000 20.170000 169 1962 1966 1972

1966 NA -3.510000 -8.120000 20.550000 170 1965 1967 1968

1967 H -4.240000 -7.820000 19.910000 171 1966

1968 CR -3.440000 -7.990000 21.890000 174 1966 1969 1970

1969 H5 -4.230000 -7.540000 22.490000 175 1968

1970 NA -2.430000 -8.670000 22.410000 176 1968 1971 1972

1971 H -2.200000 -8.830000 23.390000 177 1970

1972 CW -1.810000 -9.260000 21.320000 172 1965 1970 1973

1973 H4 -0.940000 -9.910000 21.360000 173 1972

1974 C -0.660000 -9.010000 16.790000 163 1960 1975 1976

1975 O -0.560000 -8.340000 15.760000 165 1974

1976 N -0.310000 -10.260000 16.830000 96 1974 1977 1986

1977 CT 0.360000 -10.800000 18.010000 105 1976 1978 1979 1980

1978 H1 -0.300000 -11.310000 18.700000 106 1977

1979 H1 0.950000 -10.050000 18.530000 106 1977

1980 CT 1.290000 -11.910000 17.500000 103 1977 1981 1982 1983

1981 HC 1.270000 -12.790000 18.140000 104 1980

1982 HC 2.310000 -11.520000 17.420000 104 1980

1983 CT 0.760000 -12.230000 16.090000 101 1980 1984 1985 1986

1984 HC -0.020000 -12.990000 16.160000 102 1983

1985 HC 1.560000 -12.560000 15.430000 102 1983

1986 CT 0.150000 -10.910000 15.640000 97 1976 1983 1987 1988

1987 H1 0.950000 -10.290000 15.230000 100 1986

1988 C -0.930000 -11.030000 14.620000 98 1986 1989 1990

1989 O -0.640000 -11.370000 13.480000 99 1988

1990 N -2.190000 -10.770000 15.000000 122 1988 1991 1992

1991 H -2.390000 -10.520000 15.950000 125 1990

1992 CT -3.240000 -10.860000 14.040000 123 1990 1993 1994 2009

1993 H1 -3.260000 -11.860000 13.590000 127 1992

1994 CT -4.620000 -10.520000 14.640000 128 1992 1995 1996 1997

1995 HC -5.390000 -10.610000 13.870000 129 1994

1996 HC -4.630000 -9.500000 15.020000 129 1994

1997 CA -4.870000 -11.480000 15.740000 130 1994 1998 2007

1998 CA -4.320000 -11.280000 16.980000 131 1997 1999 2000

1999 HA -3.720000 -10.420000 17.020000 132 1998

2000 CA -4.540000 -12.160000 18.010000 133 1998 2001 2002

2001 HA -4.090000 -12.000000 18.980000 134 2000

2002 C -5.330000 -13.270000 17.810000 135 2000 2003 2005

2003 OH -5.560000 -14.170000 18.860000 136 2002 2004

2004 HO -5.040000 -13.990000 19.640000 137 2003

2005 CA -5.890000 -13.480000 16.570000 133 2002 2006 2007

2006 HA -6.500000 -14.360000 16.420000 134 2005

2007 CA -5.660000 -12.600000 15.540000 131 1997 2005 2008

2008 HA -6.100000 -12.790000 14.570000 132 2007

2009 C -2.940000 -9.860000 12.970000 124 1992 2010 2011

2010 O -3.070000 -10.140000 11.780000 126 2009

2011 N -2.500000 -8.650000 13.370000 210 2009 2012 2013

2012 H -2.220000 -8.490000 14.330000 213 2011

2013 CT -2.230000 -7.630000 12.410000 211 2011 2014 2015 2021

2014 H1 -3.100000 -7.530000 11.760000 215 2013

2015 CT -1.960000 -6.230000 13.020000 216 2013 2016 2017 2018

2016 HC -2.830000 -5.920000 13.600000 217 2015

2017 HC -1.800000 -5.520000 12.210000 217 2015

2018 C -0.720000 -6.230000 13.910000 218 2015 2019 2020

2019 O2 0.390000 -6.550000 13.410000 219 2018

2020 O2 -0.880000 -5.900000 15.120000 219 2018

2021 C -1.090000 -8.030000 11.520000 212 2013 2022 2023

2022 O -1.100000 -7.720000 10.330000 214 2021

2023 N -0.070000 -8.730000 12.060000 232 2021 2024 2025

2024 H 0.030000 -8.730000 13.060000 235 2023

2025 CT 1.060000 -9.070000 11.240000 233 2023 2026 2027 2036

2026 H1 1.450000 -8.160000 10.780000 237 2025

2027 CT 2.210000 -9.760000 11.990000 238 2025 2028 2029 2030

2028 HC 2.500000 -9.140000 12.840000 239 2027

2029 HC 3.040000 -9.800000 11.280000 239 2027

2030 CT 1.890000 -11.180000 12.460000 240 2027 2031 2032 2033

2031 HC 1.440000 -11.880000 11.760000 241 2030

2032 HC 1.430000 -11.020000 13.390000 241 2030

2033 C 3.170000 -11.790000 13.030000 242 2030 2034 2035

2034 O2 4.210000 -11.080000 13.010000 243 2033

2035 O2 3.110000 -12.960000 13.480000 243 2033

2036 C 0.620000 -10.010000 10.160000 234 2025 2037 2038

2037 O 1.090000 -9.940000 9.030000 236 2036

2038 N -0.310000 -10.930000 10.490000 27 2036 2039 2040

2039 H -0.610000 -10.970000 11.460000 30 2038

2040 CT -0.730000 -11.910000 9.540000 28 2038 2041 2042 2055

2041 H1 0.160000 -12.420000 9.180000 32 2040

2042 CT -1.720000 -12.930000 10.140000 33 2040 2043 2044 2045

2043 HC -2.640000 -12.420000 10.440000 34 2042

2044 HC -1.270000 -13.340000 11.050000 34 2042

2045 CT -2.070000 -14.110000 9.210000 35 2042 2046 2047 2051

2046 HC -2.670000 -14.810000 9.800000 36 2045

2047 CT -2.940000 -13.680000 8.020000 37 2045 2048 2049 2050

2048 HC -3.500000 -14.570000 7.730000 38 2047

2049 HC -3.630000 -12.870000 8.260000 38 2047

2050 HC -2.270000 -13.510000 7.220000 38 2047

2051 CT -0.790000 -14.850000 8.770000 39 2045 2052 2053 2054

2052 HC -1.070000 -15.740000 8.210000 40 2051

2053 HC -0.180000 -14.220000 8.120000 40 2051

2054 HC -0.210000 -15.150000 9.650000 40 2051

2055 C -1.380000 -11.210000 8.380000 29 2040 2056 2057

2056 O -1.120000 -11.560000 7.230000 31 2055

2057 N -2.230000 -10.200000 8.650000 107 2055 2058 2059

2058 H -2.370000 -9.950000 9.620000 110 2057

2059 CT -2.900000 -9.490000 7.590000 108 2057 2060 2061 2075

2060 H1 -3.380000 -10.220000 6.940000 112 2059

2061 CT -3.970000 -8.500000 8.080000 113 2059 2062 2063 2064

2062 HC -4.270000 -7.840000 7.270000 114 2061

2063 HC -3.580000 -7.930000 8.920000 114 2061

2064 CA -5.140000 -9.310000 8.520000 115 2061 2065 2073

2065 CA -6.040000 -9.790000 7.590000 116 2064 2066 2067

2066 HA -5.850000 -9.590000 6.550000 117 2065

2067 CA -7.120000 -10.540000 7.980000 118 2065 2068 2069

2068 HA -7.800000 -10.940000 7.240000 119 2067

2069 CA -7.330000 -10.810000 9.310000 120 2067 2070 2071

2070 HA -8.180000 -11.410000 9.620000 121 2069

2071 CA -6.450000 -10.330000 10.250000 118 2069 2072 2073

2072 HA -6.610000 -10.530000 11.300000 119 2071

2073 CA -5.360000 -9.590000 9.850000 116 2064 2071 2074

2074 HA -4.720000 -9.140000 10.580000 117 2073

2075 C -1.910000 -8.770000 6.750000 109 2059 2076 2077

2076 O -2.050000 -8.720000 5.520000 111 2075

2077 N -0.880000 -8.180000 7.370000 7 2075 2078 2079

2078 H -0.790000 -8.240000 8.380000 10 2077

2079 CT 0.070000 -7.470000 6.590000 8 2077 2080 2081 2085

2080 H1 -0.440000 -6.700000 6.010000 12 2079

2081 CT 1.083907 -6.788946 7.518091 13 2079 2082 2083 2084

2082 HC 1.793328 -6.211105 6.925698 14 2081

2083 HC 0.565257 -6.123590 8.209848 14 2081

2084 HC 1.631761 -7.536526 8.094039 14 2081

2085 C 0.710000 -8.430000 5.630000 9 2079 2086 2087

2086 O 0.930000 -8.090000 4.470000 11 2085

2087 N 1.010000 -9.660000 6.090000 65 2085 2088 2089

2088 H 0.900000 -9.860000 7.080000 68 2087

2089 CT 1.660000 -10.630000 5.260000 66 2087 2090 2091 2099

2090 H1 2.600000 -10.220000 4.900000 70 2089

2091 CT 1.940000 -11.920000 5.980000 71 2089 2092 2093 2097

2092 H1 1.010000 -12.380000 6.310000 72 2091

2093 CT 2.650000 -12.880000 5.010000 75 2091 2094 2095 2096

2094 HC 2.930000 -13.780000 5.560000 76 2093

2095 HC 2.000000 -13.190000 4.190000 76 2093

2096 HC 3.550000 -12.410000 4.620000 76 2093

2097 OH 2.760000 -11.680000 7.110000 73 2091 2098

2098 HO 2.740000 -12.440000 7.690000 74 2097

2099 C 0.790000 -10.950000 4.090000 67 2089 2100 2101

2100 O 1.250000 -11.040000 2.960000 69 2099

2101 N -0.530000 -11.130000 4.330000 41 2099 2102 2103

2102 H -0.850000 -11.060000 5.290000 44 2101

2103 CT -1.440000 -11.490000 3.280000 42 2101 2104 2105 2118

2104 H1 -1.110000 -12.400000 2.790000 46 2103

2105 CT -2.850000 -11.650000 3.770000 47 2103 2106 2107 2111

2106 HC -3.180000 -10.720000 4.240000 48 2105

2107 CT -3.740000 -11.900000 2.540000 51 2105 2108 2109 2110

2108 HC -4.780000 -12.040000 2.830000 52 2107

2109 HC -3.770000 -11.050000 1.860000 52 2107

2110 HC -3.400000 -12.780000 2.000000 52 2107

2111 CT -2.940000 -12.770000 4.810000 49 2105 2112 2113 2114

2112 HC -2.280000 -12.490000 5.580000 50 2111

2113 HC -2.600000 -13.710000 4.370000 50 2111

2114 CT -4.370000 -12.960000 5.330000 53 2111 2115 2116 2117

2115 HC -4.430000 -13.820000 5.980000 54 2114

2116 HC -4.700000 -12.060000 5.850000 54 2114

2117 HC -5.060000 -13.190000 4.520000 54 2114

2118 C -1.450000 -10.380000 2.280000 43 2103 2119 2120

2119 O -1.430000 -10.620000 1.070000 45 2118

2120 N -1.470000 -9.130000 2.750000 55 2118 2121 2122

2121 H -1.550000 -8.960000 3.750000 58 2120

2122 CT -1.530000 -8.030000 1.830000 56 2120 2123 2124 2129

2123 H1 -2.410000 -8.110000 1.200000 60 2122

2124 CT -1.517770 -6.697177 2.588296 61 2122 2125 2126 2127

2125 H1 -1.770653 -5.888241 1.901696 62 2124

2126 H1 -0.509036 -6.539211 2.946301 62 2124

2127 OH -2.352999 -6.633103 3.723886 63 2124 2128

2128 HO -2.181157 -7.388098 4.312627 64 2127

2129 C -0.310000 -8.040000 0.970000 57 2122 2130 2131

2130 O -0.380000 -7.780000 -0.230000 59 2129

2131 N 0.860000 -8.360000 1.560000 258 2129 2132 2133

2132 H 0.860000 -8.590000 2.550000 261 2131

2133 CT 2.090000 -8.300000 0.830000 259 2131 2134 2135 2146

2134 H1 2.150000 -7.320000 0.360000 263 2133

2135 CT 3.309413 -8.361443 1.781122 264 2133 2136 2137 2138

2136 HC 2.998740 -8.044403 2.777074 265 2135

2137 HC 4.037432 -7.624379 1.439675 265 2135

2138 CT 4.050101 -9.698887 1.915473 266 2135 2139 2140 2141

2139 H1 3.334748 -10.513068 1.976799 267 2138

2140 H1 4.610816 -9.682215 2.850484 267 2138

2141 S 5.222515 -10.057020 0.580431 268 2138 2142

2142 CT 5.679644 -11.748114 1.041756 269 2141 2143 2144 2145

2143 H1 6.406853 -12.136129 0.328441 270 2142

2144 H1 6.117198 -11.751086 2.040305 270 2142

2145 H1 4.792669 -12.382058 1.032044 270 2142

2146 C 2.100000 -9.310000 -0.280000 260 2133 2147 2148

2147 O 2.640000 -9.030000 -1.350000 262 2146

2148 N 1.530000 -10.510000 -0.060000 77 2146 2149 2150

2149 H 1.100000 -10.710000 0.830000 80 2148

2150 CT 1.560000 -11.480000 -1.110000 78 2148 2151 2152 2157

2151 H1 2.590000 -11.620000 -1.440000 82 2150

2152 CT 0.970000 -12.860000 -0.720000 83 2150 2153 2154 2155

2153 H1 1.410000 -13.170000 0.230000 84 2152

2154 H1 1.260000 -13.580000 -1.490000 84 2152

2155 SH -0.840000 -12.880000 -0.580000 85 2152 2156

2156 HS -0.970000 -14.170000 -0.250000 86 2155

2157 C 0.780000 -10.950000 -2.270000 79 2150 2158 2159

2158 O 1.180000 -11.110000 -3.420000 81 2157

2159 N -0.350000 -10.270000 -1.990000 41 2157 2160 2161

2160 H -0.660000 -10.240000 -1.020000 44 2159

2161 CT -1.170000 -9.750000 -3.040000 42 2159 2162 2163 2176

2162 H1 -1.430000 -10.560000 -3.730000 46 2161

2163 CT -2.420000 -9.080000 -2.580000 47 2161 2164 2165 2169

2164 HC -2.170000 -8.310000 -1.850000 48 2163

2165 CT -2.980000 -8.400000 -3.830000 51 2163 2166 2167 2168

2166 HC -3.680000 -7.870000 -3.250000 52 2165

2167 HC -2.440000 -7.580000 -4.310000 52 2165

2168 HC -3.420000 -9.140000 -4.480000 52 2165

2169 CT -3.380000 -10.070000 -1.920000 49 2163 2170 2171 2172

2170 HC -2.900000 -10.530000 -1.060000 50 2169

2171 HC -3.630000 -10.850000 -2.640000 50 2169

2172 CT -4.670000 -9.390000 -1.440000 53 2169 2173 2174 2175

2173 HC -5.140000 -10.300000 -1.120000 54 2172

2174 HC -4.410000 -8.660000 -0.700000 54 2172

2175 HC -5.230000 -8.970000 -2.270000 54 2172

2176 C -0.400000 -8.730000 -3.810000 43 2161 2177 2178

2177 O -0.520000 -8.630000 -5.030000 45 2176

2178 N 0.410000 -7.930000 -3.110000 122 2176 2179 2180

2179 H 0.410000 -8.060000 -2.110000 125 2178

2180 CT 1.220000 -6.900000 -3.710000 123 2178 2181 2182 2197

2181 H1 0.590000 -6.180000 -4.230000 127 2180

2182 CT 1.922694 -6.094296 -2.596653 128 2180 2183 2184 2185

2183 HC 1.890397 -6.656309 -1.669681 129 2182

2184 HC 1.337353 -5.195182 -2.409275 129 2182

2185 CA 3.358722 -5.672842 -2.835049 130 2182 2186 2195

2186 CA 3.652983 -4.593913 -3.691227 131 2185 2187 2188

2187 HA 2.855881 -4.084601 -4.215254 132 2186

2188 CA 4.992848 -4.216967 -3.907267 133 2186 2189 2190

2189 HA 5.242684 -3.421446 -4.593894 134 2188

2190 C 6.034991 -4.938414 -3.286665 135 2188 2191 2193

2191 OH 7.328572 -4.645784 -3.562438 136 2190 2192

2192 HO 7.903807 -5.378988 -3.338664 137 2191

2193 CA 5.739602 -5.998133 -2.408203 133 2190 2194 2195

2194 HA 6.533479 -6.553311 -1.932630 134 2193

2195 CA 4.401905 -6.358019 -2.181579 131 2185 2193 2196

2196 HA 4.190238 -7.190843 -1.528352 132 2195

2197 C 2.130000 -7.550000 -4.710000 124 2180 2198 2199

2198 O 2.280000 -7.060000 -5.820000 126 2197

2199 N 2.740000 -8.690000 -4.340000 27 2197 2200 2201

2200 H 2.550000 -9.060000 -3.420000 30 2199

2201 CT 3.640000 -9.360000 -5.230000 28 2199 2202 2203 2216

2202 H1 4.390000 -8.660000 -5.590000 32 2201

2203 CT 4.300000 -10.590000 -4.600000 33 2201 2204 2205 2206

2204 HC 4.870000 -11.110000 -5.370000 34 2203

2205 HC 3.540000 -11.280000 -4.240000 34 2203

2206 CT 5.260000 -10.250000 -3.440000 35 2203 2207 2208 2212

2207 HC 4.680000 -9.800000 -2.640000 36 2206

2208 CT 5.900000 -11.510000 -2.840000 37 2206 2209 2210 2211

2209 HC 6.540000 -11.230000 -2.010000 38 2208

2210 HC 5.120000 -12.170000 -2.470000 38 2208

2211 HC 6.490000 -12.020000 -3.600000 38 2208

2212 CT 6.300000 -9.200000 -3.870000 39 2206 2213 2214 2215

2213 HC 6.990000 -9.030000 -3.050000 40 2212

2214 HC 6.860000 -9.570000 -4.730000 40 2212

2215 HC 5.830000 -8.250000 -4.110000 40 2212

2216 C 2.890000 -9.800000 -6.440000 29 2201 2217 2218

2217 O 3.390000 -9.690000 -7.560000 31 2216

2218 N 1.660000 -10.320000 -6.260000 7 2216 2219 2220

2219 H 1.280000 -10.380000 -5.320000 10 2218

2220 CT 0.900000 -10.820000 -7.360000 8 2218 2221 2222 2226

2221 H1 1.470000 -11.590000 -7.880000 12 2220

2222 CT -0.460000 -11.400000 -6.940000 13 2220 2223 2224 2225

2223 HC -0.980000 -11.770000 -7.820000 14 2222

2224 HC -0.310000 -12.220000 -6.240000 14 2222

2225 HC -1.080000 -10.630000 -6.480000 14 2222

2226 C 0.630000 -9.700000 -8.320000 9 2220 2227 2228

2227 O 0.730000 -9.880000 -9.530000 11 2226

2228 N 0.290000 -8.510000 -7.800000 77 2226 2229 2230

2229 H 0.050000 -8.470000 -6.820000 80 2228

2230 CT -0.020000 -7.390000 -8.640000 78 2228 2231 2232 2237

2231 H1 -0.810000 -7.660000 -9.340000 82 2230

2232 CT -0.440000 -6.140000 -7.850000 83 2230 2233 2234 2235

2233 H1 -0.570000 -5.310000 -8.550000 84 2232

2234 H1 0.340000 -5.880000 -7.130000 84 2232

2235 SH -2.010000 -6.370000 -6.960000 85 2232 2236

2236 HS -2.130000 -5.120000 -6.510000 86 2235

2237 C 1.180000 -7.010000 -9.440000 79 2230 2238 2239

2238 O 1.080000 -6.760000 -10.640000 81 2237

2239 N 2.370000 -6.970000 -8.800000 27 2237 2240 2241

2240 H 2.390000 -7.210000 -7.810000 30 2239

2241 CT 3.540000 -6.560000 -9.510000 28 2239 2242 2243 2256

2242 H1 3.360000 -5.610000 -10.020000 32 2241

2243 CT 4.810000 -6.500000 -8.630000 33 2241 2244 2245 2246

2244 HC 5.650000 -6.250000 -9.280000 34 2243

2245 HC 5.000000 -7.490000 -8.200000 34 2243

2246 CT 4.750000 -5.480000 -7.490000 35 2243 2247 2248 2252

2247 HC 4.010000 -5.810000 -6.780000 36 2246

2248 CT 6.090000 -5.420000 -6.730000 37 2246 2249 2250 2251

2249 HC 6.470000 -4.420000 -6.670000 38 2248

2250 HC 6.100000 -6.060000 -5.850000 38 2248

2251 HC 6.850000 -5.850000 -7.380000 38 2248

2252 CT 4.280000 -4.100000 -7.980000 39 2246 2253 2254 2255

2253 HC 3.930000 -3.550000 -7.110000 40 2252

2254 HC 5.140000 -3.580000 -8.340000 40 2252

2255 HC 3.450000 -4.120000 -8.680000 40 2252

2256 C 3.820000 -7.540000 -10.600000 29 2241 2257 2258

2257 O 4.220000 -7.170000 -11.690000 31 2256

2258 N 3.590000 -8.840000 -10.310000 244 2256 2259 2260

2259 H 3.250000 -9.080000 -9.380000 247 2258

2260 CT 3.870000 -9.880000 -11.250000 245 2258 2261 2262 2273

2261 H1 4.930000 -9.860000 -11.520000 249 2260

2262 CT 3.490000 -11.270000 -10.690000 250 2260 2263 2264 2265

2263 HC 2.410000 -11.310000 -10.580000 251 2262

2264 HC 3.870000 -11.380000 -9.670000 251 2262

2265 CT 3.840000 -12.440000 -11.610000 252 2262 2266 2267 2268

2266 HC 3.820000 -12.160000 -12.660000 253 2265

2267 HC 3.130000 -13.250000 -11.440000 253 2265

2268 C 5.220000 -12.950000 -11.210000 254 2265 2269 2270

2269 O 5.640000 -12.810000 -10.060000 255 2268

2270 N 5.950000 -13.570000 -12.180000 256 2268 2271 2272

2271 H 6.860000 -13.870000 -11.870000 257 2270

2272 H 5.640000 -13.600000 -13.130000 257 2270

2273 C 3.030000 -9.670000 -12.470000 246 2260 2274 2275

2274 O 3.530000 -9.770000 -13.590000 248 2273

2275 N 1.740000 -9.340000 -12.280000 210 2273 2276 2277

2276 H 1.370000 -9.290000 -11.330000 213 2275

2277 CT 0.830000 -9.160000 -13.380000 211 2275 2278 2279 2285

2278 H1 0.850000 -10.050000 -14.010000 215 2277

2279 CT -0.610000 -8.900000 -12.910000 216 2277 2280 2281 2282

2280 HC -0.690000 -7.930000 -12.410000 217 2279

2281 HC -0.900000 -9.680000 -12.210000 217 2279

2282 C -1.530000 -8.950000 -14.120000 218 2279 2283 2284

2283 O2 -1.010000 -9.160000 -15.250000 219 2282

2284 O2 -2.760000 -8.780000 -13.940000 219 2282

2285 C 1.260000 -7.970000 -14.190000 212 2277 2286 2287

2286 O 1.250000 -8.010000 -15.420000 214 2285

2287 N 1.660000 -6.880000 -13.510000 41 2285 2288 2289

2288 H 1.580000 -6.900000 -12.500000 44 2287

2289 CT 2.060000 -5.690000 -14.210000 42 2287 2290 2291 2304

2290 H1 1.190000 -5.350000 -14.740000 46 2289

2291 CT 2.430000 -4.570000 -13.280000 47 2289 2292 2293 2297

2292 HC 3.220000 -4.900000 -12.610000 48 2291

2293 CT 2.980000 -3.410000 -14.130000 51 2291 2294 2295 2296

2294 HC 3.260000 -2.580000 -13.490000 52 2293

2295 HC 3.860000 -3.710000 -14.680000 52 2293

2296 HC 2.220000 -3.070000 -14.840000 52 2293

2297 CT 1.220000 -4.170000 -12.420000 49 2291 2298 2299 2300

2298 HC 0.770000 -4.980000 -11.900000 50 2297

2299 HC 0.440000 -3.800000 -13.090000 50 2297

2300 CT 1.550000 -3.030000 -11.460000 53 2297 2301 2302 2303

2301 HC 0.680000 -2.840000 -10.830000 54 2300

2302 HC 2.390000 -3.310000 -10.830000 54 2300

2303 HC 1.770000 -2.110000 -12.000000 54 2300

2304 C 3.270000 -6.030000 -15.000000 43 2289 2305 2306

2305 O 3.430000 -5.620000 -16.150000 45 2304

2306 N 4.200000 -6.780000 -14.390000 41 2304 2307 2308

2307 H 4.030000 -7.130000 -13.450000 44 2306

2308 CT 5.380000 -7.100000 -15.120000 42 2306 2309 2310 2323

2309 H1 5.800000 -6.190000 -15.540000 46 2308

2310 CT 6.420000 -7.770000 -14.290000 47 2308 2311 2312 2316

2311 HC 5.990000 -8.660000 -13.820000 48 2310

2312 CT 7.550000 -8.230000 -15.230000 51 2310 2313 2314 2315

2313 HC 8.350000 -8.690000 -14.660000 52 2312

2314 HC 7.230000 -9.020000 -15.910000 52 2312

2315 HC 7.950000 -7.390000 -15.790000 52 2312

2316 CT 6.900000 -6.830000 -13.170000 49 2310 2317 2318 2319

2317 HC 6.120000 -6.420000 -12.560000 50 2316

2318 HC 7.360000 -5.960000 -13.650000 50 2316

2319 CT 7.970000 -7.470000 -12.290000 53 2316 2320 2321 2322

2320 HC 8.220000 -6.780000 -11.480000 54 2319

2321 HC 7.590000 -8.400000 -11.860000 54 2319

2322 HC 8.880000 -7.670000 -12.850000 54 2319

2323 C 5.040000 -8.020000 -16.260000 43 2308 2324 2325

2324 O 5.480000 -7.760000 -17.360000 45 2323

2325 N 4.280000 -9.110000 -16.020000 55 2323 2326 2327

2326 H 3.920000 -9.220000 -15.090000 58 2325

2327 CT 3.960000 -10.120000 -17.010000 56 2325 2328 2329 2334

2328 H1 4.880000 -10.380000 -17.540000 60 2327

2329 CT 3.420000 -11.410000 -16.370000 61 2327 2330 2331 2332

2330 H1 3.190000 -12.140000 -17.150000 62 2329

2331 H1 2.510000 -11.190000 -15.800000 62 2329

2332 OH 4.380000 -11.960000 -15.480000 63 2329 2333

2333 HO 3.920000 -12.620000 -14.950000 64 2332

2334 C 2.940000 -9.730000 -18.050000 57 2327 2335 2336

2335 O 3.260000 -9.510000 -19.220000 59 2334

2336 N 1.660000 -9.660000 -17.660000 258 2334 2337 2338

2337 H 1.420000 -9.620000 -16.670000 261 2336

2338 CT 0.610000 -9.400000 -18.610000 259 2336 2339 2340 2351

2339 H1 0.660000 -10.110000 -19.430000 263 2338

2340 CT -0.790000 -9.470000 -17.970000 264 2338 2341 2342 2343

2341 HC -0.800000 -8.630000 -17.310000 265 2340

2342 HC -0.960000 -10.430000 -17.490000 265 2340

2343 CT -1.950000 -9.090000 -18.890000 266 2340 2344 2345 2346

2344 H1 -2.850000 -9.520000 -18.470000 267 2343

2345 H1 -1.800000 -9.560000 -19.860000 267 2343

2346 S -2.170000 -7.290000 -19.150000 268 2343 2347

2347 CT -3.710000 -7.480000 -20.090000 269 2346 2348 2349 2350

2348 H1 -3.590000 -6.940000 -21.020000 270 2347

2349 H1 -3.840000 -8.510000 -20.420000 270 2347

2350 H1 -4.540000 -7.080000 -19.600000 270 2347

2351 C 0.800000 -8.030000 -19.150000 260 2338 2352 2353

2352 O 0.680000 -7.770000 -20.340000 262 2351

2353 N 1.080000 -7.110000 -18.230000 107 2351 2354 2355

2354 H 1.050000 -7.400000 -17.250000 110 2353

2355 CT 1.320000 -5.740000 -18.540000 108 2353 2356 2357 2371

2356 H1 0.620000 -5.430000 -19.320000 112 2355

2357 CT 1.150000 -4.780000 -17.360000 113 2355 2358 2359 2360

2358 HC 1.610000 -3.800000 -17.480000 114 2357

2359 HC 1.420000 -5.310000 -16.540000 114 2357

2360 CA -0.310000 -4.730000 -17.060000 115 2357 2361 2369

2361 CA -0.910000 -5.710000 -16.300000 116 2360 2362 2363

2362 HA -0.380000 -6.560000 -15.920000 117 2361

2363 CA -2.260000 -5.660000 -16.030000 118 2361 2364 2365

2364 HA -2.690000 -6.460000 -15.450000 119 2363

2365 CA -3.030000 -4.620000 -16.520000 120 2363 2366 2367

2366 HA -4.080000 -4.570000 -16.290000 121 2365

2367 CA -2.430000 -3.640000 -17.280000 118 2365 2368 2369

2368 HA -3.030000 -2.810000 -17.440000 119 2367

2369 CA -1.090000 -3.700000 -17.540000 116 2360 2367 2370

2370 HA -0.610000 -2.810000 -17.880000 117 2369

2371 C 2.690000 -5.670000 -19.150000 109 2355 2372 2373

2372 O 3.090000 -4.580000 -19.540000 111 2371

2373 N 3.480000 -6.780000 -19.120000 244 2371 2374 2375

2374 H 3.150000 -7.610000 -18.650000 247 2373

2375 CT 4.710000 -6.850000 -19.890000 245 2373 2376 2377 2388

2376 H1 5.380000 -6.030000 -19.640000 249 2375

2377 CT 5.480000 -8.180000 -20.020000 250 2375 2378 2379 2380

2378 HC 4.890000 -8.850000 -20.640000 251 2377

2379 HC 5.690000 -8.790000 -19.170000 251 2377

2380 CT 6.750000 -8.080000 -20.860000 252 2377 2381 2382 2383

2381 HC 7.440000 -7.380000 -20.400000 253 2380

2382 HC 6.540000 -7.780000 -21.860000 253 2380

2383 C 7.390000 -9.460000 -20.920000 254 2380 2384 2385

2384 O 6.810000 -10.440000 -20.450000 255 2383

2385 N 8.610000 -9.550000 -21.510000 256 2383 2386 2387

2386 H 8.950000 -10.500000 -21.570000 257 2385

2387 H 9.030000 -8.770000 -21.980000 257 2385

2388 C 4.220000 -6.740000 -21.260000 246 2375 2389 2390

2389 O 4.880000 -6.200000 -22.140000 248 2388

2390 N 2.970000 -7.230000 -21.480000 271 2388 2391 2392

2391 H 2.450000 -7.700000 -20.740000 274 2390

2392 CT 2.320000 -6.920000 -22.710000 272 2390 2393 2394 2410

2393 H1 3.010000 -7.030000 -23.520000 276 2392

2394 CT 1.080000 -7.780000 -23.040000 277 2392 2395 2396 2397

2395 HC 1.120000 -7.550000 -24.050000 278 2394

2396 HC 0.120000 -7.530000 -22.590000 278 2394

2397 CT 1.400000 -9.270000 -23.240000 279 2394 2398 2399 2400

2398 HC 2.040000 -9.600000 -22.420000 280 2397

2399 HC 1.950000 -9.420000 -24.170000 280 2397

2400 CT 0.150000 -10.150000 -23.230000 281 2397 2401 2402 2403

2401 HC -0.520000 -9.790000 -24.020000 282 2400

2402 HC -0.360000 -10.010000 -22.270000 282 2400

2403 CT 0.440000 -11.640000 -23.440000 283 2400 2404 2405 2406

2404 HP 1.120000 -11.980000 -22.650000 284 2403

2405 HP 0.940000 -11.760000 -24.410000 284 2403

2406 N3 -0.820000 -12.410000 -23.410000 285 2403 2407 2408 2409

2407 H -0.660000 -13.390000 -23.560000 286 2406

2408 H -1.290000 -12.260000 -22.530000 286 2406

2409 H -1.440000 -12.060000 -24.140000 286 2406

2410 C 1.850000 -5.460000 -22.600000 273 2392 2411 2412

2411 O 0.740000 -5.100000 -22.970000 275 2410

2412 N 2.770000 -4.620000 -22.070000 7 2410 2413 2414

2413 H 3.480000 -5.060000 -21.520000 10 2412

2414 CT 3.000000 -3.210000 -22.100000 8 2412 2415 2416 2420

2415 H1 2.110000 -2.600000 -22.090000 12 2414

2416 CT 4.160000 -2.720000 -21.220000 13 2414 2417 2418 2419

2417 HC 4.500000 -1.760000 -21.610000 14 2416

2418 HC 3.840000 -2.510000 -20.200000 14 2416

2419 HC 5.000000 -3.420000 -21.240000 14 2416

2420 C 3.500000 -3.220000 -23.500000 9 2414 2421 2422

2421 O 3.500000 -2.210000 -24.190000 11 2420

2422 N 4.090000 -4.390000 -23.850000 41 2420 2423 2424

2423 H 4.220000 -5.060000 -23.120000 44 2422

2424 CT 4.510000 -4.880000 -25.140000 42 2422 2425 2426 2439

2425 H1 5.180000 -4.160000 -25.510000 46 2424

2426 CT 5.240000 -6.190000 -25.060000 47 2424 2427 2428 2432

2427 HC 4.650000 -6.940000 -24.550000 48 2426

2428 CT 5.410000 -6.730000 -26.490000 51 2426 2429 2430 2431

2429 HC 5.940000 -7.680000 -26.460000 52 2428

2430 HC 4.480000 -6.990000 -26.990000 52 2428

2431 HC 6.000000 -6.070000 -27.110000 52 2428

2432 CT 6.560000 -6.030000 -24.300000 49 2426 2433 2434 2435

2433 HC 6.550000 -5.480000 -23.370000 50 2432

2434 HC 7.180000 -5.380000 -24.920000 50 2432

2435 CT 7.340000 -7.350000 -24.200000 53 2432 2436 2437 2438

2436 HC 8.150000 -7.240000 -23.480000 54 2435

2437 HC 6.680000 -8.170000 -23.920000 54 2435

2438 HC 7.780000 -7.600000 -25.170000 54 2435

2439 C 3.270000 -5.090000 -25.980000 43 2424 2440 2441

2440 O 3.300000 -4.950000 -27.200000 45 2439

2441 N 2.160000 -5.540000 -25.360000 210 2439 2442 2443

2442 H 2.120000 -5.760000 -24.400000 213 2441

2443 CT 0.920000 -5.560000 -26.080000 211 2441 2444 2445 2451

2444 H1 1.020000 -6.160000 -26.970000 215 2443

2445 CT -0.280000 -6.120000 -25.290000 216 2443 2446 2447 2448

2446 HC -0.710000 -5.510000 -24.510000 217 2445

2447 HC -0.280000 -7.190000 -25.160000 217 2445

2448 C -1.470000 -6.220000 -26.240000 218 2445 2449 2450

2449 O2 -1.330000 -5.770000 -27.410000 219 2448

2450 O2 -2.520000 -6.770000 -25.810000 219 2448

2451 C 0.700000 -4.110000 -26.340000 212 2443 2452 2453

2452 O 0.140000 -3.700000 -27.360000 214 2451

2453 N 1.140000 -3.290000 -25.370000 1 2451 2454 2455

2454 H 1.270000 -3.720000 -24.480000 4 2453

2455 CT 1.140000 -1.870000 -25.510000 2 2453 2456 2457 2458

2456 H1 1.390000 -1.350000 -24.600000 6 2455

2457 H1 0.140000 -1.540000 -25.820000 6 2455

2458 C 2.100000 -1.550000 -26.620000 3 2455 2459 2460

2459 O 1.940000 -0.560000 -27.330000 5 2458

2460 N 3.160000 -2.380000 -26.760000 232 2458 2461 2462

2461 H 3.190000 -3.130000 -26.110000 235 2460

2462 CT 4.170000 -2.240000 -27.770000 233 2460 2463 2464 2473

2463 H1 4.430000 -1.240000 -27.920000 237 2462

2464 CT 5.360000 -3.220000 -27.620000 238 2462 2465 2466 2467

2465 HC 5.240000 -4.270000 -27.700000 239 2464

2466 HC 5.840000 -2.910000 -26.690000 239 2464

2467 CT 6.530000 -2.940000 -28.570000 240 2464 2468 2469 2470

2468 HC 7.440000 -3.410000 -28.170000 241 2467

2469 HC 6.710000 -1.860000 -28.630000 241 2467

2470 C 6.200000 -3.540000 -29.930000 242 2467 2471 2472

2471 O2 5.840000 -4.750000 -29.970000 243 2470

2472 O2 6.310000 -2.800000 -30.950000 243 2470

2473 C 3.490000 -2.470000 -29.080000 234 2462 2474 2475

2474 O 3.940000 -2.000000 -30.120000 236 2473

2475 N 2.380000 -3.220000 -29.050000 15 2473 2476 2477

2476 H 2.040000 -3.620000 -28.190000 18 2475

2477 CT 1.620000 -3.400000 -30.260000 16 2475 2478 2479 2489

2478 H1 2.240000 -3.860000 -31.030000 20 2477

2479 CT 0.350000 -4.170000 -30.050000 21 2477 2480 2481 2485

2480 HC -0.360000 -3.630000 -29.450000 22 2479

2481 CT -0.430000 -4.190000 -31.380000 23 2479 2482 2483 2484

2482 HC -1.300000 -4.840000 -31.260000 24 2481

2483 HC -0.810000 -3.200000 -31.630000 24 2481

2484 HC 0.200000 -4.570000 -32.180000 24 2481

2485 CT 0.700000 -5.570000 -29.510000 25 2479 2486 2487 2488

2486 HC -0.190000 -6.150000 -29.330000 26 2485

2487 HC 1.250000 -6.090000 -30.290000 26 2485

2488 HC 1.380000 -5.500000 -28.690000 26 2485

2489 C 1.250000 -2.020000 -30.680000 17 2477 2490 2491

2490 O 1.190000 -1.720000 -31.870000 19 2489

2491 N 1.010000 -1.140000 -29.700000 210 2489 2492 2493

2492 H 1.070000 -1.460000 -28.750000 213 2491

2493 CT 0.690000 0.240000 -29.940000 211 2491 2494 2495 2501

2494 H1 0.000000 0.300000 -30.780000 215 2493

2495 CT 0.100000 0.970000 -28.730000 216 2493 2496 2497 2498

2496 HC -0.100000 2.000000 -29.040000 217 2495

2497 HC 0.680000 1.130000 -27.840000 217 2495

2498 C -1.300000 0.430000 -28.480000 218 2495 2499 2500

2499 O2 -1.790000 -0.360000 -29.340000 219 2498

2500 O2 -1.900000 0.790000 -27.440000 219 2498

2501 C 1.960000 0.930000 -30.340000 212 2493 2502 2503

2502 O 2.990000 0.310000 -30.550000 214 2501

2503 N 1.890000 2.270000 -30.470000 220 2501 2504 2505

2504 H 0.990000 2.680000 -30.270000 223 2503

2505 CT 3.010000 3.040000 -30.930000 221 2503 2506 2507 2515

2506 H1 3.310000 2.610000 -31.890000 225 2505

2507 CT 2.670000 4.530000 -31.110000 226 2505 2508 2509 2510

2508 HC 2.510000 5.000000 -30.140000 227 2507

2509 HC 1.740000 4.610000 -31.680000 227 2507

2510 C 3.800000 5.210000 -31.880000 228 2507 2511 2512

2511 O 4.840000 5.540000 -31.320000 229 2510

2512 N 3.570000 5.420000 -33.200000 230 2510 2513 2514

2513 H 4.260000 5.930000 -33.720000 231 2512

2514 H 2.690000 5.140000 -33.620000 231 2512

2515 C 4.160000 2.900000 -29.970000 222 2505 2516 2517

2516 O 3.980000 2.600000 -28.790000 224 2515

2517 N 5.390000 3.120000 -30.490000 271 2515 2518 2519

2518 H 5.410000 3.320000 -31.480000 274 2517

2519 CT 6.610000 2.990000 -29.760000 272 2517 2520 2521 2537

2520 H1 6.650000 1.980000 -29.330000 276 2519

2521 CT 7.850000 3.270000 -30.630000 277 2519 2522 2523 2524

2522 HC 7.780000 4.270000 -31.070000 278 2521

2523 HC 7.840000 2.540000 -31.440000 278 2521

2524 CT 9.180000 3.140000 -29.870000 279 2521 2525 2526 2527

2525 HC 9.170000 2.250000 -29.240000 280 2524

2526 HC 9.230000 4.040000 -29.280000 280 2524

2527 CT 10.400000 3.120000 -30.790000 281 2524 2528 2529 2530

2528 HC 10.400000 4.030000 -31.390000 282 2527

2529 HC 10.300000 2.270000 -31.470000 282 2527

2530 CT 11.730000 3.000000 -30.040000 283 2527 2531 2532 2533

2531 HP 11.720000 2.080000 -29.440000 284 2530

2532 HP 11.860000 3.860000 -29.380000 284 2530

2533 N3 12.850000 2.980000 -31.000000 285 2530 2534 2535 2536

2534 H 13.740000 2.890000 -30.530000 286 2533

2535 H 12.740000 2.200000 -31.640000 286 2533

2536 H 12.860000 3.820000 -31.560000 286 2533

2537 C 6.630000 3.980000 -28.640000 273 2519 2538 2539

2538 O 7.080000 3.680000 -27.530000 275 2537

2539 N 6.130000 5.200000 -28.890000 15 2537 2540 2541

2540 H 5.730000 5.380000 -29.810000 18 2539

2541 CT 6.130000 6.220000 -27.880000 16 2539 2542 2543 2553

2542 H1 7.130000 6.400000 -27.510000 20 2541

2543 CT 5.580000 7.530000 -28.370000 21 2541 2544 2545 2549

2544 HC 5.600000 8.250000 -27.550000 22 2543

2545 CT 6.500000 8.050000 -29.490000 23 2543 2546 2547 2548

2546 HC 6.150000 9.030000 -29.810000 24 2545

2547 HC 7.520000 8.160000 -29.120000 24 2545

2548 HC 6.490000 7.390000 -30.350000 24 2545

2549 CT 4.120000 7.320000 -28.830000 25 2543 2550 2551 2552

2550 HC 3.720000 8.340000 -28.820000 26 2549

2551 HC 3.940000 6.460000 -29.440000 26 2549

2552 HC 3.480000 7.130000 -27.980000 26 2549

2553 C 5.290000 5.760000 -26.740000 17 2541 2554 2555

2554 O 5.640000 5.950000 -25.570000 19 2553

2555 N 4.140000 5.120000 -27.040000 649 2553 2556 2557

2556 H 3.910000 4.900000 -28.000000 652 2555

2557 CT 3.250000 4.670000 -26.020000 650 2555 2558 2559 2566

2558 H1 2.950000 5.530000 -25.420000 654 2557

2559 CT 2.010000 3.970000 -26.610000 655 2557 2560 2561 2562

2560 HC 2.250000 2.970000 -26.980000 656 2559

2561 HC 1.600000 4.570000 -27.420000 656 2559

2562 C 0.950000 3.850000 -25.530000 657 2559 2563 2564

2563 O 1.190000 4.380000 -24.410000 658 2562

2564 OH -0.110000 3.240000 -25.810000 659 2562 2565

2565 HO -0.130000 2.460000 -26.380000 660 2564

2566 C 4.000000 3.670000 -25.200000 651 2557 2567 2568

2567 O 3.920000 3.670000 -23.980000 653 2566

2568 N 4.790000 2.810000 -25.870000 65 2566 2569 2570

2569 H 4.640000 2.710000 -26.870000 68 2568

2570 CT 5.500000 1.760000 -25.200000 66 2568 2571 2572 2580

2571 H1 4.790000 1.120000 -24.680000 70 2570

2572 CT 6.310000 0.930000 -26.160000 71 2570 2573 2574 2578

2573 H1 7.080000 1.520000 -26.640000 72 2572

2574 CT 7.000000 -0.200000 -25.370000 75 2572 2575 2576 2577

2575 HC 7.510000 -0.860000 -26.080000 76 2574

2576 HC 7.730000 0.180000 -24.660000 76 2574

2577 HC 6.240000 -0.790000 -24.850000 76 2574

2578 OH 5.470000 0.380000 -27.160000 73 2572 2579

2579 HO 5.930000 -0.180000 -27.790000 74 2578

2580 C 6.460000 2.360000 -24.220000 67 2570 2581 2582

2581 O 6.600000 1.870000 -23.100000 69 2580

2582 N 7.150000 3.440000 -24.610000 138 2580 2583 2584

2583 H 6.960000 3.810000 -25.540000 141 2582

2584 CT 8.130000 4.020000 -23.730000 139 2582 2585 2586 2604

2585 H1 8.860000 3.270000 -23.450000 143 2584

2586 CT 8.850000 5.240000 -24.350000 144 2584 2587 2588 2589

2587 HC 9.410000 5.760000 -23.570000 145 2586

2588 HC 8.110000 5.950000 -24.730000 145 2586

2589 C* 9.790000 4.880000 -25.470000 146 2586 2590 2603

2590 CW 9.550000 4.750000 -26.810000 147 2589 2591 2592

2591 H4 8.650000 5.090000 -27.270000 148 2590

2592 NA 10.710000 4.420000 -27.460000 150 2590 2593 2594

2593 H 10.760000 4.410000 -28.460000 151 2592

2594 CN 11.730000 4.350000 -26.540000 152 2592 2595 2603

2595 CA 13.060000 4.070000 -26.690000 155 2594 2596 2597

2596 HA 13.490000 3.870000 -27.650000 156 2595

2597 CA 13.840000 4.070000 -25.560000 159 2595 2598 2599

2598 HA 14.900000 3.860000 -25.640000 160 2597

2599 CA 13.310000 4.350000 -24.310000 157 2597 2600 2601

2600 HA 13.970000 4.360000 -23.450000 158 2599

2601 CA 11.970000 4.640000 -24.160000 153 2599 2602 2603

2602 HA 11.550000 4.890000 -23.200000 154 2601

2603 CB 11.190000 4.630000 -25.290000 149 2589 2594 2601

2604 C 7.450000 4.480000 -22.480000 140 2584 2605 2606

2605 O 7.950000 4.250000 -21.380000 142 2604

2606 N 6.280000 5.150000 -22.610000 65 2604 2607 2608

2607 H 5.850000 5.240000 -23.530000 68 2606

2608 CT 5.620000 5.680000 -21.460000 66 2606 2609 2610 2618

2609 H1 6.330000 6.290000 -20.900000 70 2608

2610 CT 4.440000 6.570000 -21.810000 71 2608 2611 2612 2616

2611 H1 4.740000 7.260000 -22.600000 72 2610

2612 CT 3.260000 5.730000 -22.290000 75 2610 2613 2614 2615

2613 HC 2.360000 6.320000 -22.130000 76 2612

2614 HC 3.250000 5.630000 -23.350000 76 2612

2615 HC 3.030000 4.970000 -21.610000 76 2612

2616 OH 4.060000 7.340000 -20.680000 73 2610 2617

2617 HO 3.190000 7.690000 -20.900000 74 2616

2618 C 5.170000 4.580000 -20.550000 67 2608 2619 2620

2619 O 5.300000 4.680000 -19.340000 69 2618

2620 N 4.630000 3.470000 -21.100000 27 2618 2621 2622

2621 H 4.410000 3.460000 -22.100000 30 2620

2622 CT 4.180000 2.400000 -20.250000 28 2620 2623 2624 2637

2623 H1 3.470000 2.790000 -19.520000 32 2622

2624 CT 3.500000 1.240000 -21.010000 33 2622 2625 2626 2627

2625 HC 3.490000 0.370000 -20.350000 34 2624

2626 HC 4.080000 0.970000 -21.890000 34 2624

2627 CT 2.040000 1.550000 -21.400000 35 2624 2628 2629 2633

2628 HC 1.550000 1.780000 -20.460000 36 2627

2629 CT 1.940000 2.760000 -22.320000 37 2627 2630 2631 2632

2630 HC 1.250000 2.490000 -23.110000 38 2629

2631 HC 1.720000 3.690000 -21.900000 38 2629

2632 HC 2.800000 2.490000 -22.810000 38 2629

2633 CT 1.360000 0.310000 -21.990000 39 2627 2634 2635 2636

2634 HC 0.380000 0.580000 -22.150000 40 2633

2635 HC 1.830000 0.060000 -22.940000 40 2633

2636 HC 1.630000 -0.430000 -21.280000 40 2633

2637 C 5.340000 1.820000 -19.490000 29 2622 2638 2639

2638 O 5.210000 1.500000 -18.310000 31 2637

2639 N 6.500000 1.670000 -20.150000 287 2637 2640 2641

2640 H 6.500000 1.900000 -21.140000 290 2639

2641 CT 7.630000 1.090000 -19.490000 288 2639 2642 2643 2661

2642 H1 7.370000 0.100000 -19.110000 292 2641

2643 CT 8.860000 1.020000 -20.420000 293 2641 2644 2645 2646

2644 HC 9.090000 2.020000 -20.790000 294 2643

2645 HC 8.600000 0.400000 -21.280000 294 2643

2646 CT 10.110000 0.450000 -19.740000 295 2643 2647 2648 2649

2647 HC 9.930000 -0.580000 -19.450000 296 2646

2648 HC 10.330000 1.010000 -18.830000 296 2646

2649 CT 11.370000 0.520000 -20.590000 297 2646 2650 2651 2652

2650 H1 12.210000 0.160000 -19.990000 298 2649

2651 H1 11.590000 1.550000 -20.850000 298 2649

2652 N2 11.190000 -0.390000 -21.760000 299 2649 2653 2654

2653 H 11.280000 -1.390000 -21.640000 300 2652

2654 CA 10.930000 0.120000 -23.000000 301 2652 2655 2658

2655 N2 10.870000 1.470000 -23.190000 302 2654 2656 2657

2656 H 10.690000 1.850000 -24.110000 303 2655

2657 H 11.000000 2.080000 -22.410000 303 2655

2658 N2 10.740000 -0.720000 -24.060000 302 2654 2659 2660

2659 H 10.470000 -0.350000 -24.950000 303 2658

2660 H 10.730000 -1.720000 -23.930000 303 2658

2661 C 8.030000 1.940000 -18.330000 289 2641 2662 2663

2662 O 8.270000 1.440000 -17.230000 291 2661

2663 N 8.080000 3.270000 -18.530000 287 2661 2664 2665

2664 H 7.810000 3.600000 -19.450000 290 2663

2665 CT 8.500000 4.160000 -17.500000 288 2663 2666 2667 2685

2666 H1 9.480000 3.860000 -17.110000 292 2665

2667 CT 8.500000 5.630000 -17.960000 293 2665 2668 2669 2670

2668 HC 8.770000 6.260000 -17.110000 294 2667

2669 HC 7.500000 5.900000 -18.290000 294 2667

2670 CT 9.470000 5.900000 -19.120000 295 2667 2671 2672 2673

2671 HC 9.340000 5.200000 -19.920000 296 2670

2672 HC 10.480000 5.720000 -18.760000 296 2670

2673 CT 9.390000 7.330000 -19.670000 297 2670 2674 2675 2676

2674 H1 8.350000 7.620000 -19.830000 298 2673

2675 H1 9.890000 7.340000 -20.640000 298 2673

2676 N2 10.140000 8.210000 -18.730000 299 2673 2677 2678

2677 H 10.530000 7.830000 -17.880000 300 2676

2678 CA 10.290000 9.530000 -19.020000 301 2676 2679 2682

2679 N2 9.730000 10.050000 -20.160000 302 2678 2680 2681

2680 H 9.820000 11.020000 -20.390000 303 2679

2681 H 9.180000 9.450000 -20.750000 303 2679

2682 N2 10.990000 10.340000 -18.180000 302 2678 2683 2684

2683 H 11.100000 11.310000 -18.400000 303 2682

2684 H 11.400000 9.970000 -17.340000 303 2682

2685 C 7.540000 4.080000 -16.360000 289 2665 2686 2687

2686 O 7.930000 3.960000 -15.200000 291 2685

2687 N 6.240000 4.100000 -16.690000 77 2685 2688 2689

2688 H 5.990000 4.180000 -17.670000 80 2687

2689 CT 5.190000 4.130000 -15.720000 78 2687 2690 2691 2696

2690 H1 5.320000 4.990000 -15.060000 82 2689

2691 CT 3.830000 4.220000 -16.430000 83 2689 2692 2693 2694

2692 H1 3.550000 3.240000 -16.830000 84 2691

2693 H1 3.920000 4.910000 -17.270000 84 2691

2694 SH 2.500000 4.850000 -15.370000 85 2691 2695

2695 HS 1.520000 4.760000 -16.280000 86 2694

2696 C 5.240000 2.880000 -14.900000 79 2689 2697 2698

2697 O 5.050000 2.900000 -13.690000 81 2696

2698 N 5.510000 1.740000 -15.560000 287 2696 2699 2700

2699 H 5.600000 1.770000 -16.570000 290 2698

2700 CT 5.560000 0.480000 -14.870000 288 2698 2701 2702 2720

2701 H1 4.620000 0.300000 -14.350000 292 2700

2702 CT 5.880000 -0.690000 -15.820000 293 2700 2703 2704 2705

2703 HC 6.800000 -0.420000 -16.340000 294 2702

2704 HC 5.070000 -0.770000 -16.550000 294 2702

2705 CT 6.040000 -2.030000 -15.110000 295 2702 2706 2707 2708

2706 HC 5.210000 -2.190000 -14.440000 296 2705

2707 HC 6.930000 -1.980000 -14.480000 296 2705

2708 CT 6.270000 -3.190000 -16.070000 297 2705 2709 2710 2711

2709 H1 5.320000 -3.490000 -16.510000 298 2708

2710 H1 6.690000 -4.020000 -15.500000 298 2708

2711 N2 7.170000 -2.690000 -17.140000 299 2708 2712 2713

2712 H 7.090000 -1.730000 -17.440000 300 2711

2713 CA 8.120000 -3.500000 -17.680000 301 2711 2714 2717

2714 N2 8.260000 -4.790000 -17.240000 302 2713 2715 2716

2715 H 8.930000 -5.420000 -17.630000 303 2714

2716 H 7.560000 -5.140000 -16.600000 303 2714

2717 N2 8.940000 -3.020000 -18.660000 302 2713 2718 2719

2718 H 9.680000 -3.600000 -19.000000 303 2717

2719 H 8.890000 -2.040000 -18.860000 303 2717

2720 C 6.640000 0.540000 -13.840000 289 2700 2721 2722

2721 O 6.480000 0.040000 -12.730000 291 2720

2722 N 7.790000 1.140000 -14.190000 210 2720 2723 2724

2723 H 7.900000 1.460000 -15.140000 213 2722

2724 CT 8.920000 1.220000 -13.310000 211 2722 2725 2726 2732

2725 H1 9.160000 0.220000 -12.940000 215 2724

2726 CT 10.150000 1.820000 -13.990000 216 2724 2727 2728 2729

2727 HC 10.950000 1.930000 -13.260000 217 2726

2728 HC 9.940000 2.790000 -14.430000 217 2726

2729 C 10.590000 0.830000 -15.060000 218 2726 2730 2731

2730 O2 10.480000 -0.390000 -14.800000 219 2729

2731 O2 11.030000 1.280000 -16.150000 219 2729

2732 C 8.600000 2.060000 -12.110000 212 2724 2733 2734

2733 O 8.940000 1.700000 -10.980000 214 2732

2734 N 7.910000 3.200000 -12.300000 41 2732 2735 2736

2735 H 7.660000 3.460000 -13.250000 44 2734

2736 CT 7.650000 4.020000 -11.150000 42 2734 2737 2738 2751

2737 H1 8.590000 4.250000 -10.650000 46 2736

2738 CT 6.999460 5.357226 -11.576610 47 2736 2739 2740 2744

2739 HC 6.116615 5.123292 -12.170768 48 2738

2740 CT 6.575582 6.183391 -10.343492 51 2738 2741 2742 2743

2741 HC 5.818472 5.650050 -9.768698 52 2740

2742 HC 7.438136 6.368605 -9.702899 52 2740

2743 HC 6.146290 7.137934 -10.644936 52 2740

2744 CT 7.973064 6.196297 -12.442531 49 2738 2745 2746 2747

2745 HC 8.493011 5.551916 -13.149634 50 2744

2746 HC 8.730236 6.650071 -11.801654 50 2744

2747 CT 7.285464 7.294004 -13.266720 53 2744 2748 2749 2750

2748 HC 8.033330 7.814960 -13.864435 54 2747

2749 HC 6.550616 6.847554 -13.937119 54 2747

2750 HC 6.794817 8.016832 -12.616165 54 2747

2751 C 6.810000 3.240000 -10.210000 43 2736 2752 2753

2752 O 7.050000 3.250000 -9.010000 45 2751

2753 N 5.790000 2.540000 -10.740000 65 2751 2754 2755

2754 H 5.660000 2.470000 -11.750000 68 2753

2755 CT 4.900000 1.890000 -9.840000 66 2753 2756 2757 2765

2756 H1 4.480000 2.630000 -9.170000 70 2755

2757 CT 3.677602 1.237254 -10.510274 71 2755 2758 2759 2763

2758 H1 3.077401 0.789849 -9.716665 72 2757

2759 CT 2.789518 2.258198 -11.222606 75 2757 2760 2761 2762

2760 HC 1.777193 1.862919 -11.275556 76 2759

2761 HC 2.764100 3.195871 -10.671375 76 2759

2762 HC 3.145457 2.447262 -12.230724 76 2759

2763 OH 4.011899 0.215726 -11.422346 73 2757 2764

2764 HO 4.776317 0.479557 -11.951472 74 2763

2765 C 5.620000 0.900000 -8.980000 67 2755 2766 2767

2766 O 5.600000 1.050000 -7.760000 69 2765

2767 N 6.370000 -0.050000 -9.570000 41 2765 2768 2769

2768 H 6.380000 -0.100000 -10.590000 44 2767

2769 CT 6.850000 -1.120000 -8.740000 42 2767 2770 2771 2784

2770 H1 5.990000 -1.550000 -8.230000 46 2769

2771 CT 7.560000 -2.270000 -9.440000 47 2769 2772 2773 2777

2772 HC 7.820000 -3.000000 -8.670000 48 2771

2773 CT 6.540000 -2.930000 -10.380000 51 2771 2774 2775 2776

2774 HC 6.790000 -3.980000 -10.550000 52 2773

2775 HC 5.500000 -2.820000 -10.100000 52 2773

2776 HC 6.580000 -2.420000 -11.340000 52 2773

2777 CT 8.860000 -1.840000 -10.130000 49 2771 2778 2779 2780

2778 HC 9.440000 -1.030000 -9.810000 50 2777

2779 HC 8.490000 -1.280000 -10.990000 50 2777

2780 CT 9.550000 -3.020000 -10.820000 53 2777 2781 2782 2783

2781 HC 10.470000 -2.670000 -11.280000 54 2780

2782 HC 9.780000 -3.790000 -10.090000 54 2780

2783 HC 8.920000 -3.440000 -11.600000 54 2780

2784 C 7.740000 -0.620000 -7.650000 43 2769 2785 2786

2785 O 7.610000 -1.030000 -6.490000 45 2784

2786 N 8.660000 0.290000 -7.960000 107 2784 2787 2788

2787 H 8.730000 0.650000 -8.910000 110 2786

2788 CT 9.520000 0.740000 -6.910000 108 2786 2789 2790 2804

2789 H1 9.880000 -0.120000 -6.340000 112 2788

2790 CT 10.740000 1.480000 -7.470000 113 2788 2791 2792 2793

2791 HC 11.200000 2.090000 -6.690000 114 2790

2792 HC 10.460000 2.110000 -8.310000 114 2790

2793 CA 11.690000 0.400000 -7.900000 115 2790 2794 2802

2794 CA 11.230000 -0.780000 -8.450000 116 2793 2795 2796

2795 HA 10.330000 -1.260000 -8.150000 117 2794

2796 CA 12.100000 -1.770000 -8.820000 118 2794 2797 2798

2797 HA 11.740000 -2.730000 -9.150000 119 2796

2798 CA 13.450000 -1.590000 -8.640000 120 2796 2799 2800

2799 HA 14.140000 -2.380000 -8.920000 121 2798

2800 CA 13.920000 -0.430000 -8.070000 118 2798 2801 2802

2801 HA 14.980000 -0.300000 -7.920000 119 2800

2802 CA 13.040000 0.550000 -7.690000 116 2793 2800 2803

2803 HA 13.420000 1.460000 -7.240000 117 2802

2804 C 8.770000 1.570000 -5.880000 109 2788 2805 2806

2805 O 8.970000 1.370000 -4.690000 111 2804

2806 N 7.890000 2.500000 -6.300000 7 2804 2807 2808

2807 H 7.710000 2.590000 -7.300000 10 2806

2808 CT 7.180000 3.370000 -5.380000 8 2806 2809 2810 2814

2809 H1 7.910000 3.860000 -4.730000 12 2808

2810 CT 6.453686 4.466416 -6.170730 13 2808 2811 2812 2813

2811 HC 5.964502 5.152356 -5.478890 14 2810

2812 HC 7.169566 5.022875 -6.776885 14 2810

2813 HC 5.704003 4.024425 -6.827855 14 2810

2814 C 6.230000 2.590000 -4.510000 9 2808 2815 2816

2815 O 6.150000 2.810000 -3.300000 11 2814

2816 N 5.500000 1.630000 -5.100000 138 2814 2817 2818

2817 H 5.620000 1.460000 -6.090000 141 2816

2818 CT 4.520000 0.890000 -4.350000 139 2816 2819 2820 2838

2819 H1 3.790000 1.560000 -3.910000 143 2818

2820 CT 3.802701 -0.154431 -5.235135 144 2818 2821 2822 2823

2821 HC 3.626708 -1.044322 -4.627287 145 2820

2822 HC 4.472025 -0.481134 -6.031585 145 2820

2823 C* 2.472135 0.186754 -5.842159 146 2820 2824 2837

2824 CW 1.393667 -0.631680 -5.804511 147 2823 2825 2826

2825 H4 1.349616 -1.601334 -5.324301 148 2824

2826 NA 0.362262 -0.089091 -6.540570 150 2824 2827 2828

2827 H -0.547452 -0.557427 -6.648536 151 2826

2828 CN 0.748491 1.080055 -7.157198 152 2826 2829 2837

2829 CA 0.102591 1.942452 -8.054869 155 2828 2830 2831

2830 HA -0.880025 1.685247 -8.427934 156 2829

2831 CA 0.768044 3.104986 -8.480064 159 2829 2832 2833

2832 HA 0.309150 3.766021 -9.200720 160 2831

2833 CA 2.049838 3.400814 -7.985847 157 2831 2834 2835

2834 HA 2.556217 4.292799 -8.329788 158 2833

2835 CA 2.693706 2.518180 -7.097399 153 2833 2836 2837

2836 HA 3.695596 2.729805 -6.762763 154 2835

2837 CB 2.071137 1.323860 -6.672288 149 2823 2828 2835

2838 C 5.220000 0.140000 -3.260000 140 2818 2839 2840

2839 O 4.710000 0.040000 -2.140000 142 2838

2840 N 6.410000 -0.410000 -3.560000 55 2838 2841 2842

2841 H 6.850000 -0.230000 -4.460000 58 2840

2842 CT 7.080000 -1.290000 -2.640000 56 2840 2843 2844 2849

2843 H1 6.400000 -2.110000 -2.400000 60 2842

2844 CT 8.370122 -1.843269 -3.265458 61 2842 2845 2846 2847

2845 H1 8.167610 -2.214431 -4.270617 62 2844

2846 H1 8.718553 -2.675841 -2.653743 62 2844

2847 OH 9.409652 -0.879706 -3.307002 63 2844 2848

2848 HO 9.164492 -0.144483 -3.899181 64 2847

2849 C 7.430000 -0.630000 -1.340000 57 2842 2850 2851

2850 O 7.500000 -1.330000 -0.330000 59 2849

2851 N 7.630000 0.700000 -1.260000 122 2849 2852 2853

2852 H 7.640000 1.320000 -2.060000 125 2851

2853 CT 8.020000 1.010000 0.080000 123 2851 2854 2855 2870

2854 H1 8.460000 0.200000 0.660000 127 2853

2855 CT 9.164340 2.038574 0.035018 128 2853 2856 2857 2858

2856 HC 10.039404 1.532774 -0.376686 129 2855

2857 HC 9.433594 2.318788 1.054072 129 2855

2858 CA 8.932403 3.306857 -0.765009 130 2855 2859 2868

2859 CA 8.172877 4.365320 -0.227346 131 2858 2860 2861

2860 HA 7.705440 4.252549 0.740559 132 2859

2861 CA 8.019923 5.561529 -0.956437 133 2859 2862 2863

2862 HA 7.436530 6.379937 -0.566794 134 2861

2863 C 8.631760 5.698006 -2.221115 135 2861 2864 2866

2864 OH 8.494767 6.851714 -2.922360 136 2863 2865

2865 HO 8.951160 6.790519 -3.762686 137 2864

2866 CA 9.399517 4.641883 -2.753368 133 2863 2867 2868

2867 HA 9.873075 4.735334 -3.718733 134 2866

2868 CA 9.536998 3.444612 -2.029507 131 2858 2866 2869

2869 HA 10.110085 2.627066 -2.450853 132 2868

2870 C 6.870000 1.450000 0.940000 124 2853 2871 2872

2871 O 7.070000 1.980000 2.030000 126 2870

2872 N 5.610000 1.180000 0.520000 41 2870 2873 2874

2873 H 5.490000 0.780000 -0.410000 44 2872

2874 CT 4.470000 1.470000 1.350000 42 2872 2875 2876 2889

2875 H1 4.560000 2.480000 1.750000 46 2874

2876 CT 3.201309 1.529181 0.470935 47 2874 2877 2878 2882

2877 HC 3.046888 0.567589 -0.021294 48 2876

2878 CT 1.987530 1.860202 1.347577 51 2876 2879 2880 2881

2879 HC 1.071767 1.817312 0.759550 52 2878

2880 HC 1.900802 1.147702 2.161820 52 2878

2881 HC 2.101536 2.857964 1.769705 52 2878

2882 CT 3.381162 2.654005 -0.589664 49 2876 2883 2884 2885

2883 HC 4.300525 2.498222 -1.152338 50 2882

2884 HC 3.471947 3.616838 -0.084910 50 2882

2885 CT 2.266826 2.755882 -1.629427 53 2882 2886 2887 2888

2886 HC 1.327583 3.034394 -1.153964 54 2885

2887 HC 2.526138 3.520729 -2.362089 54 2885

2888 HC 2.166199 1.800479 -2.142269 54 2885

2889 C 4.450000 0.510000 2.500000 43 2874 2890 2891

2890 O 4.280000 0.880000 3.660000 45 2889

2891 N 4.660000 -0.790000 2.200000 65 2889 2892 2893

2892 H 4.830000 -1.030000 1.230000 68 2891

2893 CT 4.610000 -1.820000 3.190000 66 2891 2894 2895 2903

2894 H1 3.650000 -1.780000 3.710000 70 2893

2895 CT 4.707230 -3.222264 2.568955 71 2893 2896 2897 2901

2896 H1 3.967871 -3.272790 1.774166 72 2895

2897 CT 6.047116 -3.642694 1.969830 75 2895 2898 2899 2900

2898 HC 5.935560 -4.619724 1.497590 76 2897

2899 HC 6.351538 -2.930027 1.208118 76 2897

2900 HC 6.817409 -3.715545 2.737038 76 2897

2901 OH 4.376762 -4.177786 3.543482 73 2895 2902

2902 HO 5.071545 -4.195195 4.210209 74 2901

2903 C 5.700000 -1.600000 4.190000 67 2893 2904 2905

2904 O 5.500000 -1.830000 5.380000 69 2903

2905 N 6.890000 -1.160000 3.730000 27 2903 2906 2907

2906 H 7.020000 -1.030000 2.740000 30 2905

2907 CT 7.960000 -0.970000 4.660000 28 2905 2908 2909 2922

2908 H1 8.090000 -1.900000 5.220000 32 2907

2909 CT 9.280000 -0.580000 3.980000 33 2907 2910 2911 2912

2910 HC 9.150000 0.360000 3.440000 34 2909

2911 HC 9.520000 -1.350000 3.250000 34 2909

2912 CT 10.490000 -0.450000 4.940000 35 2909 2913 2914 2918

2913 HC 11.370000 -0.330000 4.310000 36 2912

2914 CT 10.410000 0.800000 5.830000 37 2912 2915 2916 2917

2915 HC 11.410000 0.970000 6.230000 38 2914

2916 HC 10.130000 1.670000 5.250000 38 2914

2917 HC 9.800000 0.680000 6.710000 38 2914

2918 CT 10.660000 -1.740000 5.750000 39 2912 2919 2920 2921

2919 HC 11.580000 -1.670000 6.340000 40 2918

2920 HC 9.840000 -1.880000 6.450000 40 2918

2921 HC 10.740000 -2.600000 5.080000 40 2918

2922 C 7.560000 0.110000 5.630000 29 2907 2923 2924

2923 O 7.660000 -0.070000 6.840000 31 2922

2924 N 7.070000 1.260000 5.120000 7 2922 2925 2926

2925 H 6.940000 1.370000 4.120000 10 2924

2926 CT 6.690000 2.300000 6.040000 8 2924 2927 2928 2932

2927 H1 7.450000 2.410000 6.820000 12 2926

2928 CT 6.510000 3.660000 5.330000 13 2926 2929 2930 2931

2929 HC 6.260000 4.440000 6.050000 14 2928

2930 HC 7.440000 3.930000 4.830000 14 2928

2931 HC 5.710000 3.580000 4.590000 14 2928

2932 C 5.390000 1.960000 6.700000 9 2926 2933 2934

2933 O 4.700000 2.810000 7.250000 11 2932

2934 N 5.070000 0.670000 6.780000 138 2932 2935 2936

2935 H 5.650000 -0.040000 6.360000 141 2934

2936 CT 3.840000 0.280000 7.350000 139 2934 2937 2938 2956

2937 H1 3.110000 1.090000 7.420000 143 2936

2938 CT 3.057751 -0.748328 6.514281 144 2936 2939 2940 2941

2939 HC 3.479160 -1.744636 6.631050 145 2938

2940 HC 3.130228 -0.465677 5.467522 145 2938

2941 C* 1.593845 -0.853001 6.819510 146 2938 2942 2955

2942 CW 0.963204 -1.930831 7.343797 147 2941 2943 2944

2943 H4 1.452540 -2.847912 7.655706 148 2942

2944 NA -0.403613 -1.709646 7.341356 150 2942 2945 2946

2945 H -1.090191 -2.377420 7.672225 151 2944

2946 CN -0.713905 -0.485918 6.789379 152 2944 2947 2955

2947 CA -1.924827 0.140741 6.465039 155 2946 2948 2949

2948 HA -2.855285 -0.391088 6.604411 156 2947

2949 CA -1.898227 1.446884 5.949164 159 2947 2950 2951

2950 HA -2.813662 1.937299 5.655738 160 2949

2951 CA -0.671365 2.112044 5.782131 157 2949 2952 2953

2952 HA -0.662244 3.121248 5.397679 158 2951

2953 CA 0.541405 1.447726 6.044599 153 2951 2954 2955

2954 HA 1.486829 1.933623 5.846066 154 2953

2955 CB 0.549375 0.125512 6.539818 149 2941 2946 2953

2956 C 4.120000 -0.260000 8.680000 140 2936 2957 2958

2957 O 3.600000 0.220000 9.690000 142 2956

2958 N 4.980000 -1.290000 8.730000 122 2956 2959 2960

2959 H 5.440000 -1.720000 7.930000 125 2958

2960 CT 4.970000 -1.780000 10.060000 123 2958 2961 2962 2977

2961 H1 4.030000 -1.650000 10.590000 127 2960

2962 CT 5.070000 -3.310000 10.180000 128 2960 2963 2964 2965

2963 HC 4.350000 -3.810000 9.540000 129 2962

2964 HC 4.850000 -3.610000 11.210000 129 2962

2965 CA 6.430000 -3.780000 9.810000 130 2962 2966 2975

2966 CA 6.750000 -3.990000 8.490000 131 2965 2967 2968

2967 HA 6.010000 -3.840000 7.710000 132 2966

2968 CA 8.010000 -4.420000 8.150000 133 2966 2969 2970

2969 HA 8.260000 -4.590000 7.120000 134 2968

2970 C 8.950000 -4.640000 9.140000 135 2968 2971 2973

2971 OH 10.240000 -5.080000 8.790000 136 2970 2972

2972 HO 10.300000 -5.280000 7.860000 137 2971

2973 CA 8.630000 -4.430000 10.460000 133 2970 2974 2975

2974 HA 9.370000 -4.620000 11.220000 134 2973

2975 CA 7.370000 -3.990000 10.790000 131 2965 2973 2976

2976 HA 7.120000 -3.890000 11.840000 132 2975

2977 C 6.040000 -1.120000 10.850000 124 2960 2978 2979

2978 O 6.470000 -1.640000 11.880000 126 2977

2979 N 6.460000 0.100000 10.460000 27 2977 2980 2981

2980 H 6.230000 0.450000 9.550000 30 2979

2981 CT 7.390000 0.730000 11.350000 28 2979 2982 2983 2996

2982 H1 7.560000 0.210000 12.290000 32 2981

2983 CT 8.760000 1.050000 10.770000 33 2981 2984 2985 2986

2984 HC 9.270000 1.750000 11.430000 34 2983

2985 HC 8.660000 1.520000 9.790000 34 2983

2986 CT 9.620000 -0.200000 10.670000 35 2983 2987 2988 2992

2987 HC 9.490000 -0.770000 11.590000 36 2986

2988 CT 9.170000 -1.120000 9.530000 37 2986 2989 2990 2991

2989 HC 9.850000 -1.970000 9.490000 38 2988

2990 HC 8.180000 -1.530000 9.660000 38 2988

2991 HC 9.240000 -0.590000 8.590000 38 2988

2992 CT 11.100000 0.180000 10.640000 39 2986 2993 2994 2995

2993 HC 11.700000 -0.730000 10.590000 40 2992

2994 HC 11.310000 0.800000 9.770000 40 2992

2995 HC 11.360000 0.720000 11.550000 40 2992

2996 C 6.790000 2.020000 11.760000 29 2981 2997 2998

2997 O 7.250000 2.660000 12.700000 31 2996

2998 N 5.700000 2.390000 11.090000 15 2996 2999 3000

2999 H 5.290000 1.740000 10.440000 18 2998

3000 CT 5.020000 3.610000 11.420000 16 2998 3001 3002 3012

3001 H1 5.760000 4.280000 11.810000 20 3000

3002 CT 4.370000 4.330000 10.270000 21 3000 3003 3004 3008

3003 HC 3.940000 5.280000 10.600000 22 3002

3004 CT 5.450000 4.750000 9.270000 23 3002 3005 3006 3007

3005 HC 4.990000 5.210000 8.400000 24 3004

3006 HC 6.120000 5.470000 9.740000 24 3004

3007 HC 6.040000 3.890000 8.960000 24 3004

3008 CT 3.230000 3.450000 9.700000 25 3002 3009 3010 3011

3009 HC 2.830000 3.950000 8.820000 26 3008

3010 HC 3.640000 2.510000 9.370000 26 3008

3011 HC 2.390000 3.250000 10.350000 26 3008

3012 C 3.970000 3.270000 12.420000 17 3000 3013 3014

3013 O 4.070000 2.230000 13.070000 19 3012

3014 N 2.940000 4.130000 12.510000 287 3012 3015 3016

3015 H 2.990000 4.960000 11.930000 290 3014

3016 CT 1.980000 4.130000 13.570000 288 3014 3017 3018 3036

3017 H1 2.460000 4.430000 14.510000 292 3016

3018 CT 0.810000 5.100000 13.220000 293 3016 3019 3020 3021

3019 HC 0.210000 4.680000 12.410000 294 3018

3020 HC 1.280000 6.010000 12.830000 294 3018

3021 CT -0.120000 5.520000 14.370000 295 3018 3022 3023 3024

3022 HC 0.470000 6.050000 15.120000 296 3021

3023 HC -0.540000 4.630000 14.840000 296 3021

3024 CT -1.270000 6.430000 13.930000 297 3021 3025 3026 3027

3025 H1 -1.790000 6.810000 14.800000 298 3024

3026 H1 -1.880000 5.820000 13.280000 298 3024

3027 N2 -0.740000 7.550000 13.080000 299 3024 3028 3029

3028 H 0.200000 7.870000 13.230000 300 3027

3029 CA -1.550000 8.080000 12.120000 301 3027 3030 3033

3030 N2 -2.830000 7.610000 11.970000 302 3029 3031 3032

3031 H -3.430000 8.250000 11.490000 303 3030

3032 H -3.150000 7.070000 12.750000 303 3030

3033 N2 -1.100000 9.060000 11.280000 302 3029 3034 3035

3034 H -1.620000 9.610000 10.630000 303 3033

3035 H -0.120000 9.360000 11.400000 303 3033

3036 C 1.450000 2.720000 13.720000 289 3016 3037 3038

3037 O 1.610000 2.120000 14.790000 291 3036

3038 N 0.920000 2.130000 12.620000 161 3036 3039 3040

3039 H 0.970000 2.590000 11.730000 164 3038

3040 CT 0.420000 0.790000 12.770000 162 3038 3041 3042 3054

3041 H1 0.060000 0.810000 13.800000 166 3040

3042 CT -0.800000 0.290000 11.990000 167 3040 3043 3044 3045

3043 HC -0.580000 -0.630000 11.440000 168 3042

3044 HC -1.170000 1.050000 11.300000 168 3042

3045 CC -1.800000 0.020000 13.080000 169 3042 3046 3052

3046 NA -2.540000 1.000000 13.710000 170 3045 3047 3048

3047 H -2.610000 1.960000 13.420000 171 3046

3048 CR -3.190000 0.390000 14.730000 174 3046 3049 3050

3049 H5 -4.030000 0.830000 15.260000 175 3048

3050 NA -2.920000 -0.900000 14.800000 176 3048 3051 3052

3051 H -3.520000 -1.660000 15.140000 177 3050

3052 CW -2.050000 -1.130000 13.760000 172 3045 3050 3053

3053 H4 -1.620000 -2.100000 13.520000 173 3052

3054 C 1.420000 -0.280000 13.010000 163 3040 3055 3056

3055 O 1.030000 -1.360000 13.440000 165 3054

3056 N 2.710000 -0.080000 12.680000 1 3054 3057 3058

3057 H 3.040000 0.830000 12.390000 4 3056

3058 CT 3.680000 -1.050000 13.100000 2 3056 3059 3060 3061

3059 H1 4.560000 -0.810000 12.570000 6 3058

3060 H1 3.360000 -2.090000 12.990000 6 3058

3061 C 3.960000 -0.680000 14.530000 3 3058 3062 3063

3062 O 3.030000 -0.450000 15.300000 5 3061

3063 N 5.230000 -0.630000 14.980000 107 3061 3064 3065

3064 H 6.070000 -0.760000 14.430000 110 3063

3065 CT 5.200000 -0.150000 16.330000 108 3063 3066 3067 3081

3066 H1 4.250000 0.080000 16.800000 112 3065

3067 CT 5.610000 -1.140000 17.450000 113 3065 3068 3069 3070

3068 HC 4.950000 -2.010000 17.390000 114 3067

3069 HC 5.420000 -0.690000 18.420000 114 3067

3070 CA 7.020000 -1.610000 17.400000 115 3067 3071 3079

3071 CA 8.000000 -0.890000 18.030000 116 3070 3072 3073

3072 HA 7.800000 0.070000 18.470000 117 3071

3073 CA 9.300000 -1.330000 18.010000 118 3071 3074 3075

3074 HA 10.080000 -0.740000 18.470000 119 3073

3075 CA 9.620000 -2.500000 17.350000 120 3073 3076 3077

3076 HA 10.650000 -2.840000 17.310000 121 3075

3077 CA 8.630000 -3.230000 16.740000 118 3075 3078 3079

3078 HA 8.880000 -4.150000 16.220000 119 3077

3079 CA 7.330000 -2.790000 16.770000 116 3070 3077 3080

3080 HA 6.560000 -3.370000 16.280000 117 3079

3081 C 5.870000 1.190000 16.460000 109 3065 3082 3083

3082 O 7.010000 1.410000 16.070000 111 3081

3083 N 5.070000 2.090000 17.050000 138 3081 3084 3085

3084 H 4.160000 1.710000 17.240000 141 3083

3085 CT 5.240000 3.470000 17.400000 139 3083 3086 3087 3105

3086 H1 4.250000 3.920000 17.350000 143 3085

3087 CT 5.730000 3.660000 18.850000 144 3085 3088 3089 3090

3088 HC 5.870000 4.720000 19.040000 145 3087

3089 HC 6.700000 3.170000 18.970000 145 3087

3090 C* 4.750000 3.120000 19.870000 146 3087 3091 3104

3091 CW 3.630000 3.720000 20.370000 147 3090 3092 3093

3092 H4 3.340000 4.730000 20.110000 148 3091

3093 NA 3.010000 2.890000 21.280000 150 3091 3094 3095

3094 H 2.450000 3.250000 22.040000 151 3093

3095 CN 3.760000 1.730000 21.390000 152 3093 3096 3104

3096 CA 3.570000 0.620000 22.160000 155 3095 3097 3098

3097 HA 2.750000 0.540000 22.860000 156 3096

3098 CA 4.500000 -0.390000 22.040000 159 3096 3099 3100

3099 HA 4.410000 -1.280000 22.640000 160 3098

3100 CA 5.580000 -0.280000 21.180000 157 3098 3101 3102

3101 HA 6.310000 -1.080000 21.130000 158 3100

3102 CA 5.770000 0.840000 20.410000 153 3100 3103 3104

3103 HA 6.630000 0.980000 19.780000 154 3102

3104 CB 4.850000 1.840000 20.520000 149 3090 3095 3102

3105 C 6.110000 4.270000 16.480000 140 3085 3106 3107

3106 O 7.270000 3.950000 16.230000 142 3105

3107 N 5.500000 5.370000 15.960000 55 3105 3108 3109

3108 H 4.570000 5.600000 16.260000 58 3107

3109 CT 6.090000 6.350000 15.080000 56 3107 3110 3111 3116

3110 H1 6.970000 6.780000 15.560000 60 3109

3111 CT 6.490000 5.800000 13.700000 61 3109 3112 3113 3114

3112 H1 6.750000 6.600000 13.000000 62 3111

3113 H1 5.610000 5.270000 13.360000 62 3111

3114 OH 7.610000 4.940000 13.830000 63 3111 3115

3115 HO 7.390000 4.000000 13.710000 64 3114

3116 C 5.060000 7.440000 14.880000 57 3109 3117 3118

3117 O 4.050000 7.470000 15.580000 59 3116

3118 N 5.300000 8.390000 13.940000 107 3116 3119 3120

3119 H 6.150000 8.350000 13.410000 110 3118

3120 CT 4.390000 9.500000 13.730000 108 3118 3121 3122 3136

3121 H1 3.510000 9.000000 13.990000 112 3120

3122 CT 4.540000 10.660000 14.720000 113 3120 3123 3124 3125

3123 HC 5.130000 11.530000 14.640000 114 3122

3124 HC 5.320000 10.250000 15.360000 114 3122

3125 CA 3.400000 10.600000 15.680000 115 3122 3126 3134

3126 CA 2.160000 11.010000 15.240000 116 3125 3127 3128

3127 HA 2.100000 11.560000 14.320000 117 3126

3128 CA 1.080000 10.990000 16.070000 118 3126 3129 3130

3129 HA 0.130000 11.380000 15.730000 119 3128

3130 CA 1.230000 10.550000 17.370000 120 3128 3131 3132

3131 HA 0.410000 10.610000 18.050000 121 3130

3132 CA 2.460000 10.150000 17.820000 118 3130 3133 3134

3133 HA 2.590000 9.880000 18.860000 119 3132

3134 CA 3.550000 10.180000 16.980000 116 3125 3132 3135

3135 HA 4.510000 9.880000 17.370000 117 3134

3136 C 4.480000 10.150000 12.390000 109 3120 3137 3138

3137 O 4.480000 9.510000 11.330000 111 3136

3138 N 4.520000 11.490000 12.440000 232 3136 3139 3140

3139 H 4.420000 11.990000 13.320000 235 3138

3140 CT 4.470000 12.370000 11.310000 233 3138 3141 3142 3151

3141 H1 3.540000 12.150000 10.820000 237 3140

3142 CT 4.440000 13.870000 11.670000 238 3140 3143 3144 3145

3143 HC 3.520000 14.130000 12.190000 239 3142

3144 HC 4.390000 14.410000 10.720000 239 3142

3145 CT 5.680000 14.370000 12.400000 240 3142 3146 3147 3148

3146 HC 5.710000 15.380000 12.150000 241 3145

3147 HC 6.440000 13.710000 12.040000 241 3145

3148 C 5.480000 14.200000 13.900000 242 3145 3149 3150

3149 O2 4.480000 13.540000 14.300000 243 3148

3150 O2 6.310000 14.750000 14.670000 243 3148

3151 C 5.620000 12.090000 10.410000 234 3140 3152 3153

3152 O 5.540000 12.370000 9.210000 236 3151

3153 N 6.730000 11.560000 10.950000 65 3151 3154 3155

3154 H 6.760000 11.450000 11.950000 68 3153

3155 CT 7.810000 11.230000 10.080000 66 3153 3156 3157 3165

3156 H1 8.130000 12.120000 9.530000 70 3155

3157 CT 9.010000 10.630000 10.780000 71 3155 3158 3159 3163

3158 H1 9.340000 11.320000 11.560000 72 3157

3159 CT 8.610000 9.300000 11.430000 75 3157 3160 3161 3162

3160 HC 9.420000 9.000000 12.100000 76 3159

3161 HC 7.710000 9.400000 12.040000 76 3159

3162 HC 8.520000 8.500000 10.700000 76 3159

3163 OH 10.070000 10.430000 9.860000 73 3157 3164

3164 HO 10.830000 10.140000 10.370000 74 3163

3165 C 7.270000 10.230000 9.100000 67 3155 3166 3167

3166 O 7.570000 10.300000 7.910000 69 3165

3167 N 6.440000 9.290000 9.590000 1 3165 3168 3169

3168 H 6.110000 9.370000 10.540000 4 3167

3169 CT 5.820000 8.310000 8.740000 2 3167 3170 3171 3172

3170 H1 5.180000 7.650000 9.330000 6 3169

3171 H1 6.570000 7.730000 8.200000 6 3169

3172 C 4.970000 9.070000 7.780000 3 3169 3173 3174

3173 O 4.810000 8.720000 6.620000 5 3172

3174 N 4.380000 10.170000 8.280000 232 3172 3175 3176

3175 H 4.470000 10.370000 9.270000 235 3174

3176 CT 3.530000 10.970000 7.470000 233 3174 3177 3178 3187

3177 H1 2.810000 10.300000 7.030000 237 3176

3178 CT 2.970000 12.170000 8.230000 238 3176 3179 3180 3181

3179 HC 3.640000 13.020000 8.270000 239 3178

3180 HC 2.650000 11.850000 9.200000 239 3178

3181 CT 1.730000 12.750000 7.560000 240 3178 3182 3183 3184

3182 HC 1.890000 12.900000 6.490000 241 3181

3183 HC 1.480000 13.710000 8.020000 241 3181

3184 C 0.620000 11.750000 7.800000 242 3181 3185 3186

3185 O2 0.740000 10.600000 7.300000 243 3184

3186 O2 -0.350000 12.110000 8.510000 243 3184

3187 C 4.340000 11.510000 6.340000 234 3176 3188 3189

3188 O 3.880000 11.570000 5.210000 236 3187

3189 N 5.600000 11.920000 6.630000 27 3187 3190 3191

3190 H 5.910000 11.890000 7.590000 30 3189

3191 CT 6.430000 12.490000 5.600000 28 3189 3192 3193 3206

3192 H1 5.930000 13.360000 5.170000 32 3191

3193 CT 7.820000 12.900000 6.140000 33 3191 3194 3195 3196

3194 HC 8.390000 12.010000 6.410000 34 3193

3195 HC 7.660000 13.480000 7.050000 34 3193

3196 CT 8.720000 13.740000 5.200000 35 3193 3197 3198 3202

3197 HC 8.060000 14.490000 5.040000 36 3196

3198 CT 10.050000 14.060000 5.890000 37 3196 3199 3200 3201

3199 HC 10.640000 14.690000 5.230000 38 3198

3200 HC 9.850000 14.570000 6.830000 38 3198

3201 HC 10.590000 13.140000 6.080000 38 3198

3202 CT 8.940000 13.120000 3.810000 39 3196 3203 3204 3205

3203 HC 9.820000 13.580000 3.360000 40 3202

3204 HC 9.120000 12.050000 3.870000 40 3202

3205 HC 8.130000 13.400000 3.140000 40 3202

3206 C 6.630000 11.460000 4.540000 29 3191 3207 3208

3207 O 6.520000 11.760000 3.360000 31 3206

3208 N 6.920000 10.210000 4.950000 161 3206 3209 3210

3209 H 6.970000 10.070000 5.960000 164 3208

3210 CT 7.200000 9.170000 3.980000 162 3208 3211 3212 3224

3211 H1 7.990000 9.520000 3.310000 166 3210

3212 CT 7.620000 7.820000 4.600000 167 3210 3213 3214 3215

3213 HC 7.330000 7.000000 3.940000 168 3212

3214 HC 7.090000 7.670000 5.540000 168 3212

3215 CC 9.090000 7.700000 4.840000 169 3212 3216 3222

3216 NA 9.990000 7.350000 3.860000 170 3215 3217 3218

3217 H 9.770000 7.140000 2.890000 171 3216

3218 CR 11.220000 7.340000 4.440000 174 3216 3219 3220

3219 H5 12.140000 7.100000 3.910000 175 3218

3220 NA 11.170000 7.650000 5.730000 176 3218 3221 3222

3221 H 11.940000 7.730000 6.390000 177 3220

3222 CW 9.830000 7.880000 5.980000 172 3215 3220 3223

3223 H4 9.410000 8.190000 6.930000 173 3222

3224 C 6.000000 8.910000 3.130000 163 3210 3225 3226

3225 O 6.120000 8.810000 1.910000 165 3224

3226 N 4.810000 8.790000 3.730000 41 3224 3227 3228

3227 H 4.750000 8.900000 4.740000 44 3226

3228 CT 3.650000 8.470000 2.950000 42 3226 3229 3230 3243

3229 H1 3.800000 7.530000 2.410000 46 3228

3230 CT 2.451726 8.103714 3.845442 47 3228 3231 3232 3236

3231 HC 2.226690 8.932238 4.519160 48 3230

3232 CT 1.251146 7.795710 2.928197 51 3230 3233 3234 3235

3233 HC 0.499871 7.212773 3.453115 52 3232

3234 HC 0.802379 8.726657 2.595911 52 3232

3235 HC 1.573979 7.217549 2.060944 52 3232

3236 CT 2.796337 6.824821 4.662122 49 3230 3237 3238 3239

3237 HC 3.797505 6.904517 5.081023 50 3236

3238 HC 2.801776 5.959820 3.996651 50 3236

3239 CT 1.864205 6.530600 5.843076 53 3236 3240 3241 3242

3240 HC 2.246421 5.669269 6.392349 54 3239

3241 HC 1.837437 7.388936 6.515636 54 3239

3242 HC 0.860063 6.296520 5.493188 54 3239

3243 C 3.460000 9.580000 1.970000 43 3228 3244 3245

3244 O 3.120000 9.340000 0.810000 45 3243

3245 N 3.680000 10.830000 2.430000 7 3243 3246 3247

3246 H 3.950000 10.990000 3.400000 10 3245

3247 CT 3.470000 11.980000 1.600000 8 3245 3248 3249 3253

3248 H1 2.440000 11.990000 1.250000 12 3247

3249 CT 3.740000 13.300000 2.340000 13 3247 3250 3251 3252

3250 HC 3.530000 14.140000 1.680000 14 3249

3251 HC 3.090000 13.370000 3.210000 14 3249

3252 HC 4.770000 13.370000 2.670000 14 3249

3253 C 4.370000 11.940000 0.400000 9 3247 3254 3255

3254 O 3.930000 12.180000 -0.720000 11 3253

3255 N 5.660000 11.610000 0.600000 27 3253 3256 3257

3256 H 5.980000 11.510000 1.560000 30 3255

3257 CT 6.590000 11.610000 -0.490000 28 3255 3258 3259 3272

3258 H1 6.550000 12.580000 -0.980000 32 3257

3259 CT 8.040000 11.330000 -0.030000 33 3257 3260 3261 3262

3260 HC 8.080000 10.370000 0.490000 34 3259

3261 HC 8.310000 12.100000 0.700000 34 3259

3262 CT 9.110000 11.340000 -1.140000 35 3259 3263 3264 3268

3263 HC 10.080000 11.300000 -0.640000 36 3262

3264 CT 9.030000 10.100000 -2.050000 37 3262 3265 3266 3267

3265 HC 9.980000 10.040000 -2.580000 38 3264

3266 HC 8.950000 9.220000 -1.430000 38 3264

3267 HC 8.300000 10.130000 -2.840000 38 3264

3268 CT 9.060000 12.660000 -1.930000 39 3262 3269 3270 3271

3269 HC 9.880000 12.680000 -2.640000 40 3268

3270 HC 8.130000 12.750000 -2.490000 40 3268

3271 HC 9.170000 13.510000 -1.250000 40 3268

3272 C 6.190000 10.570000 -1.480000 29 3257 3273 3274

3273 O 6.180000 10.810000 -2.680000 31 3272

3274 N 5.916652 9.353603 -0.997108 1 3272 3275 3276

3275 H 5.970195 9.225640 0.008153 4 3274

3276 CT 5.498479 8.254878 -1.854233 2 3274 3277 3278 3279

3277 H1 5.296717 7.367448 -1.255798 6 3276

3278 H1 6.284395 8.031261 -2.573900 6 3276

3279 C 4.240000 8.630000 -2.620000 3 3276 3280 3281

3280 O 4.130000 8.350000 -3.810000 5 3279

3281 N 3.260000 9.260000 -1.940000 661 3279 3282 3283

3282 H 3.420000 9.460000 -0.960000 664 3281

3283 CT 2.000000 9.590000 -2.540000 662 3281 3284 3285 3295

3284 H1 1.510000 8.700000 -2.930000 666 3283

3285 CT 1.067053 10.200959 -1.495397 667 3283 3286 3287 3288

3286 HC 0.204150 10.639685 -2.000083 668 3285

3287 HC 1.602687 10.989777 -0.969454 668 3285

3288 CT 0.592402 9.162588 -0.472015 669 3285 3289 3290 3291

3289 HC 0.772245 9.554009 0.518111 670 3288

3290 HC 1.142968 8.226365 -0.573308 670 3288

3291 C -0.880382 8.872624 -0.577449 671 3288 3292 3293

3292 O -1.269503 7.970687 -1.305429 672 3291

3293 OH -1.721924 9.565688 0.225281 673 3291 3294

3294 HO -1.222563 10.064187 0.911531 674 3293

3295 C 2.250000 10.550000 -3.660000 663 3283 3296 3297

3296 O 1.670000 10.440000 -4.740000 665 3295

3297 N 3.150000 11.530000 -3.420000 107 3295 3298 3299

3298 H 3.600000 11.580000 -2.510000 110 3297

3299 CT 3.430000 12.570000 -4.370000 108 3297 3300 3301 3315

3300 H1 2.520000 13.110000 -4.610000 112 3299

3301 CT 4.490000 13.550000 -3.830000 113 3299 3302 3303 3304

3302 HC 5.430000 13.040000 -3.620000 114 3301

3303 HC 4.130000 13.990000 -2.900000 114 3301

3304 CA 4.710000 14.620000 -4.850000 115 3301 3305 3313

3305 CA 5.550000 14.410000 -5.920000 116 3304 3306 3307

3306 HA 6.110000 13.490000 -6.010000 117 3305

3307 CA 5.760000 15.400000 -6.850000 118 3305 3308 3309

3308 HA 6.440000 15.240000 -7.670000 119 3307

3309 CA 5.130000 16.610000 -6.720000 120 3307 3310 3311

3310 HA 5.310000 17.400000 -7.440000 121 3309

3311 CA 4.300000 16.830000 -5.650000 118 3309 3312 3313

3312 HA 3.800000 17.790000 -5.550000 119 3311

3313 CA 4.090000 15.840000 -4.720000 116 3304 3311 3314

3314 HA 3.410000 15.960000 -3.900000 117 3313

3315 C 3.980000 11.940000 -5.610000 109 3299 3316 3317

3316 O 3.510000 12.190000 -6.710000 111 3315

3317 N 5.000000 11.070000 -5.440000 77 3315 3318 3319

3318 H 5.360000 10.900000 -4.500000 80 3317

3319 CT 5.620000 10.470000 -6.580000 78 3317 3320 3321 3326

3320 H1 5.930000 11.210000 -7.210000 82 3319

3321 CT 6.840000 9.610000 -6.200000 83 3319 3322 3323 3324

3322 H1 6.530000 8.820000 -5.510000 84 3321

3323 H1 7.570000 10.240000 -5.690000 84 3321

3324 SH 7.620000 8.860000 -7.660000 85 3321 3325

3325 HS 8.590000 8.190000 -7.020000 86 3324

3326 C 4.650000 9.570000 -7.270000 79 3319 3327 3328

3327 O 4.500000 9.610000 -8.490000 81 3326

3328 N 3.940000 8.730000 -6.490000 7 3326 3329 3330

3329 H 4.090000 8.720000 -5.490000 10 3328

3330 CT 3.080000 7.750000 -7.080000 8 3328 3331 3332 3336

3331 H1 3.660000 7.400000 -7.940000 12 3330

3332 CT 2.788097 6.625442 -6.077341 13 3330 3333 3334 3335

3333 HC 2.203147 5.842839 -6.559223 14 3332

3334 HC 3.722669 6.197340 -5.715084 14 3332

3335 HC 2.219056 7.019981 -5.235113 14 3332

3336 C 1.870000 8.250000 -7.750000 9 3330 3337 3338

3337 O 1.290000 7.540000 -8.560000 11 3336

3338 N 1.390000 9.520000 -7.500000 271 3336 3339 3340

3339 H 1.930000 10.020000 -6.800000 274 3338

3340 CT 0.090000 10.210000 -8.040000 272 3338 3341 3342 3356

3341 H1 -0.450000 9.500000 -8.670000 276 3340

3342 CT -0.820520 10.496831 -6.853869 277 3340 3343 3344 3345

3343 HC -1.746938 10.939938 -7.222733 278 3342

3344 HC -0.343057 11.183086 -6.154488 278 3342

3345 CT -1.105378 9.132743 -6.182137 279 3342 3346 3347 3348

3346 HC -0.208193 8.697889 -5.741891 280 3345

3347 HC -1.489435 8.449344 -6.939732 280 3345

3348 CT -2.134584 9.275578 -5.083138 281 3345 3349 3350 3351

3349 HC -2.848195 9.991111 -5.458364 282 3348

3350 HC -1.674556 9.665339 -4.173623 282 3348

3351 CT -2.886490 7.993681 -4.817684 283 3348 3352 3353 3354

3352 HP -3.100062 7.518535 -5.764460 284 3351

3353 HP -3.831979 8.244455 -4.348431 284 3351

3354 N3 -2.152452 7.089809 -3.976105 285 3351 3355 3706

3355 H -2.107662 7.311987 -3.000885 286 3354

3356 C 0.450000 11.350000 -8.930000 273 3340 3357 3358

3357 O 0.110000 11.490000 -9.980000 275 3356

3358 N 1.200000 12.590000 -8.520000 15 3356 3359 3360

3359 H 1.530000 12.450000 -7.580000 18 3358

3360 CT 1.710000 13.800000 -9.060000 16 3358 3361 3362 3372

3361 H1 0.910000 14.390000 -9.500000 20 3360

3362 CT 2.460000 14.590000 -8.030000 21 3360 3363 3364 3368

3363 HC 3.340000 14.120000 -7.650000 22 3362

3364 CT 3.090000 15.820000 -8.700000 23 3362 3365 3366 3367

3365 HC 3.120000 16.670000 -8.030000 24 3364

3366 HC 4.100000 15.590000 -9.050000 24 3364

3367 HC 2.520000 16.130000 -9.580000 24 3364

3368 CT 1.510000 14.890000 -6.860000 25 3362 3369 3370 3371

3369 HC 1.870000 15.730000 -6.270000 26 3368

3370 HC 0.490000 15.060000 -7.190000 26 3368

3371 HC 1.470000 14.020000 -6.210000 26 3368

3372 C 2.710000 13.450000 -10.120000 17 3360 3373 3374

3373 O 2.560000 13.840000 -11.280000 19 3372

3374 N 3.740000 12.680000 -9.730000 15 3372 3375 3376

3375 H 3.770000 12.370000 -8.760000 18 3374

3376 CT 4.810000 12.340000 -10.620000 16 3374 3377 3378 3388

3377 H1 5.200000 13.250000 -11.080000 20 3376

3378 CT 5.930000 11.620000 -9.930000 21 3376 3379 3380 3384

3379 HC 5.800000 10.660000 -9.630000 22 3378

3380 CT 6.980000 11.200000 -10.970000 23 3378 3381 3382 3383

3381 HC 7.830000 10.760000 -10.450000 24 3380

3382 HC 6.610000 10.450000 -11.670000 24 3380

3383 HC 7.330000 12.070000 -11.530000 24 3380

3384 CT 6.490000 12.530000 -8.820000 25 3378 3385 3386 3387

3385 HC 7.330000 12.060000 -8.320000 26 3384

3386 HC 6.900000 13.420000 -9.300000 26 3384

3387 HC 5.710000 12.890000 -8.150000 26 3384

3388 C 4.330000 11.450000 -11.720000 17 3376 3389 3390

3389 O 4.610000 11.680000 -12.890000 19 3388

3390 N 3.560000 10.410000 -11.350000 27 3388 3391 3392

3391 H 3.450000 10.230000 -10.360000 30 3390

3392 CT 3.120000 9.440000 -12.300000 28 3390 3393 3394 3407

3393 H1 3.980000 9.010000 -12.810000 32 3392

3394 CT 2.374815 8.306009 -11.568249 33 3392 3395 3396 3397

3395 HC 1.360717 8.633856 -11.333451 34 3394

3396 HC 2.877303 8.088984 -10.624726 34 3394

3397 CT 2.322221 7.010151 -12.402067 35 3394 3398 3399 3403

3398 HC 2.137522 7.249499 -13.448735 36 3397

3399 CT 3.638371 6.246328 -12.272004 37 3397 3400 3401 3402

3400 HC 3.584150 5.307778 -12.822412 38 3399

3401 HC 4.451367 6.840535 -12.679029 38 3399

3402 HC 3.831645 6.017640 -11.225075 38 3399

3403 CT 1.213653 6.084287 -11.915473 39 3397 3404 3405 3406

3404 HC 1.363204 5.847632 -10.862312 40 3403

3405 HC 0.255115 6.576540 -12.058605 40 3403

3406 HC 1.219151 5.162097 -12.498243 40 3403

3407 C 2.240000 10.140000 -13.290000 29 3392 3408 3409

3408 O 2.330000 9.920000 -14.500000 31 3407

3409 N 1.350000 11.020000 -12.800000 258 3407 3410 3411

3410 H 1.290000 11.140000 -11.800000 261 3409

3411 CT 0.420000 11.720000 -13.630000 259 3409 3412 3413 3424

3412 H1 -0.090000 10.900000 -14.120000 263 3411

3413 CT -0.530000 12.620000 -12.810000 264 3411 3414 3415 3416

3414 HC 0.060000 13.310000 -12.220000 265 3413

3415 HC -1.100000 11.980000 -12.130000 265 3413

3416 CT -1.550000 13.390000 -13.660000 266 3413 3417 3418 3419

3417 H1 -2.150000 12.940000 -14.310000 267 3416

3418 H1 -0.980000 14.060000 -14.300000 267 3416

3419 S -2.670000 14.450000 -12.700000 268 3416 3420

3420 CT -1.400000 15.670000 -12.290000 269 3419 3421 3422 3423

3421 H1 -1.860000 16.490000 -11.750000 270 3420

3422 H1 -0.620000 15.230000 -11.670000 270 3420

3423 H1 -0.960000 16.070000 -13.200000 270 3420

3424 C 1.130000 12.610000 -14.590000 260 3411 3425 3426

3425 O 0.750000 12.700000 -15.760000 262 3424

3426 N 2.200000 13.290000 -14.130000 27 3424 3427 3428

3427 H 2.470000 13.210000 -13.160000 30 3426

3428 CT 2.880000 14.190000 -15.010000 28 3426 3429 3430 3443

3429 H1 2.180000 14.920000 -15.390000 32 3428

3430 CT 4.020000 14.970000 -14.310000 33 3428 3431 3432 3433

3431 HC 4.560000 15.540000 -15.070000 34 3430

3432 HC 4.710000 14.260000 -13.850000 34 3430

3433 CT 3.530000 15.950000 -13.230000 35 3430 3434 3435 3439

3434 HC 3.020000 15.510000 -12.450000 36 3433

3435 CT 4.710000 16.690000 -12.580000 37 3433 3436 3437 3438

3436 HC 4.340000 17.360000 -11.810000 38 3435

3437 HC 5.390000 15.970000 -12.120000 38 3435

3438 HC 5.240000 17.270000 -13.340000 38 3435

3439 CT 2.450000 16.900000 -13.770000 39 3433 3440 3441 3442

3440 HC 2.200000 17.630000 -13.000000 40 3439

3441 HC 2.840000 17.430000 -14.640000 40 3439

3442 HC 1.540000 16.360000 -14.020000 40 3439

3443 C 3.470000 13.420000 -16.140000 29 3428 3444 3445

3444 O 3.390000 13.860000 -17.290000 31 3443

3445 N 4.050000 12.250000 -15.860000 41 3443 3446 3447

3446 H 4.070000 11.910000 -14.900000 44 3445

3447 CT 4.670000 11.520000 -16.930000 42 3445 3448 3449 3462

3448 H1 5.360000 12.190000 -17.440000 46 3447

3449 CT 5.430000 10.290000 -16.490000 47 3447 3450 3451 3455

3450 HC 6.050000 10.550000 -15.630000 48 3449

3451 CT 4.450000 9.190000 -16.070000 51 3449 3452 3453 3454

3452 HC 4.990000 8.260000 -15.980000 52 3451

3453 HC 4.310000 9.310000 -15.020000 52 3451

3454 HC 3.650000 8.850000 -16.710000 52 3451

3455 CT 6.380000 9.850000 -17.610000 49 3449 3456 3457 3458

3456 HC 7.070000 10.660000 -17.830000 50 3455

3457 HC 5.810000 9.620000 -18.510000 50 3455

3458 CT 7.190000 8.600000 -17.230000 53 3455 3459 3460 3461

3459 HC 7.850000 8.430000 -18.040000 54 3458

3460 HC 7.760000 8.770000 -16.320000 54 3458

3461 HC 6.570000 7.710000 -17.140000 54 3458

3462 C 3.610000 11.120000 -17.910000 43 3447 3463 3464

3463 O 3.800000 11.250000 -19.110000 45 3462

3464 N 2.450000 10.650000 -17.420000 107 3462 3465 3466

3465 H 2.330000 10.520000 -16.420000 110 3464

3466 CT 1.460000 10.240000 -18.370000 108 3464 3467 3468 3482

3467 H1 1.920000 9.530000 -19.060000 112 3466

3468 CT 0.180000 9.600000 -17.800000 113 3466 3469 3470 3471

3469 HC -0.250000 10.260000 -17.060000 114 3468

3470 HC 0.400000 8.630000 -17.360000 114 3468

3471 CA -0.670000 9.450000 -19.020000 115 3468 3472 3480

3472 CA -0.370000 8.470000 -19.940000 116 3471 3473 3474

3473 HA 0.500000 7.840000 -19.810000 117 3472

3474 CA -1.130000 8.310000 -21.080000 118 3472 3475 3476

3475 HA -0.780000 7.720000 -21.910000 119 3474

3476 CA -2.200000 9.140000 -21.300000 120 3474 3477 3478

3477 HA -2.640000 9.100000 -22.290000 121 3476

3478 CA -2.500000 10.130000 -20.400000 118 3476 3479 3480

3479 HA -3.080000 10.950000 -20.710000 119 3478

3480 CA -1.750000 10.280000 -19.260000 116 3471 3478 3481

3481 HA -1.950000 11.120000 -18.620000 117 3480

3482 C 0.960000 11.390000 -19.170000 109 3466 3483 3484

3483 O 0.830000 11.290000 -20.390000 111 3482

3484 N 0.660000 12.530000 -18.510000 15 3482 3485 3486

3485 H 0.820000 12.570000 -17.510000 18 3484

3486 CT 0.020000 13.620000 -19.190000 16 3484 3487 3488 3498

3487 H1 -0.920000 13.260000 -19.610000 20 3486

3488 CT -0.250000 14.790000 -18.290000 21 3486 3489 3490 3494

3489 HC -0.850000 14.440000 -17.450000 22 3488

3490 CT 1.090000 15.330000 -17.770000 23 3488 3491 3492 3493

3491 HC 0.850000 16.160000 -17.110000 24 3490

3492 HC 1.380000 14.560000 -17.120000 24 3490

3493 HC 1.800000 15.760000 -18.460000 24 3490

3494 CT -1.080000 15.820000 -19.070000 25 3488 3495 3496 3497

3495 HC -1.690000 16.270000 -18.310000 26 3494

3496 HC -0.430000 16.490000 -19.630000 26 3494

3497 HC -1.770000 15.320000 -19.760000 26 3494

3498 C 0.870000 14.110000 -20.320000 17 3486 3499 3500

3499 O 0.360000 14.430000 -21.390000 19 3498

3500 N 2.200000 14.200000 -20.110000 220 3498 3501 3502

3501 H 2.580000 13.950000 -19.200000 223 3500

3502 CT 3.040000 14.710000 -21.150000 221 3500 3503 3504 3512

3503 H1 2.680000 15.700000 -21.450000 225 3502

3504 CT 4.520000 14.770000 -20.740000 226 3502 3505 3506 3507

3505 HC 4.960000 13.770000 -20.700000 227 3504

3506 HC 4.600000 15.250000 -19.770000 227 3504

3507 C 5.260000 15.630000 -21.760000 228 3504 3508 3509

3508 O 4.670000 16.090000 -22.740000 229 3507

3509 N 6.580000 15.840000 -21.540000 230 3507 3510 3511

3510 H 7.000000 16.380000 -22.280000 231 3509

3511 H 7.060000 15.490000 -20.740000 231 3509

3512 C 2.930000 13.780000 -22.320000 222 3502 3513 3514

3513 O 2.840000 14.200000 -23.470000 224 3512

3514 N 2.910000 12.470000 -22.030000 220 3512 3515 3516

3515 H 2.910000 12.190000 -21.060000 223 3514

3516 CT 2.850000 11.450000 -23.030000 221 3514 3517 3518 3526

3517 H1 3.650000 11.640000 -23.750000 225 3516

3518 CT 2.960000 10.030000 -22.450000 226 3516 3519 3520 3521

3519 HC 2.140000 9.810000 -21.780000 227 3518

3520 HC 3.900000 9.910000 -21.920000 227 3518

3521 C 2.900000 9.040000 -23.600000 228 3518 3522 3523

3522 O 2.120000 8.090000 -23.590000 229 3521

3523 N 3.740000 9.280000 -24.640000 230 3521 3524 3525

3524 H 3.510000 8.680000 -25.420000 231 3523

3525 H 4.250000 10.130000 -24.760000 231 3523

3526 C 1.540000 11.530000 -23.760000 222 3516 3527 3528

3527 O 1.490000 11.270000 -24.960000 224 3526

3528 N 0.450000 11.900000 -23.070000 55 3526 3529 3530

3529 H 0.530000 12.050000 -22.070000 58 3528

3530 CT -0.830000 11.900000 -23.720000 56 3528 3531 3532 3537

3531 H1 -1.050000 10.900000 -24.090000 60 3530

3532 CT -1.990000 12.370000 -22.800000 61 3530 3533 3534 3535

3533 H1 -1.830000 11.860000 -21.870000 62 3532

3534 H1 -2.960000 12.140000 -23.230000 62 3532

3535 OH -1.860000 13.750000 -22.520000 63 3532 3536

3536 HO -2.260000 13.890000 -21.650000 64 3535

3537 C -0.760000 12.840000 -24.860000 57 3530 3538 3539

3538 O -1.430000 12.650000 -25.880000 59 3537

3539 N 0.050000 13.890000 -24.720000 15 3537 3540 3541

3540 H 0.570000 14.000000 -23.850000 18 3539

3541 CT 0.210000 14.820000 -25.790000 16 3539 3542 3543 3553

3542 H1 -0.740000 15.240000 -26.090000 20 3541

3543 CT 1.170000 15.920000 -25.470000 21 3541 3544 3545 3549

3544 HC 2.150000 15.530000 -25.220000 22 3543

3545 CT 1.380000 16.760000 -26.740000 23 3543 3546 3547 3548

3546 HC 2.000000 17.620000 -26.490000 24 3545

3547 HC 1.920000 16.220000 -27.510000 24 3545

3548 HC 0.430000 17.120000 -27.130000 24 3545

3549 CT 0.630000 16.720000 -24.280000 25 3543 3550 3551 3552

3550 HC 1.330000 17.520000 -24.050000 26 3549

3551 HC -0.340000 17.150000 -24.520000 26 3549

3552 HC 0.550000 16.090000 -23.400000 26 3549

3553 C 0.790000 14.070000 -26.950000 17 3541 3554 3555

3554 O 0.330000 14.200000 -28.080000 19 3553

3555 N 1.800000 13.230000 -26.670000 232 3553 3556 3557

3556 H 2.100000 13.130000 -25.710000 235 3555

3557 CT 2.490000 12.500000 -27.690000 233 3555 3558 3559 3568

3558 H1 2.850000 13.190000 -28.450000 237 3557

3559 CT 3.640000 11.640000 -27.130000 238 3557 3560 3561 3562

3560 HC 4.050000 11.010000 -27.920000 239 3559

3561 HC 3.260000 10.940000 -26.410000 239 3559

3562 CT 4.760000 12.460000 -26.490000 240 3559 3563 3564 3565

3563 HC 5.440000 11.780000 -25.970000 241 3562

3564 HC 4.350000 13.160000 -25.760000 241 3562

3565 C 5.510000 13.190000 -27.590000 242 3562 3566 3567

3566 O2 5.120000 13.020000 -28.780000 243 3565

3567 O2 6.470000 13.930000 -27.260000 243 3565

3568 C 1.540000 11.570000 -28.370000 234 3557 3569 3570

3569 O 1.540000 11.450000 -29.600000 236 3568

3570 N 0.670000 10.900000 -27.590000 232 3568 3571 3572

3571 H 0.750000 11.020000 -26.590000 235 3570

3572 CT -0.230000 9.920000 -28.140000 233 3570 3573 3574 3583

3573 H1 0.330000 9.170000 -28.700000 237 3572

3574 CT -1.070000 9.220000 -27.050000 238 3572 3575 3576 3577

3575 HC -1.670000 9.970000 -26.530000 239 3574

3576 HC -0.360000 8.790000 -26.340000 239 3574

3577 CT -1.980000 8.110000 -27.570000 240 3574 3578 3579 3580

3578 HC -1.410000 7.390000 -28.170000 241 3577

3579 HC -2.760000 8.580000 -28.160000 241 3577

3580 C -2.640000 7.440000 -26.380000 242 3577 3581 3582

3581 O2 -2.400000 7.910000 -25.230000 243 3580

3582 O2 -3.380000 6.450000 -26.590000 243 3580

3583 C -1.170000 10.590000 -29.080000 234 3572 3584 3585

3584 O -1.490000 10.050000 -30.140000 236 3583

3585 N -1.650000 11.790000 -28.720000 7 3583 3586 3587

3586 H -1.300000 12.180000 -27.850000 10 3585

3587 CT -2.580000 12.470000 -29.570000 8 3585 3588 3589 3593

3588 H1 -3.440000 11.830000 -29.750000 12 3587

3589 CT -3.040000 13.820000 -28.990000 13 3587 3590 3591 3592

3590 HC -4.050000 14.050000 -29.310000 14 3589

3591 HC -2.900000 13.730000 -27.930000 14 3589

3592 HC -2.360000 14.620000 -29.300000 14 3589

3593 C -1.900000 12.750000 -30.870000 9 3587 3594 3595

3594 O -2.480000 12.590000 -31.940000 11 3593

3595 N -0.620000 13.160000 -30.800000 244 3593 3596 3597

3596 H -0.150000 13.220000 -29.900000 247 3595

3597 CT 0.100000 13.540000 -31.980000 245 3595 3598 3599 3610

3598 H1 -0.450000 14.330000 -32.500000 249 3597

3599 CT 1.530000 14.020000 -31.650000 250 3597 3600 3601 3602

3600 HC 2.120000 13.170000 -31.310000 251 3599

3601 HC 1.510000 14.720000 -30.810000 251 3599

3602 CT 2.270000 14.620000 -32.850000 252 3599 3603 3604 3605

3603 HC 2.150000 14.010000 -33.750000 253 3602

3604 HC 3.330000 14.690000 -32.610000 253 3602

3605 C 1.730000 16.020000 -33.080000 254 3602 3606 3607

3606 O 0.870000 16.500000 -32.340000 255 3605

3607 N 2.260000 16.710000 -34.130000 256 3605 3608 3609

3608 H 1.870000 17.620000 -34.270000 257 3607

3609 H 2.930000 16.280000 -34.740000 257 3607

3610 C 0.220000 12.370000 -32.900000 246 3597 3611 3612

3611 O 0.000000 12.500000 -34.110000 248 3610

3612 N 0.570000 11.190000 -32.370000 41 3610 3613 3614

3613 H 0.760000 11.160000 -31.370000 44 3612

3614 CT 0.790000 10.040000 -33.200000 42 3612 3615 3616 3629

3615 H1 1.540000 10.280000 -33.950000 46 3614

3616 CT 1.280000 8.850000 -32.430000 47 3614 3617 3618 3622

3617 HC 0.550000 8.610000 -31.650000 48 3616

3618 CT 1.350000 7.660000 -33.390000 51 3616 3619 3620 3621

3619 HC 1.520000 6.760000 -32.820000 52 3618

3620 HC 0.410000 7.420000 -33.880000 52 3618

3621 HC 2.120000 7.810000 -34.150000 52 3618

3622 CT 2.620000 9.170000 -31.740000 49 3616 3623 3624 3625

3623 HC 2.540000 10.020000 -31.080000 50 3622

3624 HC 3.350000 9.430000 -32.510000 50 3622

3625 CT 3.150000 7.980000 -30.940000 53 3622 3626 3627 3628

3626 HC 4.150000 8.390000 -30.790000 54 3625

3627 HC 2.340000 7.770000 -30.230000 54 3625

3628 HC 3.200000 7.310000 -31.730000 54 3625

3629 C -0.480000 9.640000 -33.880000 43 3614 3630 3631

3630 O -0.500000 9.390000 -35.080000 45 3629

3631 N -1.590000 9.570000 -33.120000 220 3629 3632 3633

3632 H -1.520000 9.790000 -32.130000 223 3631

3633 CT -2.820000 9.100000 -33.700000 221 3631 3634 3635 3643

3634 H1 -2.670000 8.120000 -34.160000 225 3633

3635 CT -3.970000 9.040000 -32.670000 226 3633 3636 3637 3638

3636 HC -4.900000 8.790000 -33.180000 227 3635

3637 HC -4.070000 10.000000 -32.160000 227 3635

3638 C -3.660000 7.930000 -31.680000 228 3635 3639 3640

3639 O -3.360000 6.800000 -32.070000 229 3638

3640 N -3.730000 8.250000 -30.360000 230 3638 3641 3642

3641 H -3.410000 7.490000 -29.770000 231 3640

3642 H -3.620000 9.210000 -30.100000 231 3640

3643 C -3.240000 10.040000 -34.780000 222 3633 3644 3645

3644 O -3.630000 9.610000 -35.860000 224 3643

3645 N -3.140000 11.350000 -34.520000 65 3643 3646 3647

3646 H -2.700000 11.670000 -33.670000 68 3645

3647 CT -3.600000 12.300000 -35.480000 66 3645 3648 3649 3657

3648 H1 -4.640000 12.110000 -35.720000 70 3647

3649 CT -3.440000 13.710000 -34.990000 71 3647 3650 3651 3655

3650 H1 -3.840000 14.410000 -35.720000 72 3649

3651 CT -4.210000 13.860000 -33.660000 75 3649 3652 3653 3654

3652 HC -3.740000 14.610000 -33.020000 76 3651

3653 HC -5.210000 14.220000 -33.910000 76 3651

3654 HC -4.400000 12.930000 -33.130000 76 3651

3655 OH -2.060000 14.000000 -34.790000 73 3649 3656

3656 HO -1.980000 14.870000 -34.400000 74 3655

3657 C -2.820000 12.140000 -36.730000 67 3647 3658 3659

3658 O -3.390000 12.020000 -37.810000 69 3657

3659 N -1.480000 12.100000 -36.630000 15 3657 3660 3661

3660 H -1.060000 12.220000 -35.710000 18 3659

3661 CT -0.690000 11.970000 -37.820000 16 3659 3662 3663 3673

3662 H1 -0.980000 12.750000 -38.520000 20 3661

3663 CT 0.790000 12.040000 -37.560000 21 3661 3664 3665 3669

3664 HC 1.020000 12.980000 -37.060000 22 3663

3665 CT 1.210000 10.880000 -36.640000 23 3663 3666 3667 3668

3666 HC 2.280000 11.000000 -36.460000 24 3665

3667 HC 0.770000 11.070000 -35.700000 24 3665

3668 HC 1.140000 9.880000 -37.050000 24 3665

3669 CT 1.520000 12.040000 -38.920000 25 3663 3670 3671 3672

3670 HC 2.590000 12.160000 -38.750000 26 3669

3671 HC 1.360000 11.100000 -39.450000 26 3669

3672 HC 1.160000 12.870000 -39.530000 26 3669

3673 C -1.010000 10.660000 -38.450000 17 3661 3674 3675

3674 O -1.200000 10.570000 -39.660000 19 3673

3675 N -1.100000 9.590000 -37.630000 531 3673 3676 3677

3676 H -1.160000 9.750000 -36.640000 534 3675

3677 CT -1.410000 8.300000 -38.160000 532 3675 3678 3679 3684

3678 H1 -0.800000 8.070000 -39.030000 536 3677

3679 CT -1.290000 7.180000 -37.110000 61 3677 3680 3681 3682

3680 H1 -2.040000 7.360000 -36.340000 62 3679

3681 H1 -0.300000 7.200000 -36.660000 62 3679

3682 OH -1.520000 5.910000 -37.730000 63 3679 3683

3683 HO -1.980000 5.350000 -37.100000 64 3682

3684 C -2.860000 8.350000 -38.620000 533 3677 3685 3686

3685 O2 -3.280000 7.540000 -39.030000 535 3684

3686 O2 -3.520000 9.400000 -38.500000 535 3684

3687 Cl- -11.930000 16.630000 15.030000 2015

3688 Cl- 0.230000 25.130000 -23.170000 2015

3689 Cl- 12.570000 16.590000 4.050000 2015

3690 Cl- -7.110000 11.490000 24.180000 2015

3691 Cl- 1.410000 9.420000 12.920000 2015

3692 C3R 0.241407 -2.254409 3.049076 2013 3693 3697 3707 3708

3693 C3R 0.633012 -3.032155 4.321895 2013 3692 3694 3712 3713

3694 C3R 0.523515 -4.537248 4.174820 2013 3693 3695 3714 3715

3695 C3R 1.470720 -5.003546 3.080031 2013 3694 3696 3716 3717

3696 C2R 1.501539 -4.130510 1.854470 2012 3695 3697 3709

3697 C2R 0.954082 -2.852398 1.813826 2012 3692 3696 3698

3698 C2R 1.024900 -2.177835 0.553500 2012 3697 3699 3718

3699 C2R 0.350117 -1.050747 0.098608 2012 3698 3700 3719

3700 C2R 0.434859 -0.550730 -1.227804 2012 3699 3701 3710

3701 C2R -0.345941 0.589011 -1.510099 2012 3700 3702 3720

3702 C2R -0.426965 1.323729 -2.719963 2012 3701 3703 3721

3703 C2R -1.009648 2.602076 -2.710363 2012 3702 3704 3722

3704 C2R -1.031655 3.540126 -3.772247 2012 3703 3705 3711

3705 C2R -1.507974 4.837065 -3.457181 2012 3704 3706 3723

3706 C2R -1.782213 5.850559 -4.356991 2012 3354 3705 3724

3707 C3R -1.289256 -2.317463 2.862635 2013 3692 3725 3726 3727

3708 C3R 0.669861 -0.800166 3.314893 2013 3692 3728 3729 3730

3709 C3R 2.176755 -4.806826 0.682687 2013 3696 3731 3732 3733

3710 C3R 1.318631 -1.204565 -2.265204 2013 3700 3734 3735 3736

3711 C3R -0.485797 3.196514 -5.133414 2013 3704 3737 3738 3739

3712 HR 0.012237 -2.676876 5.137180 2014 3693

3713 HR 1.657565 -2.780879 4.588197 2014 3693

3714 HR -0.497165 -4.826578 3.946816 2014 3694

3715 HR 0.787357 -5.028606 5.105097 2014 3694

3716 HR 1.250239 -6.021959 2.773188 2014 3695

3717 HR 2.489177 -5.040239 3.462371 2014 3695

3718 HR 1.663732 -2.629124 -0.173689 2014 3698

3719 HR -0.334937 -0.546073 0.745446 2014 3699

3720 HR -0.895811 1.000064 -0.679429 2014 3701

3721 HR 0.014079 0.943173 -3.620053 2014 3702

3722 HR -1.413716 2.938019 -1.768783 2014 3703

3723 HR -1.704724 5.047381 -2.418384 2014 3705

3724 HR -1.733521 5.690704 -5.416423 2014 3706

3725 HR -1.647188 -3.332082 2.735101 2014 3707

3726 HR -1.777807 -1.900959 3.738711 2014 3707

3727 HR -1.618950 -1.751638 1.999910 2014 3707

3728 HR 0.328133 -0.098947 2.567230 2014 3708

3729 HR 0.263292 -0.473629 4.265049 2014 3708

3730 HR 1.750889 -0.715682 3.378986 2014 3708

3731 HR 1.432953 -5.269461 0.038049 2014 3709

3732 HR 2.787266 -4.157214 0.072246 2014 3709

3733 HR 2.823898 -5.591694 1.051897 2014 3709

3734 HR 1.218157 -0.731600 -3.229712 2014 3710

3735 HR 2.366130 -1.144799 -1.984601 2014 3710

3736 HR 1.061265 -2.250915 -2.392795 2014 3710

3737 HR 0.589836 3.347859 -5.162949 2014 3711

3738 HR -0.670417 2.164268 -5.396440 2014 3711

3739 HR -0.914983 3.800845 -5.921463 2014 3711

3740 OT -2.250000 -1.450000 -8.890000 2001 3741 3742

3741 HT -1.980000 -2.000000 -8.150000 2002 3740

3742 HT -3.070000 -1.070000 -8.520000 2002 3740

3743 OT 0.950000 10.400000 10.380000 2001 3744 3745

3744 HT 1.440000 10.670000 11.170000 2002 3743

3745 HT 0.610000 11.210000 9.980000 2002 3743

3746 OT -1.610000 13.430000 4.090000 2001 3747 3748

3747 HT -1.030000 14.190000 4.190000 2002 3746

3748 HT -2.470000 13.850000 4.020000 2002 3746

3749 OT -4.320000 10.550000 2.550000 2001 3750 3751

3750 HT -4.410000 10.070000 1.710000 2002 3749

3751 HT -5.200000 10.920000 2.700000 2002 3749

3752 OT -3.660000 13.280000 -20.250000 2001 3753 3754

3753 HT -4.480000 12.790000 -20.080000 2002 3752

3754 HT -3.310000 13.410000 -19.370000 2002 3752

3755 OT -0.430000 11.080000 2.030000 2001 3756 3757

3756 HT 0.320000 10.830000 2.570000 2002 3755

3757 HT -0.580000 12.000000 2.300000 2002 3755

3758 OT -4.260000 7.280000 -11.650000 2001 3759 3760

3759 HT -4.880000 6.610000 -11.960000 2002 3758

3760 HT -3.400000 6.900000 -11.840000 2002 3758

3761 OT -3.810000 -9.600000 -11.890000 2001 3762 3763

3762 HT -3.180000 -9.470000 -11.180000 2002 3761

3763 HT -3.430000 -9.100000 -12.640000 2002 3761

3764 LAH -2.334728 8.934360 -5.012479 2999
